# Chromosome-level assemblies of two hexaploid bamboos, *Thyrsostachys oliveri* and *Thyrsostachys siamensis*, provide a foundation for functional and comparative genomics studies

**DOI:** 10.1093/gigascience/giaf142

**Published:** 2025-11-17

**Authors:** Chaiwat Naktang, Supaporn Khanbo, Poompat Phadphon, Sonicha U-thoomporn, Duangjai Sangsakru, Chutima Sonthirod, Pitchaporn Waiyamitra, Sarawood Sungkaew, Sithichoke Tangphatsornruang, Wirulda Pootakham

**Affiliations:** National Omics Center,National Center for Genetic Engineering and Biotechnology, National Science and Technology Development Technology, 111 Thailand Science Park, Pathum Thani 12120, Thailand; National Omics Center,National Center for Genetic Engineering and Biotechnology, National Science and Technology Development Technology, 111 Thailand Science Park, Pathum Thani 12120, Thailand; National Omics Center,National Center for Genetic Engineering and Biotechnology, National Science and Technology Development Technology, 111 Thailand Science Park, Pathum Thani 12120, Thailand; National Omics Center,National Center for Genetic Engineering and Biotechnology, National Science and Technology Development Technology, 111 Thailand Science Park, Pathum Thani 12120, Thailand; National Omics Center,National Center for Genetic Engineering and Biotechnology, National Science and Technology Development Technology, 111 Thailand Science Park, Pathum Thani 12120, Thailand; National Omics Center,National Center for Genetic Engineering and Biotechnology, National Science and Technology Development Technology, 111 Thailand Science Park, Pathum Thani 12120, Thailand; National Omics Center,National Center for Genetic Engineering and Biotechnology, National Science and Technology Development Technology, 111 Thailand Science Park, Pathum Thani 12120, Thailand; Department of Forest Biology, Faculty of Forestry, Kasetsart University, Bangkok 10900, Thailand; National Omics Center,National Center for Genetic Engineering and Biotechnology, National Science and Technology Development Technology, 111 Thailand Science Park, Pathum Thani 12120, Thailand; National Omics Center,National Center for Genetic Engineering and Biotechnology, National Science and Technology Development Technology, 111 Thailand Science Park, Pathum Thani 12120, Thailand

**Keywords:** *Thyrsostachys oliveri*, *Thyrsostachys siamensis*, chromosome-scale genome assembly, annotation, Hi-C, stLFR

## Abstract

**Background:**

Bamboo is an important nontimber forest resource with significant ecological and economic value. However, genomic resources for several bamboo lineages remain scarce.

**Results:**

We present the first chromosome-scale genome assemblies for 2 economically important Thai woody bamboos, *Thyrsostachys oliveri* and *Thyrsostachys siamensis*. Using single-tube long-fragment read (stLFR) sequencing integrated with chromosome conformation capture (Hi-C) scaffolding, we assembled 35 pseudochromosomes spanning 990 Mb (N50 = 22.18 Mb) for *T. oliveri* and 1.14 Gb (N50 = 19.45 Mb) for *T. siamensis*. The *T. oliveri* and *T. siamensis* genome assemblies contain 51,191 and 67,483 predicted genes, with repeat contents of 50.9% and 48.8%, respectively. BUSCO completeness scores reached 97.4% for *T. oliveri* and 95.2% for *T. siamensis*, indicating near-complete coverage of the conserved gene space. Comparative analyses revealed that *Thyrsostachys* formed a sister group to *Dendrocalamus*, and the 2 *Thyrsostachys* species diverged approximately 5.4 million years ago. Both species have undergone a recent whole-genome duplication event. Gene family analysis identified species-specific gene families associated with root development and carbohydrate metabolism in *T. oliveri* and histone binding and proteasome pathways in *T. siamensis*. Moreover, homoeolog suppression was more frequent than single-homoeolog dominance, and subgenome-specific expression biases differed between species.

**Conclusions:**

These genome assemblies fill a critical gap in bamboo genomics and provide a foundation for evolutionary, functional, and breeding research in hexaploid woody bamboos. They also serve as valuable reference genomes for comparative studies within and across bamboo species.

## Introduction

Bamboo is one of the most important nontimber forest resources that are widely distributed across the subtropical and tropical regions of Asia, Africa, and Latin America [1–3], covering roughly 31–35 million ha (≈0.8–1% of global forest area) [4, 5]. Bamboos constitute the subfamily Bambusoideae of Poaceae (Gramineae) and include about 1,670 species in 125 genera [3, 6, 7]. They are classified into 4 monophyletic lineages with distinct ploidy levels: herbaceous bamboos (2n = 20–24, diploid), temperate woody bamboos (2n = 46–48, tetraploid), neotropical woody bamboos (2n = 40–48, tetraploid), and palaeotropical woody bamboos (2n = 70–72, hexaploid) [8–11]. Among these, woody bamboos show a wide range in genome sizes, chromosome number, and ploidy [8]. Because of rapid growth and ease of propagation, bamboos provide substantial economic and ecological benefits. Their uses span food, medicine, bioenergy, building timber, furniture, and handicrafts, and they also help restore degraded landscapes and mitigate climate change impacts [12–14].

Bamboo plays a significant economic role in many developing countries, particularly in Asia [15]. In Thailand, roughly 69 species across 17 genera have been recorded [14, 16]. *Thyrsostachys* is a small bamboo genus native to Thailand and Myanmar, comprising only 2 species*: Thyrsostachys oliveri* (NCBI:txid338531) and *Thyrsostachys siamensis* (NCBI:txid338532). *T. oliveri* is a tropical clumping bamboo characterized by short rhizomes and straight and slender culms. These culms also have high branches with dense, small leaves, which give the entire bamboo cluster a slender and elegant appearance [17, 18]. Its culms are widely used locally for construction, furniture, and household products [17]. This bamboo is also cultivated as an ornamental plant [19]. *T. siamensis* is one of the most useful Thai bamboos, yielding wood and good edible shoots [19]. The species is characterized by its compact clumps, branching from upper mid-culm, and small leaves [19]. It grows rapidly, develops thicker culm walls, accumulates larger biomass, and supplies material for construction and handicrafts [14, 20]. Both species share a characteristic erect while differing in key traits such as culm wall thickness, branching patterns, and shoot productivity. Despite their ecological and economic significance, they lack genomic resources, leaving many key traits poorly understood at the molecular level.

Although reference genomes have been developed for multiple bamboo species ,including *Dendrocalamus brandisii* [21], *Dendrocalamus latiflorus* [22], *Phyllostachys edulis* [13, 23], *Bambusa odashimae* [24], *Raddia distichophylla* [25], *Phyllostachys violascens* [26], and others at various ploidy levels [27], genomic resources for *T. oliveri* or *T. siamensis* have not yet been established. The absence of reference genomes for *Thyrsostachys* now represents a critical gap. Therefore, there is an urgent need to obtain a reference genome for the genus. Generating chromosome-scale reference genomes for this genus will therefore provide essential resources for investigating lineage-specific adaptations, uncovering the evolutionary history, and enabling functional studies across multiple Bambusoideae lineages. These genomes will also help identify genes or proteins that control desirable traits, providing a foundation for trait-specific genetic modification programs in bamboos and offering new insights into the genetic basis of growth form, morphological diversity, and ecological adaptation. In addition, they will facilitate comparative genomics and transcriptomics with other woody bamboos and support conservation, breeding, and sustainable utilization efforts.

Here, we sequenced and assembled 2 Thai hexaploid species, *T. oliveri* and *T. siamensis*, using single-tube long-fragment reads (stLFR) technology [28] combined with chromosome conformation capture (Hi-C) scaffolding. The resulting chromosome-level assemblies provide crucial resources for investigating genomic organization and evolution in these species. Our work fills a critical gap in bamboo genomics and lays the groundwork for future studies aimed at genetic improvement, molecular breeding, and conservation of economically important bamboos.

## Methods

### Plant materials and DNA/RNA isolation

For genome sequencing, young leaf samples were collected from *T. oliveri* and *T. siamensis* plants grown at Kasetsart University, Bangkok Province, Thailand (13.8423°N, 100.5771°E). Immediately after collection, healthy leaves were flash-frozen in liquid nitrogen and stored at −80°C. High-molecular-weight (HMW) DNA was extracted using the Qiagen Genomic-tip 100/G following the manufacturer’s instructions. DNA quality and quantity were subsequently evaluated using the Pippin Pulse Electrophoresis System (Sage Science) and the Qubit 4 Fluorometer (RRID:SCR_018095) (Thermo Fisher Scientific), respectively, prior to library construction. For downstream annotation purposes, total RNA was extracted from leaf and root tissues of the same individual used for RNA sequencing (RNA-seq), following the protocol outlined in [29]. Briefly, RNA was isolated using a CTAB buffer and a 25:24:1 phenol/chloroform/isoamyl alcohol mixture, then precipitated overnight with a quarter volume of 8 M LiCl. The resulting RNA pellets were washed with 70% ethanol, air-dried, and resuspended in RNase-free water. RNA integrity was assessed using the Fragment Analyzer system (RRID:SCR_019417) (Agilent) before RNA-seq library construction. Representative photographs of *T. oliveri* and *T. siamensis* are shown in Supplementary Figs. S1–S2.

### Genome and transcriptome sequencing

A preliminary draft genome assembly was generated by first constructing a stLFR sequencing library. This library was prepared using 10 ng HMW DNA and the MGIEasy stLFR Library Prep Kit (MGI Tech), following the manufacturer’s protocol. Briefly, this process involved transposon-mediated insertion into the HMW DNA, incubation with clonally barcoded beads for random priming, and subsequent fragmentation into subfragments (<1 kb). Following adapter ligation and PCR amplification, the library was ready for sequencing. For transcriptome analysis, 200 ng total RNA was used to generate a library with the MGIEasy RNA Library Prep Kit v3.0 (MGI Tech), adhering to the manufacturer’s instructions. Both the stLFR and RNA libraries were then sequenced on the DNBSEQ-G400 (RRID:SCR_017980) platform using the MGISEQ-2000RS Sequencing Flow Cell v3.0 (MGI Tech).

### Hi-C library preparation and sequencing

To achieve chromosome-level scaffolding of the initial assembly, Biomarker Technologies utilized a chromosome conformation capture (Hi-C) technique. For Hi-C library preparation using *T. oliveri* tissue, the following procedure was employed. Briefly, chromatin from fresh tissue was crosslinked with formaldehyde, and the fixed chromatin was digested with the restriction endonuclease HindIII [30]. The resulting fragments were then treated to incorporate biotinylated nucleotides at their 5′ ends, followed by ligation to form chimeric junctions representing spatially proximate chromatin regions. After reversing the crosslinks, the DNA was purified and sheared into fragments ranging from 300 to 700 bp. Biotinylated fragments were then enriched using streptavidin beads. The purified Hi-C fragments were used to construct Illumina-compatible sequencing libraries, which were subsequently sequenced on the Illumina HiSeq X Ten (RRID:SCR_016385) (PE150).

### Genome assembly and Hi-C scaffolding

Preliminary draft assemblies for both genomes were generated from 150-bp paired-end stLFR sequencing data using stLFRdenovo v1.0.5 [31] with default settings. Subsequently, the initial draft assembly of *T. oliveri* was scaffolded to the chromosome level utilizing Hi-C data. This Hi-C scaffolding was conducted by Biomarker Technologies. The draft assembly and Hi-C reads were processed using HiRise v2.1.9 (RRID:SCR_017788), a pipeline optimized for proximity ligation data [32]. Hi-C reads were aligned to the draft assembly using BWA v0.7.17 (RRID:SCR_010910) [33] while data filtering and quality assessment were performed with HiC-Pro v2.10.0 (RRID:SCR_017643) [34]. The HiRise software analyzed Hi-C read pair distributions to detect and correct misjoins, identify prospective joins, and produce a chromosome-level assembly. For visualization, a genome-wide Hi-C contact matrix was generated with LACHESIS (RRID:SCR_017644) [35] and plotted in CGAP (Biomarker Technologies’ in-house Hi-C visualization software), which displays higher interaction intensities as darker colors. Manual inspection and limited adjustments were performed in CGAP; for example, when the interaction between positions 1 and 3 was stronger than that between positions 2 and 3 within a local block, the short interval between positions 1 and 2 was flipped to restore the expected near-diagonal *cis* signal (Supplementary Fig. S3A, B). *T. siamensis* was subsequently scaffolded using the *T. oliveri* chromosome-level assembly as a reference with the RagTag software v1.1.0 (RRID:SCR_027293) [36, 37]. Both *T. oliveri* and *T. siamensis* genome assemblies have been deposited at NCBI under accession numbers JAWCWW000000000 and JBEFOJ000000000, respectively. The raw stLFR reads for *T. oliveri* and *T. siamensis* are available in the NCBI SRA database under accession numbers SRR32915794 and SRR32915889, respectively. The transcriptome data for *T. oliveri* were submitted under SRR33015685 and SRR33015684. The transcriptome data for *T. siamensis* were submitted under SRR33015804 and SRR33015803.

### Genome size estimation

Genome size was estimated using 2 complementary approaches. First, the *k*-mer analysis was performed on raw stLFR reads using Jellyfish v2.2.10 (RRID:SCR_005491), and the resulting distributions were visualized with GenomeScope v2.0 (RRID:SCR_017014) [38, 39] (*k* = 21). Second, nuclear DNA content was estimated using flow cytometry. Fresh leaf tissues from *T. oliveri* and *T. siamensis* were processed following the protocol described in [40], using Galbraith’s buffer for nuclear isolation. Nuclei were stained with 50 µg/mL propidium iodide (Thermo Fisher Scientific). Maize (*Zea mays*) leaf sample was used as the reference standard for DNA content.

### Quality assessment of the genome assembly

To evaluate the quality of the final *T. oliveri* and *T. siamensis* assemblies, both short-read DNA (stLFR) and RNA-seq data were mapped to each respective assembly. Specifically, stLFR reads were aligned using BWA v0.7.17 [33], and RNA-seq reads were aligned using HISAT2 v2.2.0 (RRID:SCR_015530) [41]. Furthermore, assembly and proteome completeness was assessed using the BUSCO pipeline v5.4.4 (RRID:SCR_015008) [42], which examined the presence of conserved orthologous genes against the plant-specific Embryophyta OrthoDB database release 10 [43]. In addition, we computed the long terminal repeat (LTR) assembly index (LAI) with LTR_retriever v3.0 (RRID:SCR_017623) [44] and estimated base-level quality value (QV) with Merqury v1.3 [45] (RRID:SCR_022964).

### Repetitive sequence identification

Repetitive elements were identified through both *de novo* and homology-based approaches. First, we generated a *de novo* repeat library using RepeatModeler (v2.0.3) (RRID:SCR_015027) [46], which incorporates multiple algorithms (RECON, RepeatScout, and LtrHarvest/Ltr_retriever) to detect and characterize repeat boundaries. The resulting consensus sequences were aligned against the NCBI GenBank nonredundant protein database (nr) via BLASTX (e-value ≤ 1e^−6^) (RRID:SCR_004870) to confirm the absence of large, transposable element (non-TE) protein-coding families. For homology-based identification, the assembled genome was scanned with RepeatMasker v4.0.9_p2 (RRID:SCR_012954) [47] using the RepBase plant repeat database (RRID:SCR_021169) [48].

### Gene prediction and annotation

To annotate protein-coding sequences, we employed both Evidence Modeler (EVM) software version 1.1.1 (RRID:SCR_014659) [49] and the MAKER2 (RRID:SCR_005309) pipeline [50] to identify protein-coding genes in the masked genome assembly. These approaches combined homology-based, RNA-based, and *ab initio* predictions. Transcript-based gene prediction was performed using RNA-seq data derived from leaf and root tissues. Raw reads were assembled using Trinity v2.9.1 (RRID:SCR_013048) [51] and clustered at 95% identity with CD-HIT v4.8.1 (RRID:SCR_007105) [52, 53]. For each cluster, the longest open reading frame was identified and subsequently aligned to the assembled genome using PASA v2.5.3 (RRID:SCR_014656) [54] and GMAP v2020-09-12 (RRID:SCR_008992) [55]. To aid in gene annotation, protein sequences from related bamboo species, specifically *Dendrocalamus latiflorus* [56], *Dendrocalamus sinicus, Bonia amplexicaulis, Guadua angustifolia, Olyra latifolia*, and *Raddia guianensis* [57], were downloaded from publicly available databases and aligned to the genome assembly using AAT [58]. *Ab initio* gene predictions were generated with AUGUSTUS v3.2.1 (RRID:SCR_008417) [59], trained on data from the same reference species plus PASA transcript alignments. EVM was then used to integrate these lines of evidence—transcript, homology, and *ab initio*—assigning weights of 5 for PASA, 1 for GMAP, 0.5 for AAT, and 0.1 for AUGUSTUS.

In parallel, the MAKER2 pipeline was employed to merge *ab initio* predictions (using SNAP (RRID:SCR_007936) [60] and AUGUSTUS), protein homology, and transcript-based evidence. After an initial MAKER run with default parameters, the resulting gene models were used to retrain SNAP and AUGUSTUS, followed by a second MAKER iteration to refine predictions. Low-confidence models were removed or flagged for manual inspection, and the final consensus gene models were output in GFF format. GFF files exhibiting the highest BUSCO completeness scores were ultimately selected for downstream analyses, ensuring robust and high-quality gene annotations. All predicted genes were functionally annotated using OmicsBox v2.0.10 [61] (RRID:SCR_018930). Protein sequences were aligned against the GenBank’s nonredundant (NR) databases via BLASTP [62] with an E-value cutoff of 10^−5^. Gene Ontology (GO) terms were retrieved and assigned to the predicted gene models, while enzyme codes (EC) were extracted and mapped to KEGG pathway annotations. To annotate noncoding RNAs (ncRNAs), transfer RNAs (tRNAs) were predicted using tRNAscan-SE v2.0 (RRID:SCR_008637) [63], and ribosomal RNA (rRNA) sequences were identified by aligning known rRNA references from closely related species using BLAST. Other ncRNAs, such as microRNAs and small nuclear RNAs, were identified by searching against the Rfam v14.1 database (RRID:SCR_007891) [64] with Infernal v1.1.5 (RRID:SCR_011809) [65] using default parameters.

### Comparative genomics and phylogenetic analyses

To investigate the evolutionary relationships of *T. oliveri* and *T. siamensis* within the grass family, a comparative genomics study was conducted. OrthoFinder v2.5.5 (RRID:SCR_017118) [66] was used to identify orthologous groups among 10 grass species, including 9 bamboo species (*B. amplexicaulis, G. angustifolia, P. edulis* [Moso bamboo], *T. oliveri, T. siamensis, O. latifolia, R. guianensis, D. sinicus*, and *D. latiflorus*), other grasses (*Brachypodium distachyon* [a model grass species], and a cereal crop (*Oryza sativa* [rice]). A set of single-copy orthologous proteins was extracted, and these sequences were aligned and trimmed using MUSCLE v3.8 (RRID:SCR_011812) [67] and trimAl (RRID:SCR_017334) [68], respectively. Catsequences software [69] was used to concatenate the alignment blocks. The best substitution model for each block was evaluated using ModelTest-NG (RRID:SCR_026633) [70]. A maximum likelihood phylogenetic tree was constructed using RAxML-NG (RRID:SCR_022066) [71] with 1,000 bootstrap. *O. sativa* was designated as an outgroup.

The divergence time of the 11 species was estimated using BEAST v.2.7.7 (RRID:SCR_017307) [72]. Two independent MCMC tree searches were run for 10,000,000 generations, with a sampling frequency of 1,000 generations. The estimations were run under the JTT substitution model together with optimized relaxed clock model and Birth-Death tree prior. The Bambusoideae cf. Chusquea fossil (35–90 million years ago [Mya]; [73]) and the fossilized phytoliths and cuticle of Ehrhartoideae-Oryzeae (67–90 Mya; [74]) were used to calibrate the crown node of Bambusoideae and BEP clade, respectively, following [75]. The crown node of Bambuseae, estimated to be 28.24 (19.82–38.95) Mya by Zhang et al. [75], was included as a secondary calibration point. To assess the convergence of parameters, Tracer v1.7.1(RRID:SCR_019121) [76] was utilized to check the effective sample sizes (ESSs). ESSs of all parameters were above 200 after a burn-in period of 25%. The maximum clade credibility tree was generated by TreeAnnotator v2.7.7 [72].

Gene family expansions and contractions were identified using CAFE v5 (RRID:SCR_018924) [77], to detect significant changes in gene family size across the phylogeny (P < 0.05). This analysis employed a probabilistic model based on a time-calibrated phylogenetic tree and estimated gene birth/death rates (λ) using a maximum likelihood approach. Functionally, GO analyses were performed to annotate expanded and contracted gene families.

### The analysis of genome synteny

Collinearity analyses were performed using McscanX (RRID:SCR_022067) [78] to investigate syntenic relationships within the *T. oliveri* and *T. siamensis* genomes, as well as between *T. oliveri* and *T. siamensis, B. amplexicaulis, D. sinicus, D. latiflorus, O. latifolia, P. edulis*, and *O. sativa*. Putative paralogous gene pairs were identified by aligning *T. oliveri* amino acid sequences against themselves and those of *T. siamensis* using BLASTP with an E-value threshold of 10^−10^. Intragenic homeologous blocks were defined as regions containing 10 or more collinear or nearly collinear paralogous gene pairs, with a maximum of 6 intervening nonparalogous genes. The resulting intragenic homeologous blocks were visualized using CIRCOS v0.69.8 (RRID:SCR_011798) [79]. The synteny of the *Thyrsostachys* subgenome and *O. sativa* was examined using NGenomeSyn 1.41 [80] to identify similarities in their genomic structures. Ks for syntenic anchor pairs was estimated using the MCScanX script add_ka_and_ks_to_collinearity.pl. For the subgenome-level test, we repeated this pipeline on single-copy orthogroups from the 5 hexaploids (*Thyrsostachys, Melocanna baccifera, B. amplexicaulis, D. sinicus*), labeled sequences by A/B/C, and asked whether genes with the same label formed a monophyletic clade across species.

### Subgenome identification

To facilitate subgenome classification within the newly assembled *T. oliveri* and *T. siamensis* genomes, the established subgenome assignments of the *D. sinicus* reference genome, previously determined via allele-aware chromosome-level analysis [27], were adopted as a reference framework. We selected *D. sinicus* as the synteny framework owing to its close placement to *Thyrsostachys* in published plastome phylogenies [81], together with its chromosome-level hexaploid assembly and strong collinearity with our genomes (Supplementary Fig. S4). In practice, protein-coding genes from the *T. oliveri* and *T. siamensis* assemblies were aligned to those of the *D. sinicus* reference using the jcvi synteny pipeline (v1.1.17) (RRID:SCR_018403) [82]. Syntenic gene blocks were identified, with the “–quota” parameter adjusted to reflect genome ploidy. By comparing the resulting syntenic blocks to the previously characterized subgenomes of *D. sinicus*, subgenome identities were inferred for the *T. oliveri* and *T. siamensis* assemblies. This methodology enabled the projection of the established *D. sinicus* subgenome structure onto the target genomes, thereby minimizing methodological redundancy.

### Expression bias between subgenomes

To investigate subgenome-specific gene expression in hexaploid *T. oliveri* and *T. siamensis*, we utilized 1:1:1 gene triads, identified using the methodology of [27]. Briefly, we defined a triad as expressed when the sum of the A, B, and C subgenome homoeologs had a transcript per million (TPM) >0.5 and standardized the relative expression of each homoeolog across the triad. The ternary diagrams were plotted using the R package ggtern [83]. To categorize homoeolog expression bias, we employed a method analogous to that used in wheat [84]. Ideal normalized expression biases for 6 distinct categories were defined. The Euclidean distance (calculated using the R function rdist) between the observed normalized expression of each triad and each ideal category was determined. Each triad was subsequently assigned to the category with the shortest Euclidean distance, representing its homoeolog expression bias, and this process was repeated for both leaf and root tissues.

## Results

### Genome assembly and evaluation

High-coverage stLFR libraries generated 408,544,881 reads (81.71 Gb) for *T. oliveri* and 401,766,290 reads (80.35 Gb) for *T. siamensis* (Supplementary Table S1). Genome size estimates for *T. oliveri* were 1.088 Gb by *k*-mer analysis and 1.226 Gb by flow cytometry, yielding an average of 1.157 Gb; for *T. siamensis*, estimates were 1.097 Gb (*k*-mer) and 1.247 Gb (flow cytometry), with a mean of 1.172 Gb (Supplementary Figs S5–S6). Using the mean genome sizes (1.157 Gb for *T. oliveri*; 1.172 Gb for *T. siamensis*), the stLFR yields (81.71 Gb and 80.35 Gb) correspond to ∼71× and ∼69× sequencing depth, respectively. *De novo* assembly of the stLFR reads produced 32,505 contigs (N50 = 2.45 Mb; 990 Mb) for *T. oliveri* and 77,021 contigs (N50 = 0.32 Mb; 1.14 Gb) for *T. siamensis* (Table 1). Heterozygosity estimates were 0.98% for *T. oliveri* and 2.67% for *T. siamensis* (Supplementary Fig. S6).

**Table 1:** Assembly statistics of *T. oliveri* and *T. siamensis*

	*T. oliveri*		*T. siamensis*	
	stLFR (prescaffold)	Hi-C (final)	stLFR (prescaffold)	RagTag (final)
N50 size (bases)	2,447,080	22,175,826	319,778	19,454,152
L50 number	86	18	390	23
N75 size (bases)	407,938	15,134,499	13,857	34,829
L75 number	313	31	7,916	629
N90 size (bases)	9,250	9,256	4,892	6,105
L90 number	5,845	4,941	29,083	15,413
Total (bases)	990,067,507	990,098,900	1,138,811,672	1,140,691,237
Number of scaffolds	32,505	31,592	77,021	57,746
Number of scaffolds ≥100 kb	566	51	964	119
Number of scaffolds ≥1 Mb	187	38	139	49
Number of scaffolds ≥10 Mb	10	35	5	36
Longest scaffold (bases)	15,491,168	40,971,832	14,091,541	40,067,183
GC content (%)	43.41	43.41	43.19	43.19
BUSCO evaluation (% completeness)	97.4	97.4	94.6	95.2

**Figure 1:**
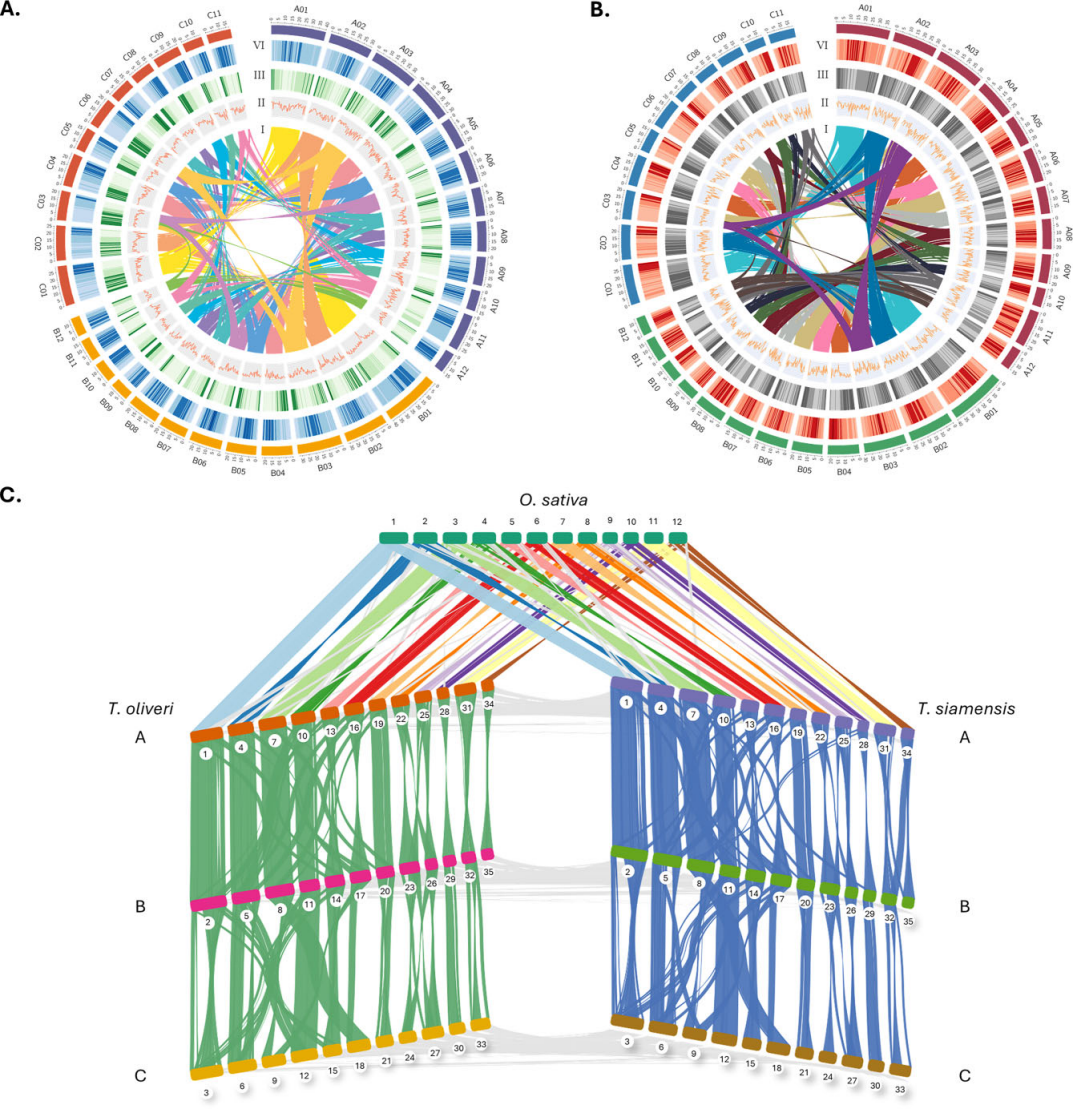
Genomic landscape and comparative collinearity of hexaploid *Thyrsostachys* and *Oryza sativa*. (A) Circos plot of the hexaploid *Thyrsostachys oliveri* assembly. The outermost ring shows chromosome ideograms for subgenome A (purple; 12 chromosomes), subgenome B (yellow; 12 chromosomes), and subgenome C (orange; 11 chromosomes). From the outermost data track inward: 1. Repeat content—proportion of bases covered by repetitive sequences in 500-kb windows. 2. Gene density—number of genes per 500-kb window. 3. GC content—percentage of G + C bases per 500-kb window. (B) Circos plot of the *T. siamensis* assembly, formatted identically to panel A. (C) Global collinearity between the 12 chromosomes of *O. sativa* and the A, B, and C subgenomes of *T. oliveri* (left) and *T. siamensis* (right).

For *T. oliveri*, 351 million Hi-C read pairs were used for scaffolding of the preliminary assembly to yield a scaffold N50 of 22.18 Mb and anchoring 81.4% (804 Mb) of the sequence into 35 pseudo-chromosomes, matching 2n = 6x = 70 (Fig. 1A and Supplementary Fig. S7). For *T. siamensis*, RagTag scaffolding against the *T. oliveri* reference produced a 19.45-Mb scaffold N50 with 69.2% (789 Mb) of the sequence anchored to the 35 *T. oliveri* pseudo-chromosomes (Table 1). Each assembly was partitioned into 3 homoeologous subgenomes (A, B, C). Extensive intra- and inter-subgenomic collinearity and large blocks aligning to the 12 *O. sativa* chromosomes are shown in Fig. 1A–C.

BUSCO analysis (Embryophyta odb10; 1,614 genes) recovered 97.4% complete genes in *T. oliveri* and 95.2% in *T. siamensis* (Table 1). Read-mapping rates further supported assembly accuracy: 98.83%/98.10% of stLFR reads and 90.22%/87.62% of RNA-seq reads mapped back to the *T. oliveri* and *T. siamensis* genomes, respectively. In proteome mode (embryophyta_odb10), BUSCO completeness was 86.0% for *T. oliveri* and 83.0% for *T. siamensis*, directly assessing annotation completeness. Together with LAI scores of 7.75 and 7.10 and Merqury QV values of 55.85 and 52.5 for *T. oliveri* and *T. siamensis*, respectively (Supplementary Table S12), these metrics indicate chromosome-scale assemblies with high per-base accuracy and broad gene space recovery suitable for comparative and functional genomics. The LAI values (<10) also indicate moderate repeat continuity.

### Genome annotation

To annotate the *Thyrsostachys* genomes, we employed an integrated pipeline combining *ab initio* prediction, RNA-seq supported evidence, and protein homology evidence. This analysis revealed distinct differences in the genomic composition of the 2 bamboo species. *T. oliveri* contained 51,191 predicted gene models, of which 48,070 were protein-coding; *T. siamensis* exhibited 67,483 predicted models with 59,683 protein-coding genes. Average genomic GC content was similar between species (43.41% in *T. oliveri*, 43.19% in T*. siamensis*), with exons enriched in GC (53.4% and 54.7%, respectively) and introns lower (39.8% and 39.0%) (Table 2). Relative to 11 recently published bamboo genomes [27], *Thyrsostachys* shows more compact gene models, with shorter genes and introns overall, while median exon size is broadly conserved; exon number is ∼5 in *T. oliveri* and lower in *T. siamensis* (4.27). Full cross-species feature values are provided in Supplementary Table S2.

**Table 2:** Annotation statistics for *T. oliveri* and *T. siamensis*

	*T. oliveri*	*T. siamensis*
Number of predicted gene models	51,191	67,483
Total gene length (Mb)	146.26	179.99
Average gene size (nt)	2,857	2,667
Average number of exons/gene	5.04	4.27
Total exon length (Mb)	55.98	71.55
Average exon length (nt)	216.8	248.2
GC content of exons (%)	53.4	54.69
Average number of Introns/gene	4.04	3.27
Total intron length (Mb)	90.33	108.51
Average intron length (nt)	436.4	491.5
GC content of introns (%)	39.81	38.99

Of these protein-coding genes, 93.9% in *T. oliveri* and 88.4% in *T. siamensis* had best hits in the NCBI NR database. GO terms were assigned to 72.4% of *T. oliveri* genes and 77.8% of *T. siamensis* genes, and KEGG pathways to 32.2% and 28.1%, respectively (Supplementary Table S3). In the GO biological process category, *T. oliveri* was enriched for regulation of DNA-templated transcription, transmembrane transport, and protein ubiquitination, whereas *T. siamensis* showed regulation of DNA-templated transcription, regulation of transcription by RNA polymerase II, and chromatin remodeling. Membrane, nucleus, and cytoplasm dominated the cellular component category in both species, while ATP binding, metal/zinc-ion binding, and DNA binding dominated the molecular function category (Supplementary Figs S8–S9).

For the ncRNAs, we also identified 78,048 microRNAs, 1,009 tRNAs, 320 rRNAs, and 13,420 small nuclear RNAs in the *T. oliveri* genome (Supplementary Table S4). Similarly, the *T. siamensis* genome contained 36,297 microRNAs, 1,049 tRNAs, 336 rRNAs, and 11,678 small nuclear RNAs (Supplementary Table S5).

### Identification of repetitive elements

Comparative analysis of repetitive elements in the 2 *Thyrsostachys* genomes reveals both shared architecture and lineage-specific variation. We grouped repeats into known classes (e.g., LTR, LINE, DNA transposons) and an unclassified category (Other) for sequences lacking clear annotation. Repeats constitute 50.89% of the *T. oliveri* assembly (Table 3) and 48.78% of the *T. siamensis* assembly (Table 4). In *T. oliveri*, unclassified repeats are most abundant (64.55% of all repeats), followed by retrotransposons (24.94%). Within retrotransposons, LTR elements of the Copia and Gypsy superfamilies contribute 13.09% and 9.86% of the genome, respectively. *T. siamensis* exhibits a similar profile: unclassified repeats dominate (67.88%), with Copia and Gypsy elements contributing 13.00% and 8.20% of the genome, respectively. The overall repeat content is approximately 2 percentage points lower in *T. siamensis*, mainly owing to its smaller Gypsy fraction.

**Table 3:** Repeat elements in the *T. oliveri* genome assembly

Types of repeats	Bases (Mb)	% of the assembly	% of total repeats
**DNA transposons**	47.68	4.81	9.46
**Retrotransposons**			
LINE	8.47	0.85	1.68
SINE	0.09	0.00	0.00
LTR: *Copia*	65.94	6.66	13.09
LTR: *Gypsy*	49.68	5.02	9.86
LTR: Others	1.56	0.16	0.31
**Simple sequence repeats**	5.31	0.54	1.05
**Others**	325.08	32.85	64.55
**Total**	503.81	50.89	

**Table 4:** Repeat elements in the *T. siamesis* genome assembly

Types of repeats	Bases (Mb)	% of the assembly	% of total repeats
**DNA transposons**	45.77	4.01	8.22
**Retrotransposons**			
LINE	9.09	0.80	1.63
SINE	0.00	0.00	0.00
LTR: *Copia*	72.08	6.32	13.00
LTR: *Gypsy*	45.62	4.00	8.20
LTR: Others	0.31	0.03	0.05
**Simple sequence repeats**	5.70	0.50	1.02
**Others**	377.90	33.12	67.88
**Total**	556.47	48.78	

### Phylogenetic and comparative genomics analyses

**Figure 2:**
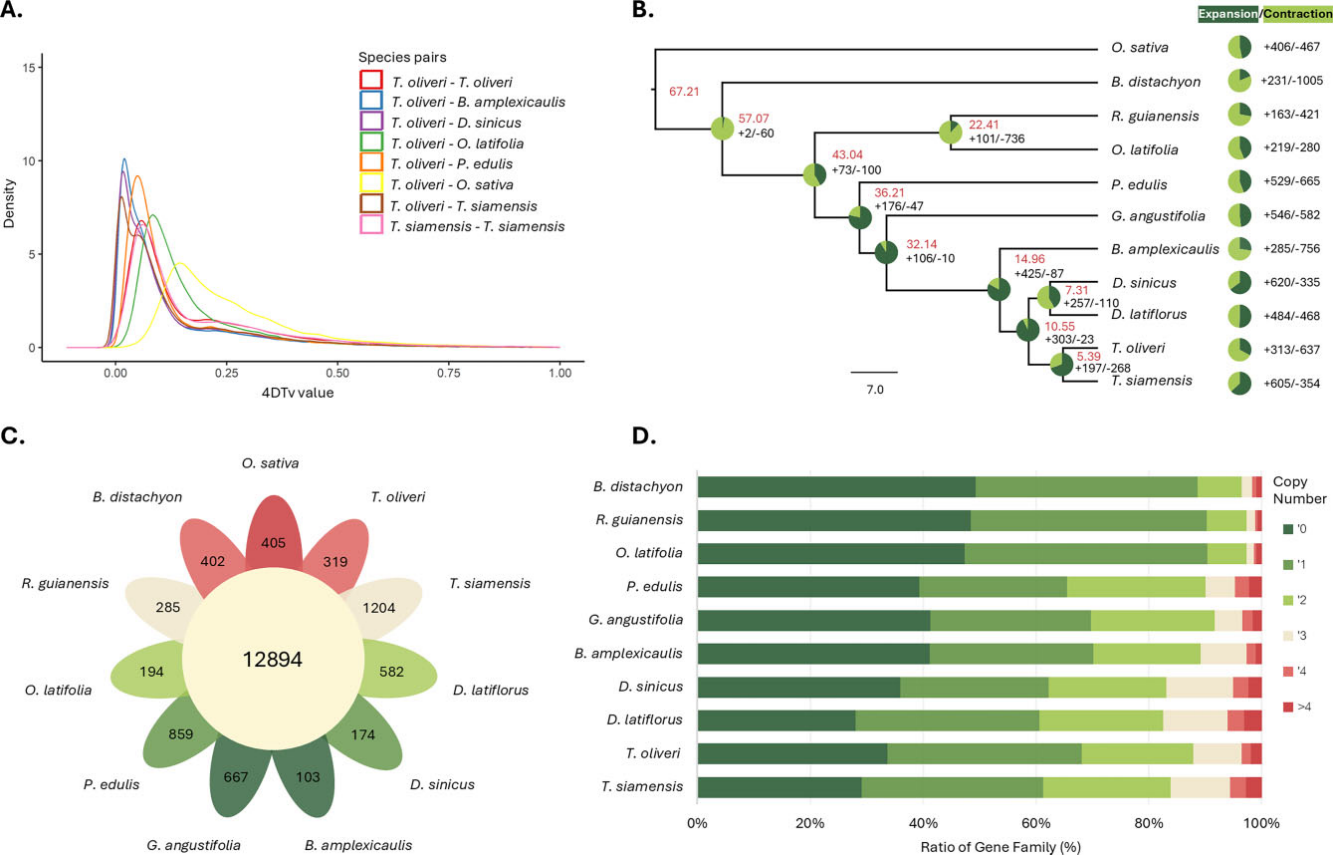
Evolutionary analysis of the hexaploid *Thyrsostachys* genome. (A) Density plot showing pairwise 4-fold synonymous (degenerative) third-codon transversion (4DTv) values between the indicated pairs of genomes. (B) Phylogenetic tree of 11 plant species, with the predicted divergence time shown as each branch. Adjacent pie charts show numbers of gene family expansion (shown in light green) and contraction (shown in dark green) among 11 species. The size and color of the circles represent the proportion of genes. (C) Flower‐petal plot showing orthologous gene‐family sharing among the 11 species (center circle) and species-specific gene families (individual petals). (D) Stacked bar chart of gene family copy-number distributions across the 11 species. Bars are partitioned into categories (0, 1, 2, 3, 4, >4 copies); colors correspond to each copy-number class (legend at right).

To estimate the relative timing of divergence and whole-genome duplication (WGD) events in the *Thyrsostachys* lineage, we quantified 4-fold degenerate transversions (4DTvs) for orthologous and paralogous gene pairs and synonymous substitution rates (Ks) for syntenic anchor pairs. The interspecific distances are concordant across metrics, with the closest affinity between *T. oliveri* and *T. siamensis* (4DTv = 0.0164; Ks = 0.0131), followed by *D. sinicus* (0.0178; 0.0375), *B. amplexicaulis* (0.0180; 0.0416), *P. edulis* (0.0516; 0.135), and *O. latifolia* (0.0810; 0.2184) (Fig. 2A and Supplementary Fig. S10). Within genomes, 4DTv distributions of paralogous pairs (*n* = 28,430 in *T. oliveri; n* = 28,061 in *T. siamensis*) are bimodal, with peaks at 0.056 and 0.208 in *T. oliveri* and 0.059 and 0.231 in *T. siamensis*; the corresponding Ks distributions show peaks at 0.150 and 0.516 in *T. oliveri* and 0.152 and 0.533 in *T. siamensis*. Concordant 4DTv and Ks modes support a relatively recent WGD shared by the 2 *Thyrsostachys* species.

Next, we reconstructed a maximum likelihood phylogeny from 605 single-copy orthologs sampled from *Thyrsostachys* and 8 reference grasses (*B. amplexicaulis, P. edulis, D. latiflorus, D. sinicus, G. angustifolia, R. guianensis, B. distachyon*, and *O. sativa*) (Fig. 2B). The 2 *Thyrsostachys* species form a clade sister to the *Dendrocalamus* pair, with a crown age of 5.39 Mya and a stem divergence from *Dendrocalamus* at 10.55 Ma.

To test whether the *Thyrsostachys* A, B, and C subgenomes correspond to the core A, B, and C subgenomes in other hexaploid bamboos, we inferred orthogroups and gene trees with OrthoFinder using proteomes from *Thyrsostachys* (this study) and 3 hexaploids (*M. baccifera, B. amplexicaulis, D. sinicus*). For single-copy orthogroups with representation across species, we labeled each sequence by its subgenome and assessed subgenome-specific clustering. Across gene trees, A copies from different species cluster together, and likewise for B and C, indicating that the *Thyrsostachys* subgenomes are orthologous to the core A, B, and C subgenomes described for other hexaploids (Supplementary Fig. S11).

Orthogroup clustering placed 436,476 of 469,186 proteins (93%) into 37,153 families. Within this 11-species framework, a core set of 12,894 families is shared by all 11 species (Fig. 2C), while species-unique families number 319 in *T. oliveri*, enriched for root development and plasmodesmata genes, and 1,204 in *T. siamensis*, highlighting histone-binding and proteasome pathways (Supplementary Tables S6–S7).

Finally, gene copy profiling revealed that hexaploid *D. latiflorus* has undergone the greatest expansion of gene families, followed by *T. siamensis*, with *T. oliveri* showing fewer duplications (Fig. 2D). In *T. oliveri*, 313 families expanded and 637 contracted; in *T. siamensis*, 605 expanded and 354 contracted. GO enrichment of *T. oliveri* expansions highlights protein kinase activity, defense response, and monooxygenase activity (Supplementary Figs. S12–S13), whereas *T. siamensis* expansions are enriched for ADP binding, carbohydrate binding, and DNA integration functions (Supplementary Figs. S14–S15). At the *Thyrsostachys* crown node, CAFE identifies significant shifts in gene family size, with 197 expansions and 268 contractions. Contracted families are enriched for cell-surface receptor protein Ser/Thr-kinase signaling and broader defense/interaction processes (e.g., defense responses to bacteria and oomycetes), together with oxidoreductase functions and polyamine biosynthesis (adenosylmethionine decarboxylase, spermidine/spermine biosynthetic process). A plasma membrane cellular component signal among contractions is compatible with reductions in some membrane-localized receptor-like kinase repertoires (Supplementary Table S8). In contrast, expanded families at this ancestral node are enriched for stress/interaction responses (notably defense against other organisms), osmolyte transport (L-proline/proline transport), proteostasis and organelle-processing functions (proteasome subcomplex, mitochondrial inner-membrane peptidase), and ADP/phosphate binding and transport. Collectively, these patterns indicate a lineage-level remodeling of stress and signaling-related gene content at the *Thyrsostachys* crown node (Supplementary Table S8).

### Homoeolog expression patterns

**Figure 3:**
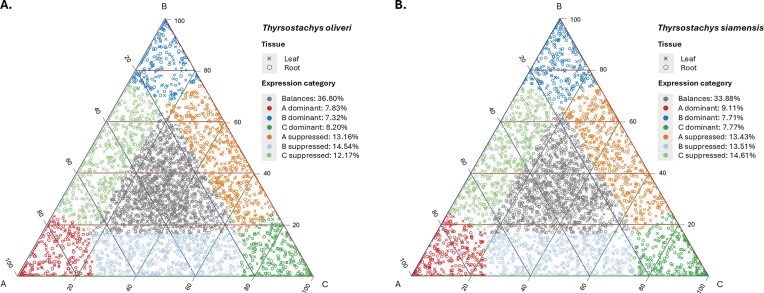
Homoeolog expression patterns in the hexaploid *Thyrsostachys* genome. Ternary plots of relative expression contributions of the A, B, and C subgenome homoeologs in (A) *T. oliveri* and (B) *T. siamensis*. Each point represents 1 expressed gene triad (sum TPM > 0.5) in either leaf (×) or root (°) tissue; its position reflects the proportion of total expression contributed by each homoeolog. • Vertices (red, blue, or green) indicate single‐homoeolog dominance (A-, B-, or C-dominant). • Edges (orange, light blue, or light green) indicate single‐homoeolog suppression (A-, B-, or C-suppressed). • Center (gray) indicates balanced expression among all 3 homoeologs. Percentages in the legend denote the fraction of expressed triads in each category.

From RNA-seq of leaf and root (3 biological replicates each), we identified 2,197 homoeologous triads in *T. oliveri* and 2,054 triads in *T. siamensis*. Triads in each tissue with a combined expression of A, B, and C homoeolog expression greater than 0.5 TPM were designated “expressed” and classified into 6 relative-expression categories (Fig. 3 and Supplementary Table S9). Balanced expression was observed in 36.8% of expressed *T. oliveri* triads and 33.8% of expressed *T. siamensis* triads, whereas single-homoeolog dominance was the least common (23.4% and 24.6%, respectively). Single-homoeolog suppression affected 39.9% of expressed *T. oliveri* triads and 41.8% of expressed *T. siamensis* triads. Within the dominance categories, B-homoeolog dominance was rare (7.3% in *T. oliveri*; 7.7% in *T. siamensis*), while A- and C-homoeolog dominance showed slightly higher frequencies. Among the suppression categories, B-homoeolog suppression was most frequent in *T. oliveri* (14.5%), whereas C-homoeolog suppression led in *T. siamensis* (14.6%) (Supplementary Table S9).

### Functional enrichment of homoeolog bias categories

We performed GO enrichment across 6 bias categories in leaf and root tissues of *T. oliveri* and *T. siamensis* (Supplementary Table S10); 5 modules recurred: (i) photosynthesis/growth: C-dominant leaves are enriched for chlorophyll and gibberellin/terpenoid biosynthesis, indicating a disproportionately large contribution from subgenome C to photosynthesis and growth in leaves. (ii) Cell wall/carbohydrate flux: In *T. oliveri*, B-dominant sets emphasize GDP mannose supply and cell wall organization; in *T. siamensis*, they highlight broad transmembrane transport and symporter activity, consistent with roles in carbon allocation. (iii) Hormone and redox signaling: Auxin/brassinosteroid and oxidative stress detoxification terms concentrate in A- and B-suppressed sets, supporting hormone–reactive oxygen species crosstalk under stress. (iv) Membrane trafficking/organelles: A- and B-suppressed sets are enriched for ESCRT/HOPS, vacuole organization, and nuclear pore/transport, indicating reduced A/B contribution to these pathways and partitioning across subgenomes rather than pathway absence. (v) Organelle metabolism: Respiration and chloroplast compartment terms co-occur across subgenomes, indicating coordinated mitochondrial–chloroplast support. Together, these 5 modules are consistent with subgenome C preferentially supporting leaf photosynthesis and growth, subgenome B being associated with cell wall biogenesis and carbon allocation, and subgenome A contributing to terpenoid/sterol metabolism and translational/quality control. Endomembrane remodeling signals are prominent. To demonstrate subgenome-specific expression biases that differ between species, we highlight a mannose-6-phosphate isomerase (PMI; GO:0004476) triad that is B-dominant in *T. oliveri* leaves but not B-dominant in *T. siamensis* leaves, illustrating species-specific subgenome bias. Full term lists and statistics are provided in Supplementary Table S10.

### Subgenome composition and gene retention in *Thyrsostachys*

To place the expression results in structural context, we quantified gene retention across the A, B, and C subgenomes by classifying gene sets into triads (1:1:1 across A/B/C), duplets (present in exactly 2 subgenomes), singletons (present in 1), and other multicopy configurations, including copy-number variants (CNVs; any class in which a present subgenome contributes >1 copy) (Supplementary Table S11).

In *T. oliveri*, subgenomes A, B, and C contain 15,803, 14,691, and 13,627 genes, with 2,197 genes per subgenome in 1:1:1 triads (13.90%, 14.95%, 16.12%). Among nontriad classes, duplets account for 33.80%, 32.82%, and 31.84% and singletons for 3.46%, 2.55%, and 1.74%; the remaining genes fall into other multicopy configurations (including CNVs) at 48.85%, 49.68%, and 50.30% for A, B, and C, respectively.

In *T. siamensis*, subgenomes A, B, and C contain 17,670, 16,171, and 14,838 genes; 2,054 genes per subgenome are in 1:1:1 triads (11.62%, 12.70%, 13.84%). Duplets comprise 31.77%, 29.97%, and 29.72%; singletons 4.24%, 3.36%, and 2.47%; and other multicopy configurations (including CNVs) 52.37%, 53.97%, and 53.97% for A, B, and C, respectively.

Across both species, subgenome C consistently shows the highest triad retention and the lowest absence-bearing fraction (duplet + singleton; 33.58% in *T. oliveri*, 32.19% in *T. siamensis*), whereas subgenome A shows the greatest loss (37.26% and 36.01%). These complementary patterns of loss and duplication indicate asymmetric fractionation among subgenomes following polyploidization (Supplementary Table S11).

## Discussion

Bamboo is one of the world’s most important nontimber forest resources and a crucial component of forest ecosystems. Despite its significance, only a limited number of bamboo genomes have been sequenced to date, resulting in limited knowledge of bamboo biological mechanisms and hindering progress in understanding genome evolution and the potential for molecular breeding. In this study, we sequenced and assembled chromosome-level genomes of 2 bamboo species, *T. oliveri* and *T. siamensis*, which resulted in the first reference genome for the *Thyrsostachys* genus. The assembled genome sizes of *T. oliveri* and *T. siamensis* were 990.1 Mb (N50 = 22.18 Mb) and 1.14 Gb (N50 = 19.45 Mb), respectively. The assemblies were anchored into 35 pseudochromosomes, and subgenome partitioning identified 3 distinct subgenomes, supporting a hexaploid structure (2n = 6x = 70). Compared with previously reported hexaploid bamboo genome assemblies, the genome sizes of *T. oliveri* and *T. siamensis* are smaller than those of *D. latiflorus* (1,368 Mb, 1C) [22] and *D. brandisii* (1,378 Mb, 1C) [21] but larger than that of *B. amplexicaulis* (848 Mb) [9]. Based on the BUSCO assessment, the completeness of the gene space in the *T. oliveri* and *T. siamensis* genomes was estimated at 97.4% and 95.2%, respectively, indicating that the current assemblies cover most of their genomes. The assembly size of *T. oliveri* is slightly smaller than the estimated genome size based on both *k*-mer analysis and flow cytometry (1.157 Gb). This slight difference likely reflects unassembled repetitive portions of the genome. In contrast, the assembly size of *T. siamensis* is close to the estimates from both methods (1.172 Gb). Compared with PacBio HiFi references, our stLFR + Hi-C assemblies achieve chromosome-scale, scaffold N50s and comparable BUSCO completeness (Supplementary Table S12) [22, 26, 85], while exhibiting lower repeat continuity (LAI) and base-level QV, as expected for short-read barcoded libraries. These trade-offs primarily affect long repetitive regions and do not preclude the gene space and comparative analyses reported here. While Merqury QV indicates very high per-base accuracy, our LAI scores (<10) point to moderate repeat continuity, consistent with assemblies derived from 150-bp stLFR short reads. Long LTR retrotransposons and other complex or highly similar repeats are likely fragmented or collapsed, and some homoeologous regions in this hexaploid context may remain underresolved. Future long-read (e.g., HiFi/ONT) and T2T scaffolding would further improve structural completeness and repeat resolution.

The genome annotations of *T. oliveri* and *T. siamensis* contained 51,191 and 67,483 predicted gene models, respectively. Compared with other bamboo genomes, the number of annotated genes in the haploid assembly of *T. oliveri* is similar to that reported for *P. edulis* (51,074) [13] and higher than that of *B. amplexicaulis* (47,056) [9]. In contrast, the 67,483 genes in the haploid *T. siamensis* assembly correspond to roughly half the total counts observed in diploid‐level assemblies: 135,231 genes for *D. latiflorus* [22] and 126,817 genes for *D. brandisii* [21], both of which were assembled at 2n = 70. The proportions of repetitive sequences identified in our genome assemblies were 50.89% for *T. oliveri* and 48.78% for *T. siamensis*. The composition of repeat types was highly similar between the 2 species, which is unsurprising given their close evolutionary relationship. The content of repetitive elements in our genome assemblies is slightly lower than that reported for moso bamboo [13, 23], *D. latiflorus* [22], and *D. brandisii* [21]. LTR elements are the predominant retrotransposon in our bamboo assemblies. Consistent with previous studies, LTR retroelements are also the most common elements in bamboo genomes [13, 21, 22]. Phylogenetic analysis using single-copy orthologs placed *T. oliveri* and *T. siamensis* as sister taxa, diverging approximately 5.39 Mya. Consistent signals from 4DTv and Ks analyses (using syntenic anchors) indicate that a recent, shared WGD event occurred in their common ancestor before this speciation.

Gene family analysis revealed distinct genomic features contributing to the phenotypic specificity and adaptive divergence of *T. oliveri* and *T. siamensis*. While 12,894 gene families were shared across 11 species, *T. oliveri* and *T. siamensis* possessed 319 and 1,204 species-specific gene families, respectively, with functions potentially related to root development and carbon metabolism in *T. oliveri* and protein degradation in *T. siamensis*. In our analysis, *T. oliveri* was found to have fewer expanded gene families and more contracted gene families compared to *T. siamensis*. In contrast, *T. siamensis* exhibited a greater number of expanded gene families and fewer contractions. Given that *T. siamensis* also had a higher overall gene count, it is likely that the expanded families in this species contain more gained genes than were lost through contraction, potentially contributing to its distinct genomic and phenotypic features such as rapid growth and high shoot productivity.

By examining the expanded gene families in *T. oliveri*, we found enrichment in genes associated with protein kinase activity and monooxygenase activity that may be linked to signaling pathways and metabolic regulation under more specialized ecological conditions. In *T. siamensis*, expanded gene families were enriched in functions related to ADP binding and protein dimerization activity that potentially support its rapid growth, high shoot productivity, and broader environmental adaptability relative to *T. oliveri* and other woody bamboos. When compared with other woody bamboos, *P. edulis* shows expansions in gene families associated with lignin biosynthesis and has the highest copy numbers in the peroxidase gene family [13]. In contrast, *D. latiflorus* exhibits expansions in genes involved in telomere maintenance and DNA repair, which likely play important roles in supporting its long‐lived vegetative growth [22]. These species-specific expansions and contractions of gene families therefore provide insights into the phenotypic characteristics, adaptive evolution, and unique evolutionary pressures that have shaped the genomes of *Thyrsostachys* species compared with other bamboo lineages. At the genus level, our gene family analysis revealed that the contraction of receptor-like kinase, pathogen response, and polyamine biosynthesis families suggests streamlining of certain signaling and defense pathways, whereas expansion of stress response, osmolyte transport, and proteostasis families indicates adaptive innovations that enhance cellular homeostasis.

Allopolyploidy is a defining feature of many bamboo species. Previous studies have shown that duplicated gene pairs may display homoeolog expression bias in several allopolyploid species [86–90], in which bias refers to the preferential expression of one homoeolog relative to the other [91]. In our hexaploid *Thyrsostachys* species, single-homoeolog suppression was the most frequent expression category, affecting 39.9% of expressed triads in *T. oliveri* and 41.8% in *T. siamensis*, a pattern also observed in other woody bamboos [27]. Balanced expression accounted for 36.8% of triads in *T. oliveri* and 33.8% in *T. siamensis*, consistent with coordinated subgenome regulation observed in hexaploid bamboo species [27]. Single‐homoeolog dominance was relatively uncommon (<25%). Among the dominant triads, the B-subgenome was the least frequently dominant, while A- and C-subgenomes showed comparable but slightly higher levels of dominance. These findings are consistent with previous reports in *M. baccifera, B. amplexicaulis*, and *D. sinicus* bamboo species [27]. Suppression biases differed between species: B‐subgenome suppression predominated in *T. oliveri*, whereas C‐subgenome suppression was more frequent in *T. siamensis*, suggesting lineage‐specific regulatory divergence. Together, these findings indicate that, while balanced expression remains substantial, single‐homoeolog suppression is a major driver of subgenome‐specific expression and may underlie functional differentiation in these two *Thyrsostachys* species.

## Supplementary Material

giaf142_Supplemental_Files

giaf142_Authors_Response_To_Reviewer_Comments_Original_Submission

giaf142_GIGA-D-25-00232_Original_Submission

giaf142_GIGA-D-25-00232_Revision_1

giaf142_Reviewer_1_Report_Original_Submission Hansheng Zhao -- 7/31/2025

giaf142_Reviewer_1_Report_Revision_1 Hansheng Zhao -- 10/20/2025

giaf142_Reviewer_2_Report_Original_Submission Mingbing Zhou -- 8/2/2025

giaf142_Reviewer_2_Report_Revision_1 Mingbing Zhou -- 10/10/2025

giaf142_Reviewer_3_Report_Original_Submission Lianfeng Gu -- 8/5/2025

## Data Availability

Both *T. oliveri* and *T. siamensis* genome assemblies have been deposited at NCBI under accession numbers JAWCWW000000000 and JBEFOJ000000000, respectively. The raw stLFR reads for *T. oliveri* and *T. siamensis* are available in the NCBI SRA database under accession numbers SRR32915794 and SRR32915889, respectively. The transcriptome data for *T. oliveri* were submitted under SRR33015685 and SRR33015684. The transcriptome data for *T. siamensis* were submitted under SRR33015804 and SRR33015803. All additional supporting data are available in the *GigaScience* repository, GigaDB [92].

## Additional Files

**Supplementary Table S1**. Summary of sequencing data from stLFR and Hi-C platforms.

**Supplementary Table S2**. Comparative gene features across *Thyrsostachys* and 11 bamboo genomes.

**Supplementary Table S3**. Functional annotations of *T. oliveri* and *T. siamensis* protein-coding genes.

**Supplementary Table S4**. Noncoding RNA in the *T. oliveri* genome.

**Supplementary Table S5**. Noncoding RNA in the *T. siamensis* genome.

**Supplementary Table S6**. GO term enrichment analyses for gene families specific to *T. oliveri*.

**Supplementary Table S7**. GO term enrichment analyses for gene families specific to *T. siamensis*.

**Supplementary Table S8**. GO term enrichment analyses for gene families specific to *Thyrsostachys* crown node.

**Supplementary Table S9**. Percentage of syntenic triads assigned to the 6 homoeolog expression bias categories.

**Supplementary Table S10**. GO enrichment of homoeolog expression bias categories in leaf and root of 2 *Thyrsostachys* bamboos.

**Supplementary Table S11**. Subgenome composition and distribution of 1:1:1 triads, duplets, and singletons in *Thyrsostachys*.

**Supplementary Table S12**. Assembly technology and key metrics across *Thyrsostachys* and PacBio long-read bamboo references.

**Supplementary Fig. S1**. *Thyrsostachys oliveri* morphology.

**Supplementary Fig. S2**. *Thyrsostachys siamensis* morphology.

**Supplementary Fig. S3**. Example of manual Hi-C curation performed by Biomarker Technologies (BMK).

**Supplementary Fig. S4**. Collinearity between *Thyrsostachys* genomes and *D. sinicus*.

**Supplementary Fig. S5**. Flow-cytometric estimation of nuclear DNA content in *Thyrsostachys* species.

**Supplementary Fig. S6**. Genome survey *k*-mer distributions for hexaploidy *Thyrsostachys*.

**Supplementary Fig. S7**. Genome-wide Hi-C contact map of hexaploid *Thyrsostachys oliveri*.

**Supplementary Fig. S8**. GO annotation statistics for *T. oliveri*.

**Supplementary Fig. S9**. GO annotation statistics for *T. siamensis*.

**Supplementary Fig. S10**. Density plot showing pairwise synonymous substitution rate (Ks) values between the indicated pairs of genomes.

**Supplementary Fig. S11**. Subgenome-specific clustering of single-copy orthologs across hexaploid bamboos.

**Supplementary Fig. S12**. GO term enrichment analyses for expanded gene families in *T. oliveri*.

**Supplementary Fig. S13**. GO term enrichment analyses for contracted gene families in *T. oliveri*.

**Supplementary Fig. S14**. GO term enrichment analyses for expanded gene families in *T. siamensis*.

**Supplementary Fig. S15**. GO term enrichment analyses for contracted gene families in *T. siamensis*.

## Abbreviations

4DTv: 4-fold degenerate transversion; BLASTP: Basic Local Alignment Search Tool for Protein; bp: basepair; BUSCO: Benchmarking Universal Single-Copy Orthologs; CNVs: copy-number variants; ESS: effective sample size; EVM: EvidenceModeler; Gb: gigabase; GO: Gene Ontology; Hi-C: chromosome conformation capture; HMW: high molecular weight; kb: kilobase; KEGG: Kyoto Encyclopedia of Genes and Genomes; LAI: long terminal repeat assembly index; LTR: long terminal repeat; Mb: megabase; Mya: million years ago; ncRNA: noncoding RNA; NR: nonredundant; QV: quality value; RNA-seq: RNA sequencing; stLFR: single-tube long-fragment read; non-TE: transposable element; TPM: transcript per million; WGD: whole-genome duplication.
