# Supplementary figures and images for: Chromosome-level assemblies of two hexaploid bamboos, Thyrsostachys oliveri and Thyrsostachys siamensis, provide a foundation for functional and comparative genomics studies
Source: Gigascience. 2025 Nov 17;14:giaf142. doi: 10.1093/gigascience/giaf142 (PMC12699669; doi:10.1093/gigascience/giaf142)

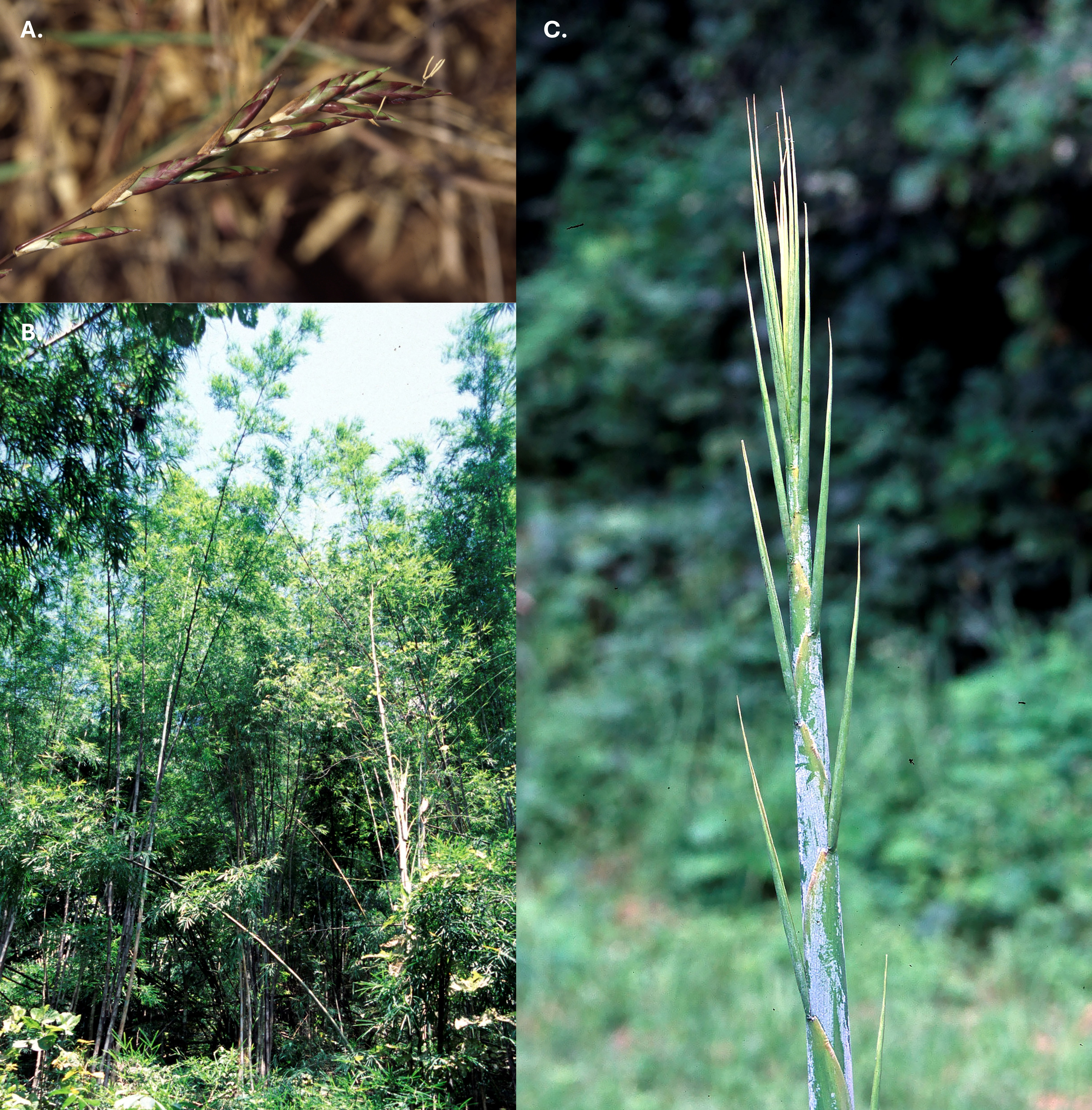

Supplement: giaf142_Supplemental_Files [file giaf142_supplemental_files.zip › Supplementary Fig. S1.PNG]

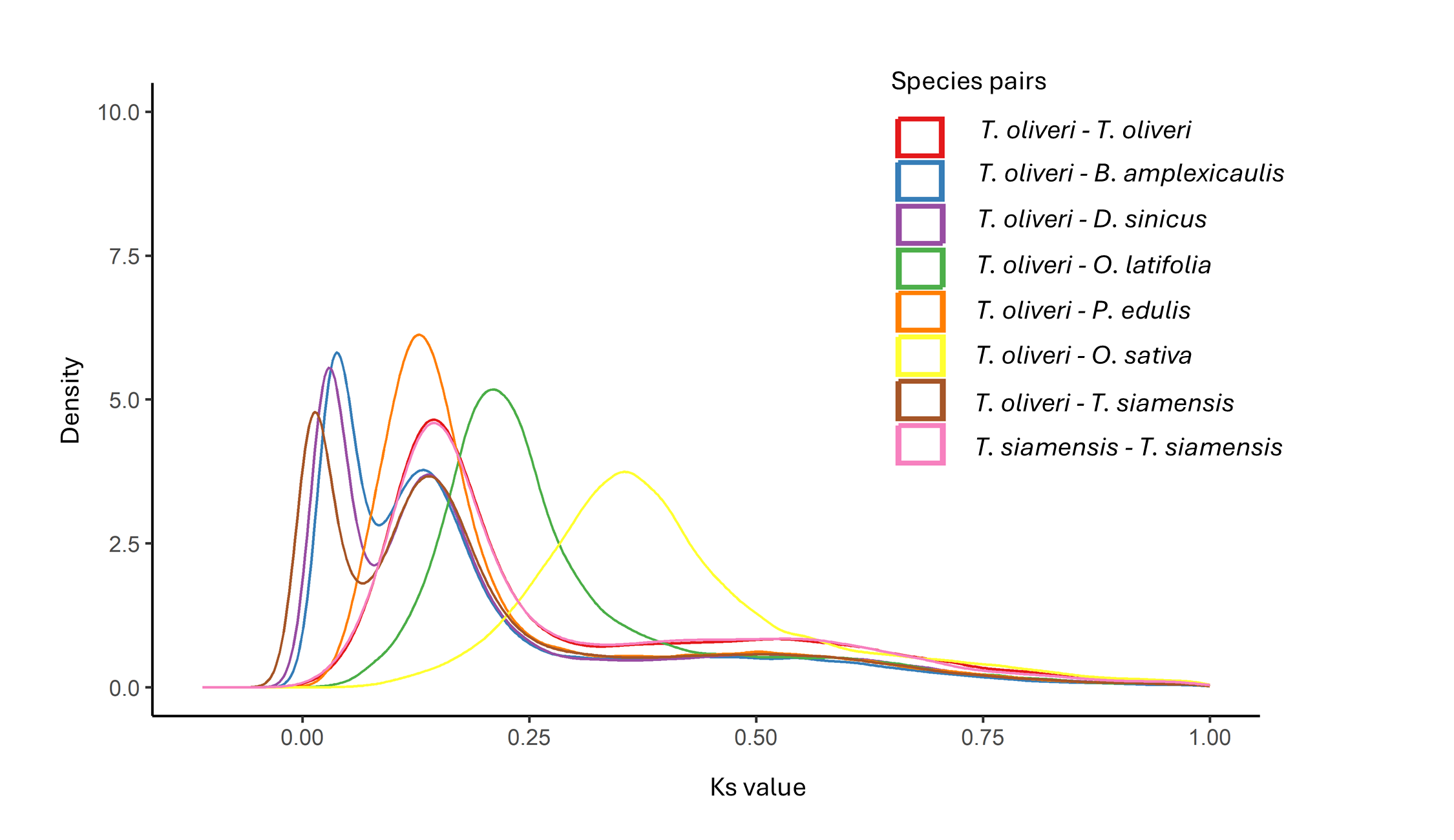

Supplement: giaf142_Supplemental_Files [file giaf142_supplemental_files.zip › Supplementary Fig. S10.png]

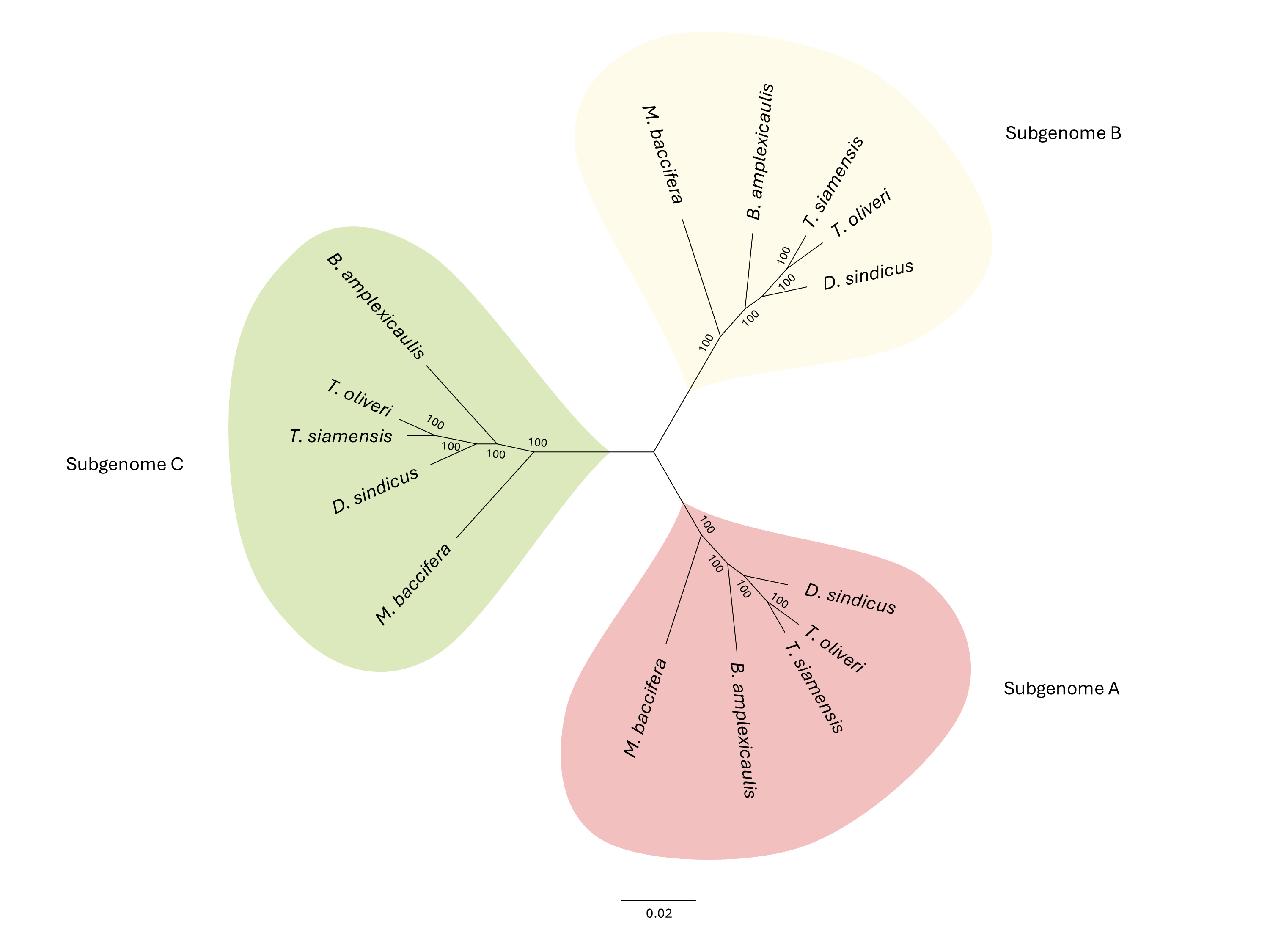

Supplement: giaf142_Supplemental_Files [file giaf142_supplemental_files.zip › Supplementary Fig. S11.tif]

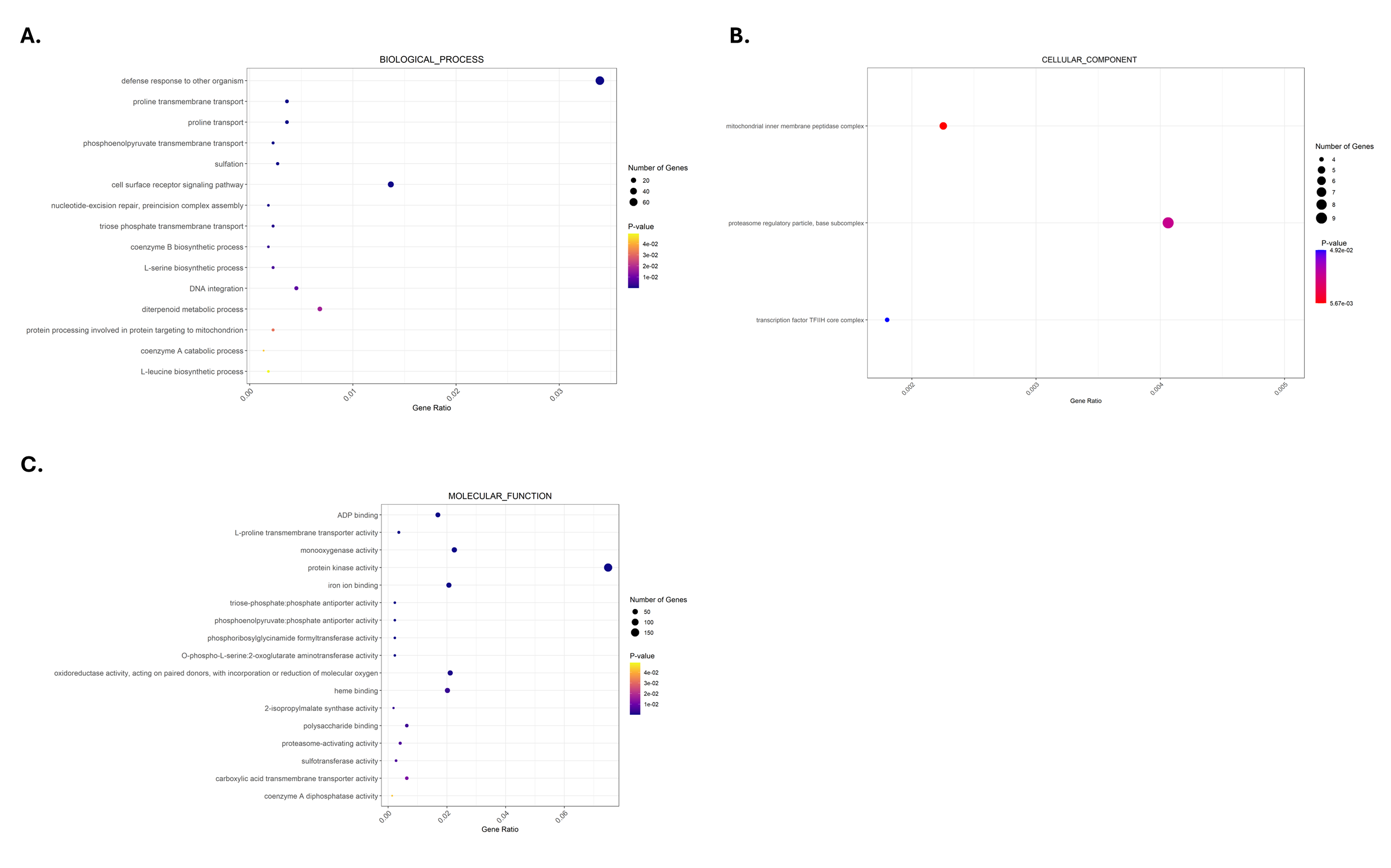

Supplement: giaf142_Supplemental_Files [file giaf142_supplemental_files.zip › Supplementary Fig. S12.PNG]

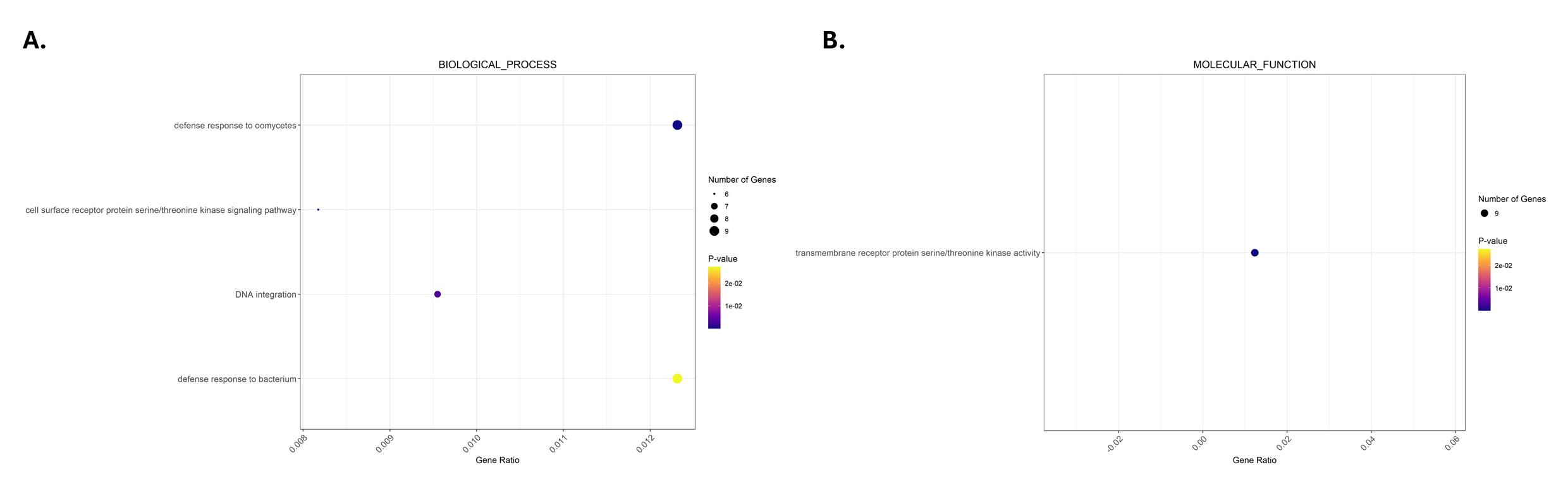

Supplement: giaf142_Supplemental_Files [file giaf142_supplemental_files.zip › Supplementary Fig. S13.PNG]

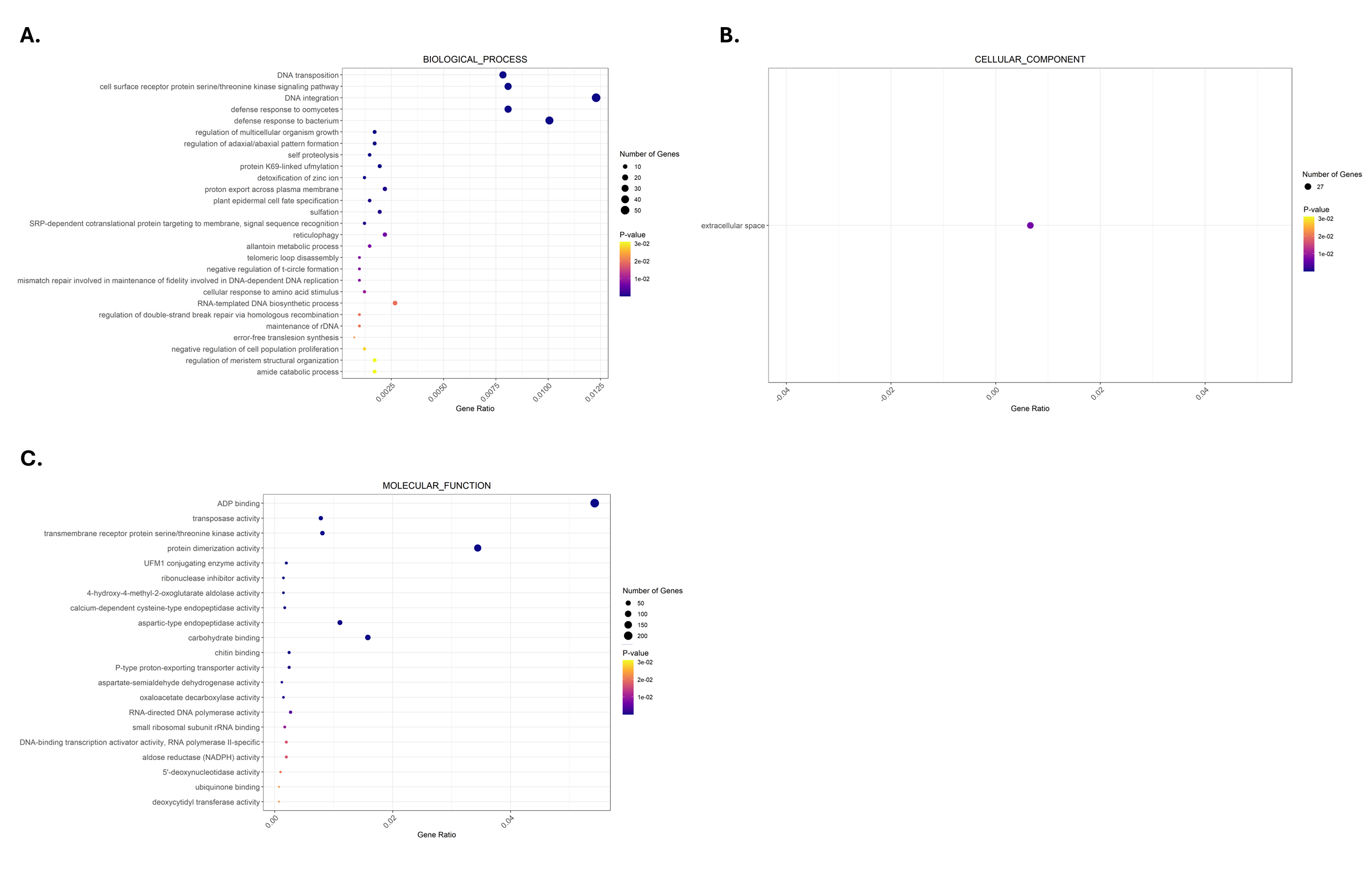

Supplement: giaf142_Supplemental_Files [file giaf142_supplemental_files.zip › Supplementary Fig. S14.PNG]

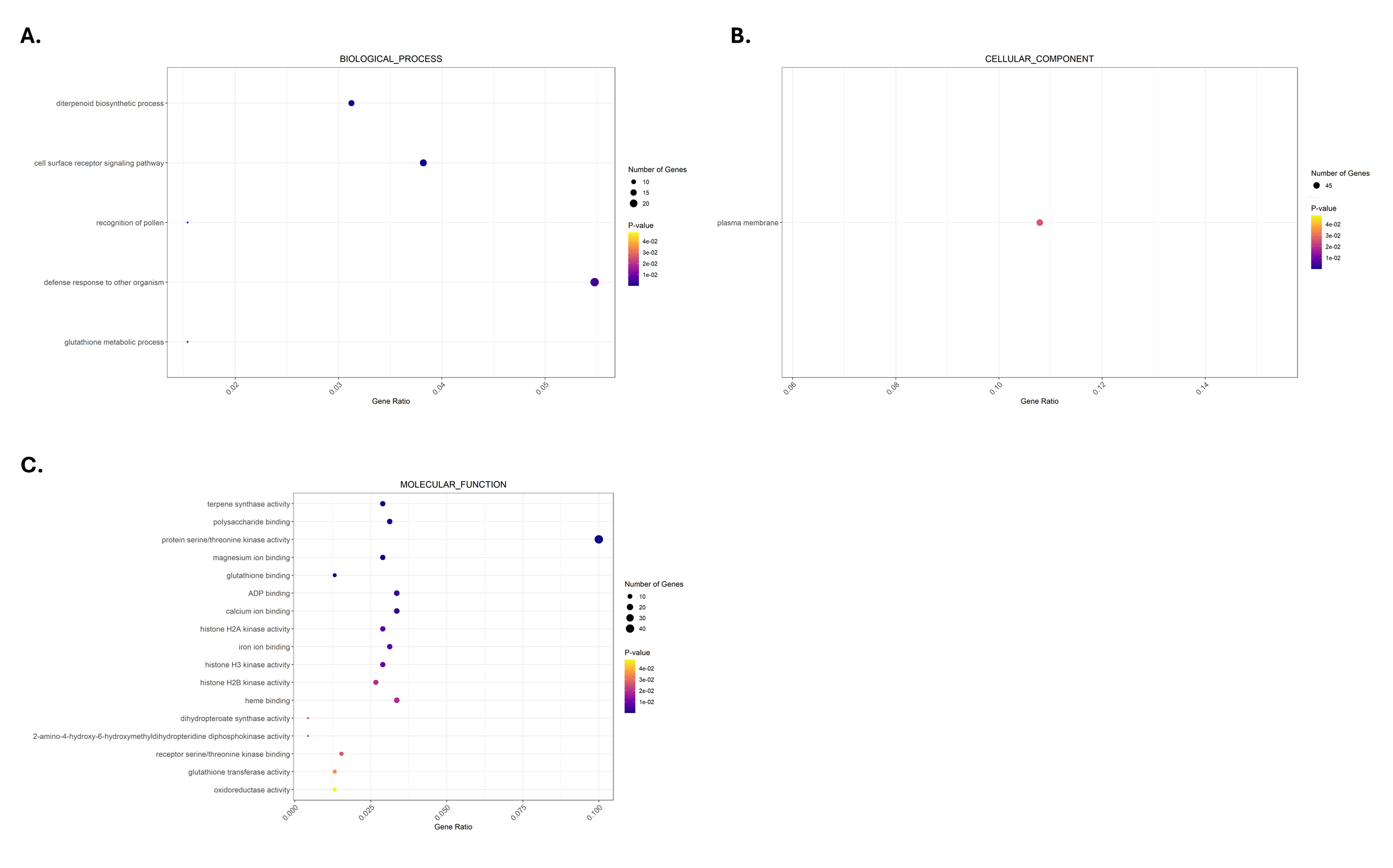

Supplement: giaf142_Supplemental_Files [file giaf142_supplemental_files.zip › Supplementary Fig. S15.PNG]

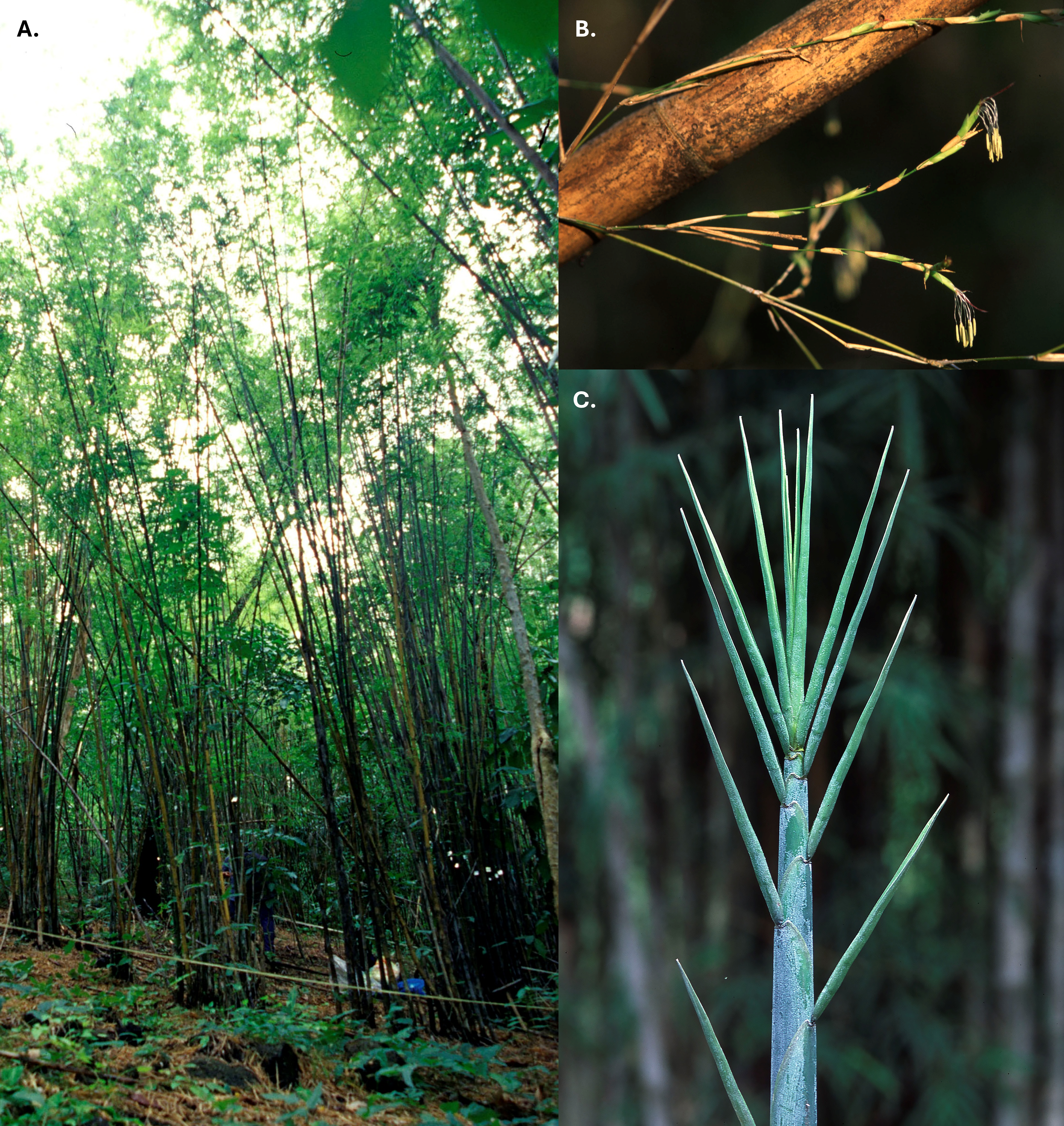

Supplement: giaf142_Supplemental_Files [file giaf142_supplemental_files.zip › Supplementary Fig. S2.PNG]

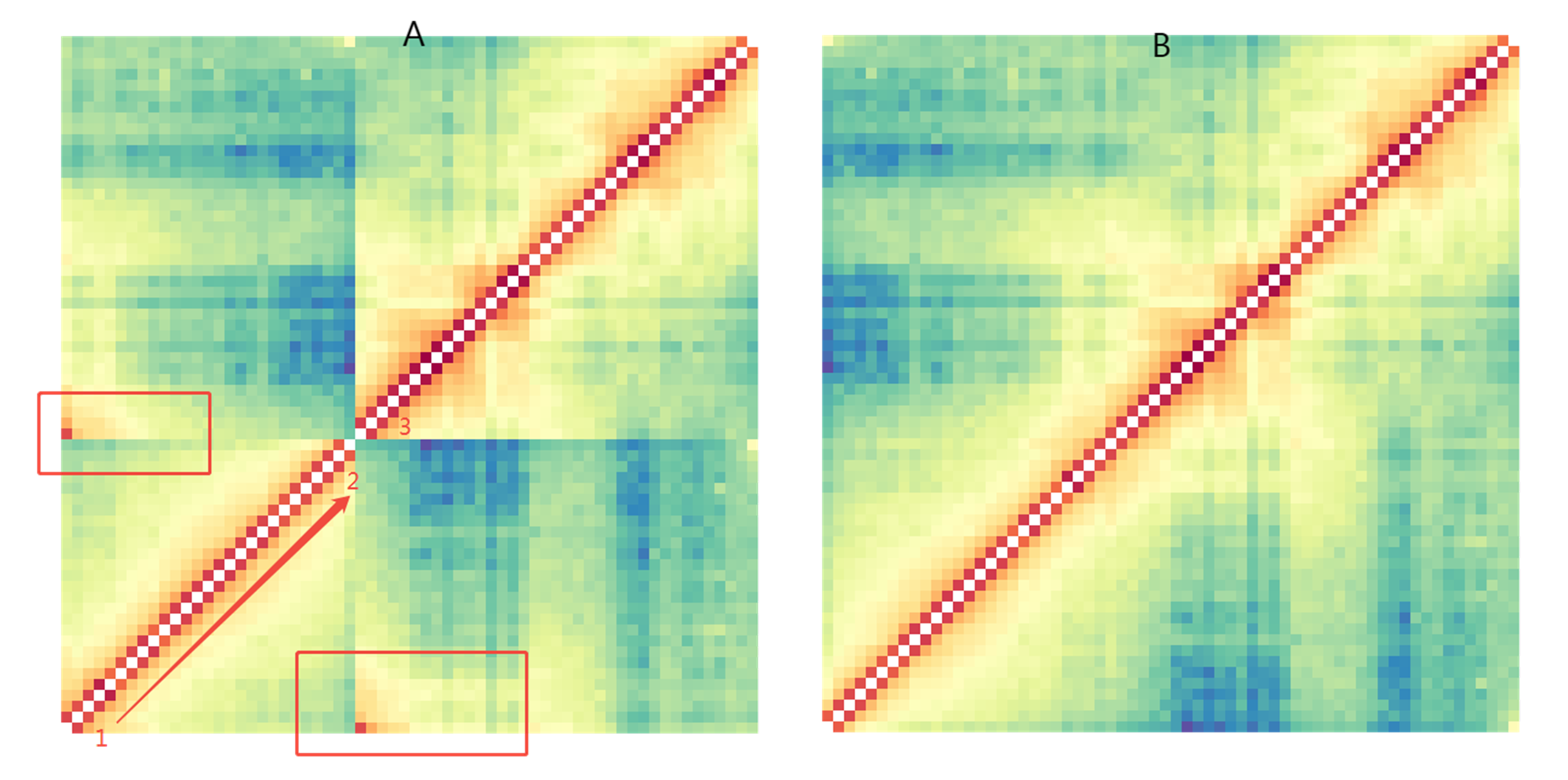

Supplement: giaf142_Supplemental_Files [file giaf142_supplemental_files.zip › Supplementary Fig. S3.png]

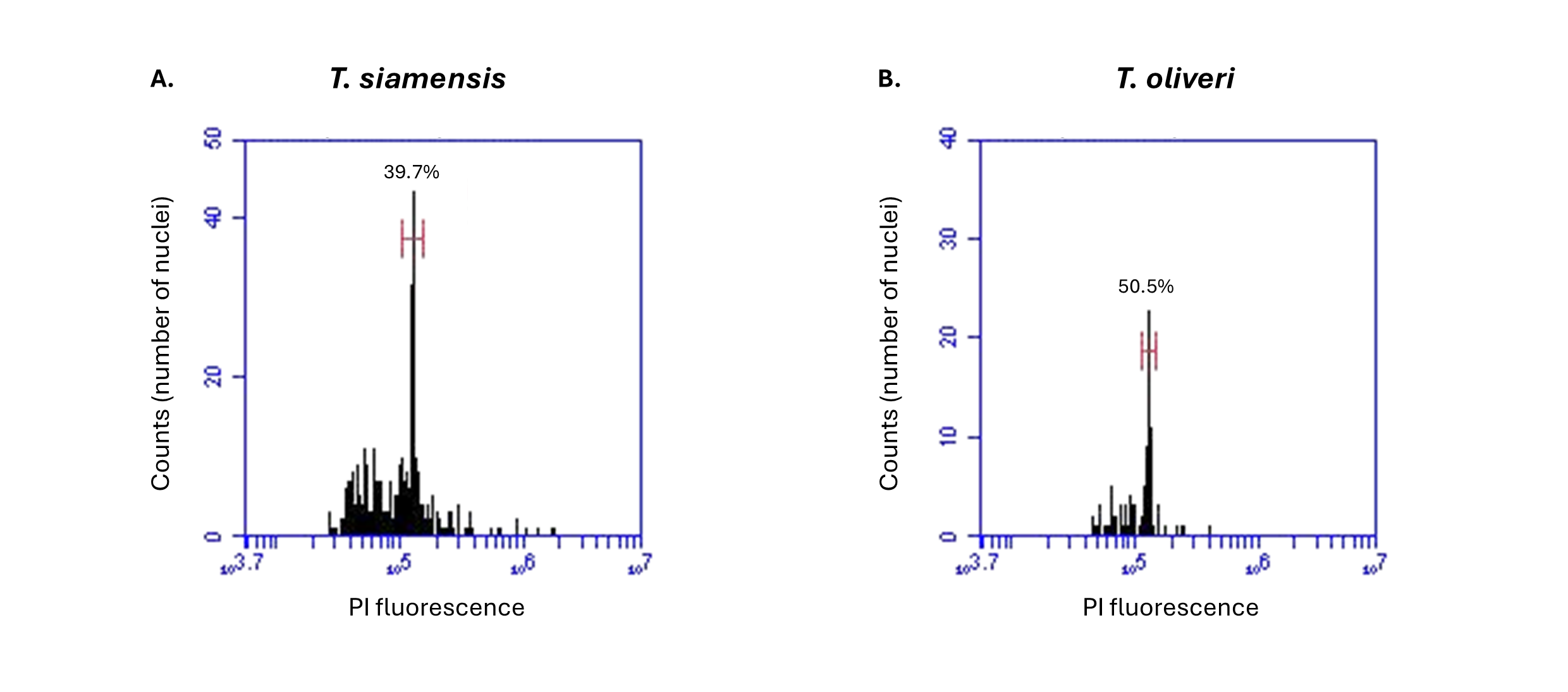

Supplement: giaf142_Supplemental_Files [file giaf142_supplemental_files.zip › Supplementary Fig. S5.PNG]

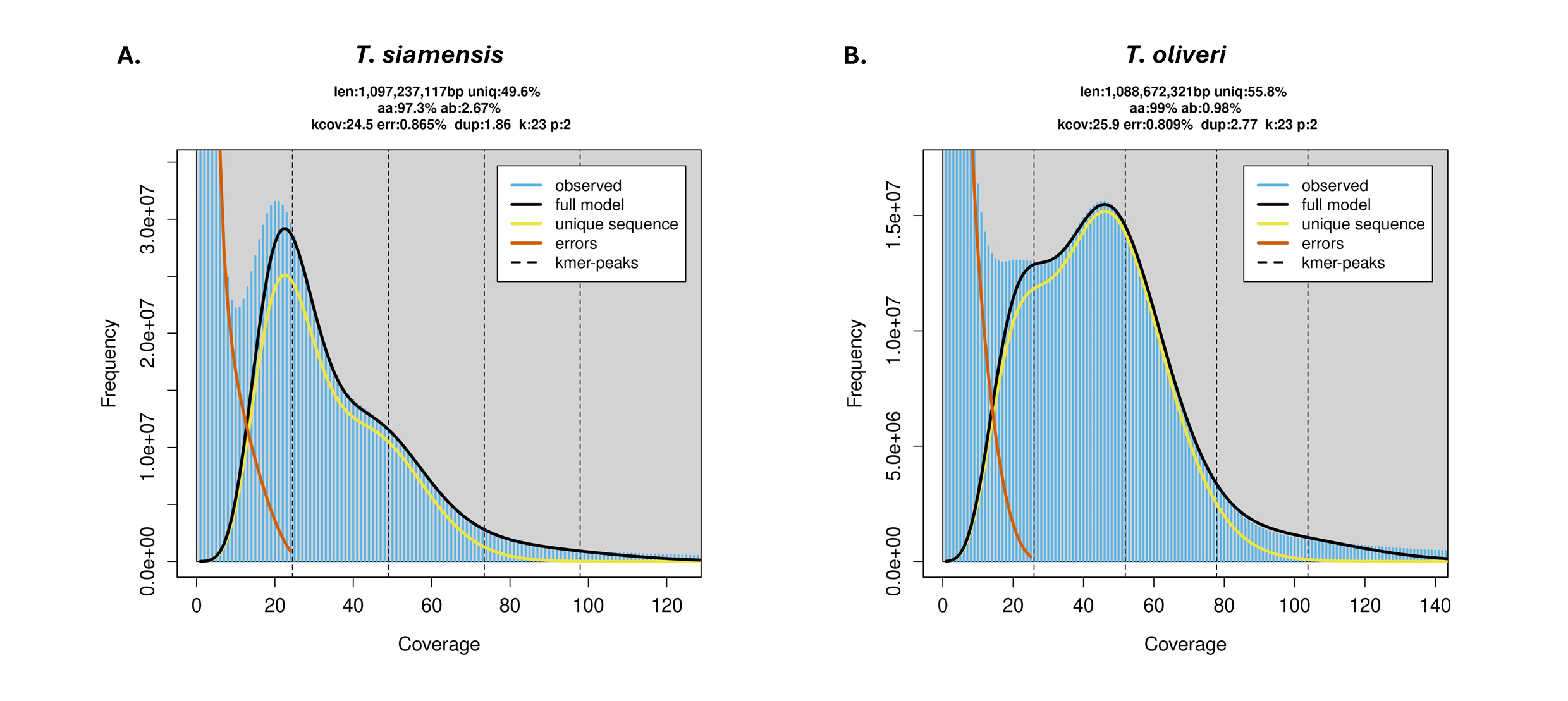

Supplement: giaf142_Supplemental_Files [file giaf142_supplemental_files.zip › Supplementary Fig. S6.png]

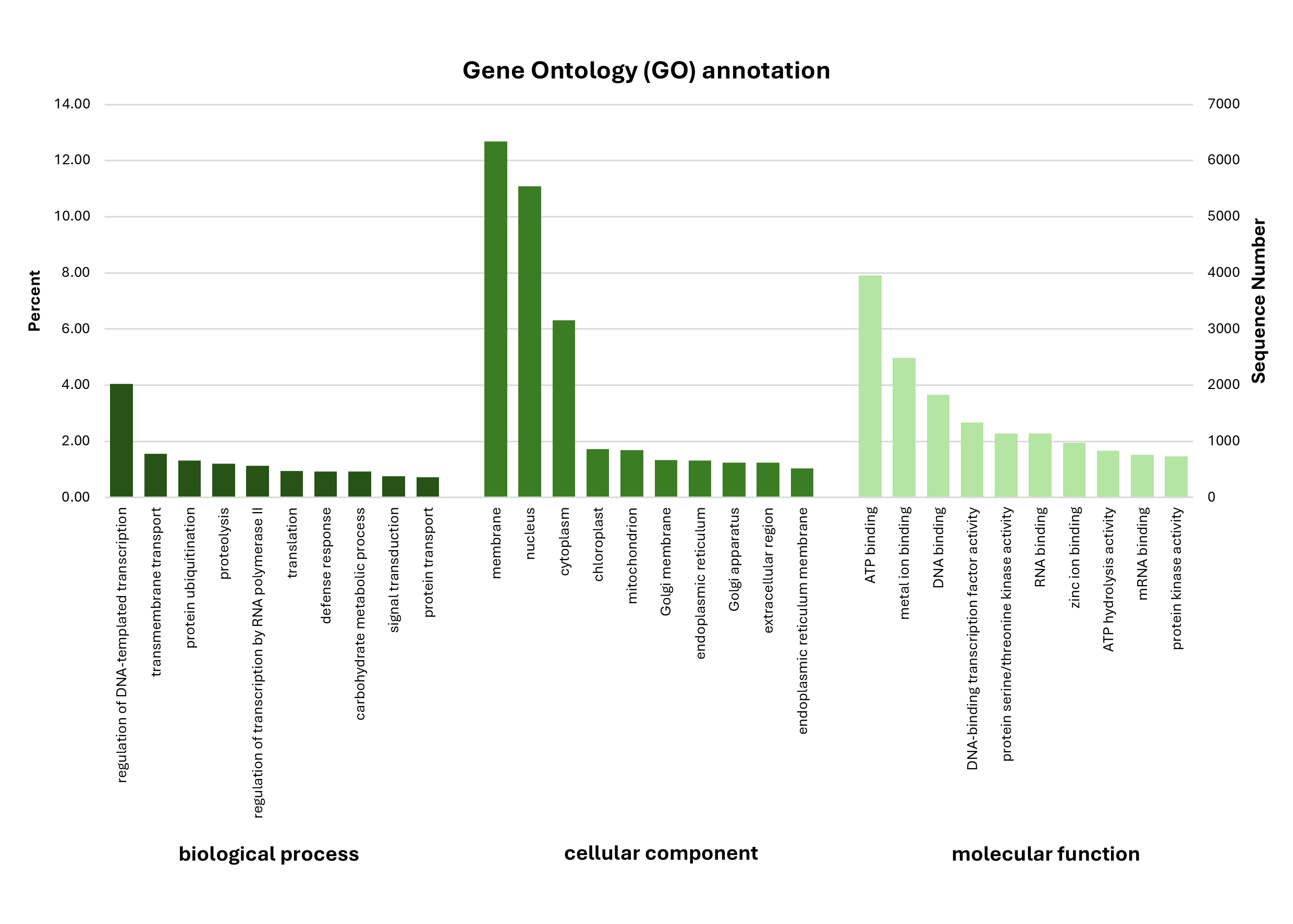

Supplement: giaf142_Supplemental_Files [file giaf142_supplemental_files.zip › Supplementary Fig. S8.PNG]

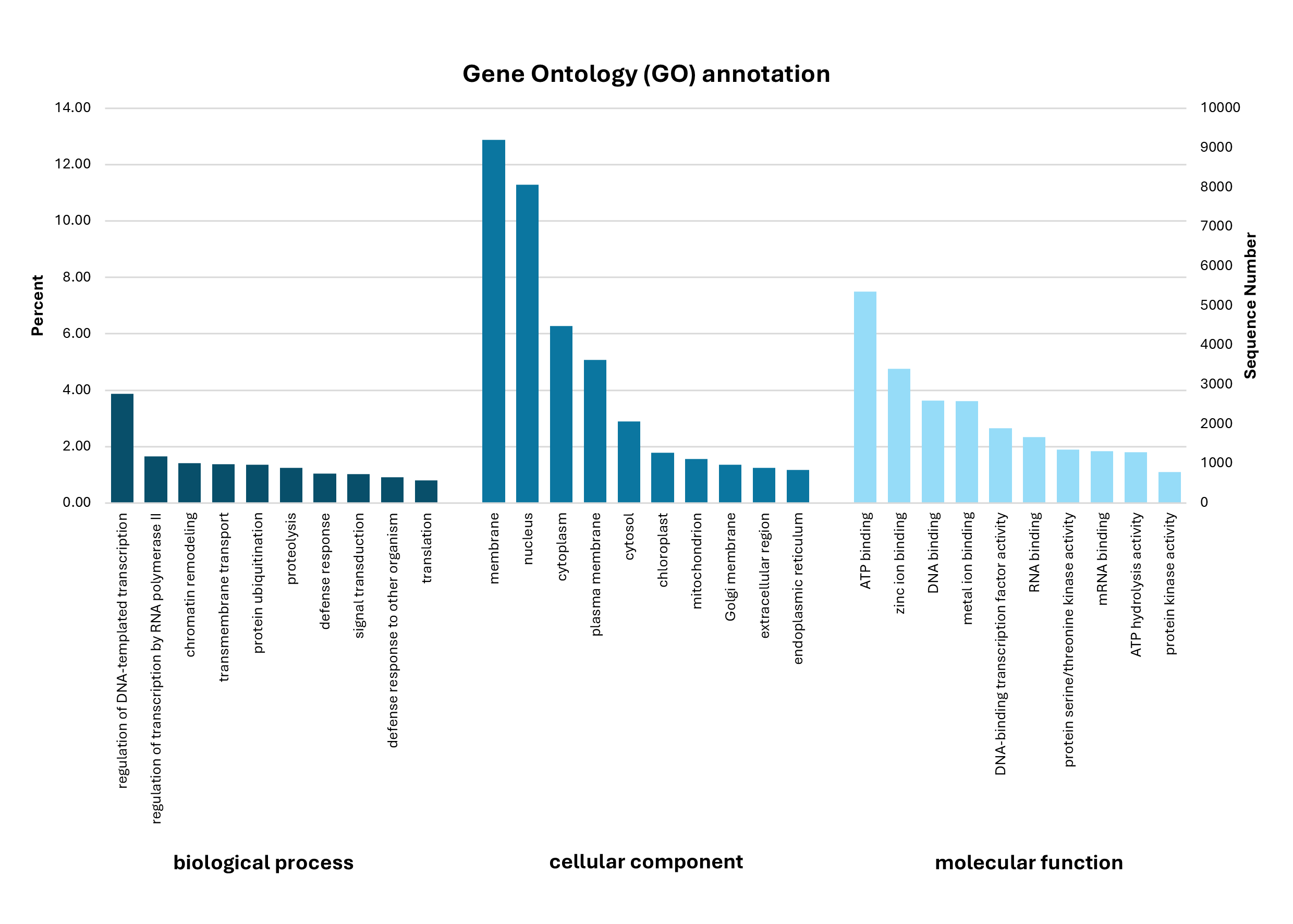

Supplement: giaf142_Supplemental_Files [file giaf142_supplemental_files.zip › Supplementary Fig. S9.png]
