# Supplementary material for: Chromosome-level assemblies of two hexaploid bamboos, Thyrsostachys oliveri and Thyrsostachys siamensis, provide a foundation for functional and comparative genomics studies
Source: Gigascience. 2025 Nov 17;14:giaf142. doi: 10.1093/gigascience/giaf142 (PMC12699669; doi:10.1093/gigascience/giaf142)
Supplement: giaf142_GIGA-D-25-00232_Original_Submission [file giaf142_giga-d-25-00232_original_submission.pdf]

GigaScience

Chromosome-level assemblies of the hexaploid bamboos Thyrsostachys oliveri and Thyrsostachys siamensis enable functional and comparative studies

--Manuscript Draft--

**Manuscript Number:**	GIGA-D-25-00232
**Full Title:**	Chromosome-level assemblies of the hexaploid bamboos Thyrsostachys oliveri and Thyrsostachys siamensis enable functional and comparative studies
**Article Type:**	Research
**Funding Information:**	National Science and Technology Development Agency (P2251271) — Dr. Wirulda Pootakham
**Abstract:**	Background<br><br>Bamboo is an important non-timber forest resource with significant ecological and economic value. However, genomic resources for several bamboo lineages remain scarce.<br><br>Results<br><br>We present the first chromosome-scale reference genomes for two economically important Thai woody bamboos, Thyrsostachys oliveri and Thyrsostachys siamensis. Using single-tube long-fragment read (stLFR) sequencing integrated with chromosome conformation capture (Hi-C) scaffolding, we assembled 35 pseudochromosomes spanning 990Mb (N50 = 22.18Mb) for T. oliveri and 1.14Gb (N50 = 19.45Mb) for T. siamensis, respectively. The T. oliveri and T. siamensis genome assemblies contain 51,191 and 67,483 predicted genes, with repeat contents of 50.9% and 48.8%, respectively. BUSCO completeness scores reached 97.4% for T. oliveri and 95.2% for T. siamensis, indicating high assembly quality for both genomes. Comparative analysis revealed that Thyrsostachys formed a sister group to Dendrocalamus, and the two Thyrsostachys species diverged approximately 5.4 million years ago. Both species have undergone a recent whole-genome duplication event. Gene family analysis identified species-specific gene families associated with root development and carbohydrate metabolism in T. oliveri, and with histone binding and proteasome pathways in T. siamensis. Moreover, homoeolog expression patterns in these hexaploid bamboos showed that homoeolog suppression was more prevalent than dominance, with subgenome-specific expression biases differing between species.<br><br>Conclusions<br><br>These genome assemblies fill a critical gap in bamboo genomics and provide a foundation for evolutionary, functional, and breeding research in hexaploid woody bamboos. They also serve as valuable reference genomes for comparative studies within and across bamboo species.
**Corresponding Author:**	Wirulda Pootakham<br>NSTDA: National Science and Technology Development Agency<br>Khlong Luang, Pathum Thani THAILAND
**Corresponding Author Secondary Information:**	
**Corresponding Author's Institution:**	NSTDA: National Science and Technology Development Agency
**Corresponding Author's Secondary Institution:**	
**First Author:**	Chaiwat Naktang
**First Author Secondary Information:**	
**Order of Authors:**	Chaiwat Naktang

	Supaporn Khanbo
	Poompat Phadphon
	Sonicha U-thoomporn
	Duangjai Sangsakru
	Chutima Sonthirod
	Pitchaporn Waiyamitra
	Sarawood Sungkaew
	Sithichoke Tangphatsornruang
	Wirulda Pootakham
**Order of Authors Secondary Information:**	
**Additional Information:**	
**Question**	**Response**
Are you submitting this manuscript to a special series or article collection?	No
**Experimental design and statistics**<br><br>Full details of the experimental design and statistical methods used should be given in the Methods section, as detailed in our Minimum Standards Reporting Checklist. Information essential to interpreting the data presented should be made available in the figure legends.<br><br>Have you included all the information requested in your manuscript?	Yes
**Resources**<br><br>A description of all resources used, including antibodies, cell lines, animals and software tools, with enough information to allow them to be uniquely identified, should be included in the Methods section. Authors are strongly encouraged to cite Research Resource Identifiers (RRIDs) for antibodies, model organisms and tools, where possible.<br><br>Have you included the information requested as detailed in our Minimum Standards Reporting Checklist?	Yes

Powered by Editorial Manager® and ProduXion Manager® from Aries Systems Corporation

**Availability of data and materials**<br><br>All datasets and code on which the conclusions of the paper rely must be either included in your submission or deposited in publicly available repositories (where available and ethically appropriate), referencing such data using a unique identifier in the references and in the "Availability of Data and Materials" section of your manuscript.<br><br>Have you have met the above requirement as detailed in our Minimum Standards Reporting Checklist?	Yes
GigaScience has policies and guidelines in place for the use of generative AI-writing tools such as ChatGPT. If you have used such writing tools to assist with writing the manuscript this must be declared and cited in the text. Authors should not list AI-writing tools and other AI-assisted technologies as an author or co-author and should acknowledge that they are fully responsible for text generated or refined by AI-writing tools.<p><br><br>A summary of use (particularly in the introduction or among methods) needs to be included at the end of the paper, and the outputs should also be included as a supplementary file hosted in GigaDB or other open repositories. Please read our guidelines for more information. <p><br><br>By submitting to GigaScience, you are aware of the journal's AI-writing tools policy, and if you have declared use of such tools below, you have acknowledged this where appropriate in your manuscript and have made a summary of use and outputs available. <p><br><b>AI-assisted writing tools have been used in the preparation of this manuscript?	No

Manuscript

Click here to access/download;Manuscript;Bamboo_Genome_Manuscript_gi

Chromosome-level assemblies of the hexaploid bamboos *Thyrsostachys oliveri* and *Thyrsostachys siamensis* enable functional and comparative studies

Chaiwat Naktang[1, †], Supaporn Khanbo[1, †], Poompat Phadphon[1], Sonicha U-thoomporn[1], Duangjai Sangsakru[1], Chutima Sonthirod[1], Pitchaporn Waiyamitra[1], Sarawood Sungkaew[2], Sithichoke Tangphatsornruang[1], Wirulda Pootakham[1,*]

[1]National Omics Center, National Center for Genetic Engineering and Biotechnology, National Science and Development Agency, Pathum Thani, Thailand

[2]Department of Forest Biology, Faculty of Forestry, Kasetsart University, Bangkok, Thailand

[†]Equal contribution and shared first authorship.

*Corresponding author:

Wirulda Pootakham (wirulda@alumni.stanford.edu)

Abstract

Background

Bamboo is an important non-timber forest resource with significant ecological and economic value. However, genomic resources for several bamboo lineages remain scarce.

Results

We present the first chromosome-scale reference genomes for two economically important Thai woody bamboos, *Thyrsostachys oliveri* and *Thyrsostachys siamensis*. Using single-tube long-fragment read (stLFR) sequencing integrated with chromosome conformation capture (Hi-C) scaffolding, we assembled 35 pseudochromosomes spanning 990 Mb (N50 = 22.18 Mb) for *T. oliveri* and 1.14 Gb (N50 = 19.45 Mb) for *T. siamensis*, respectively. The *T. oliveri* and *T. siamensis* genome assemblies contain 51,191 and 67,483 predicted genes, with repeat contents of 50.9% and 48.8%, respectively. BUSCO completeness scores reached 97.4% for *T. oliveri* and 95.2% for *T. siamensis*, indicating high assembly quality for both genomes. Comparative analysis revealed that *Thyrsostachys* formed a sister group to *Dendrocalamus,* and the two *Thyrsostachys* species diverged approximately 5.4 million years ago. Both species have undergone a recent whole-genome duplication event. Gene family analysis identified species-specific gene families associated with root development and carbohydrate metabolism in *T. oliveri*, and with histone binding and proteasome pathways in *T. siamensis*. Moreover, homoeolog expression patterns in these hexaploid bamboos showed that homoeolog suppression was more prevalent than dominance, with subgenome-specific expression biases differing between species.

Conclusions

These genome assemblies fill a critical gap in bamboo genomics and provide a foundation for evolutionary, functional, and breeding research in hexaploid woody bamboos. They also serve as valuable reference genomes for comparative studies within and across bamboo species.

Keywords: *Thyrsostachys oliveri*, *Thyrsostachys siamensis,* chromosome-scale genome assembly, Annotation, Hi-C, stLFR

Introduction

Bamboo is one of the most important non-timber forest resources that are widely distributed across the subtropical and tropical regions of Asia, Africa, and Latin America [1-3], covering roughly 31–35 million ha (≈ 0.8–1% of global forest area) [4, 5]. Bamboos constitute the subfamily Bambusoideae of Poaceae (Gramineae) and include about 1,670 species in 125 genera [3, 6, 7]. They are classified into four monophyletic lineages with distinct ploidy levels: herbaceous bamboos (2n = 20–24, diploid); temperate woody bamboos (2n = 46–48, tetraploid); neotropical woody bamboos (2n= 40–48, tetraploid); and palaeotropical woody bamboos (2n = 70–72, hexaploid) [8-11]. Among these, woody bamboos show a wide range in genome sizes, chromosome number, and ploidy [8]. Because of rapid growth and ease of propagation, bamboos provide substantial economic and ecological benefits. Their uses span food, medicine, bio-energy, building timber, furniture, and handicrafts, and they also help restore degraded landscapes and mitigate climate change impacts [12-14].

Bamboo plays a significant economic role in many developing countries, particularly in Asia [15]. In Thailand, roughly 69 species across 17 genera have been recorded [14, 16]. *Thyrsostachys* is a small bamboo genus native to Thailand and Myanmar, comprising only two species*: Thyrsostachys oliveri* and *Thyrsostachys siamensis* [17, 18]. *T. oliveri* is a tropical clumping bamboo with straight, small-diameter culms that are valued locally for construction [19]. *T. siamensis* is one of the most useful Thai bamboos; it grows rapidly, produces edible shoots, and supplies material for construction and handicrafts [14, 20]. Both species have tall, upright canes and broad socio-economic importance, yet genomic information for them remains scarce.

Genome assembly and annotation are essential for revealing functional elements that underpin breeding and conservation efforts. Although high-quality assemblies have recently been generated for *Dendrocalamus brandisii* [21], *Dendrocalamus latiflorus* [22], *Phyllostachys edulis* [13], *Phyllostachys heterocycla* [23], and 11 additional bamboo species published in 2024 [24] , a reference genome for *Thyrsostachys* has been lacking. This gap limits deeper insights into bamboo genome evolution and functional traits.

Here, we sequenced and assembled two Thai hexaploid species, *T. oliveri* and *T. siamensis*, using single-tube long-fragment reads (stLFR) technology [25] combined with chromosome conformation capture (Hi-C) scaffolding. The resulting chromosome-level assemblies provide crucial resources for investigating genomic organization and evolution in these species. Our work fills a critical gap in bamboo genomics and lays the groundwork for future studies aimed at genetic improvement, molecular breeding, and conservation of economically important bamboos.

Methods

Plant materials and DNA/RNA isolation

For genome sequencing, young leaf samples were collected from *T. oliveri* and *T. siamensis* plants grown at Kasetsart University, Bangkok Province, Thailand (13.8423° N, 100.5771° E). Immediately after collection, healthy leaves were flash-frozen in liquid nitrogen and stored at -80°C. High-molecular-weight (HMW) DNA was extracted using the Qiagen Genomic-tip 100/G following the manufacturer’s instructions (Qiagen, Hilden, Germany). DNA quality and quantity were subsequently evaluated using the Pippin Pulse Electrophoresis System (Sage Science, Beverly, USA) and the Qubit 4 Fluorometer (Thermo Fisher Scientific, Waltham, USA), respectively, prior to library construction. For downstream annotation purposes, total RNA was extracted from leaf and root tissues of the same individual used for RNA sequencing, following the protocol outlined in [26]. Briefly, RNA was isolated using a CTAB buffer and a 25:24:1 phenol:chloroform:isoamyl alcohol mixture, then precipitated overnight with a quarter volume of 8M LiCl. The resulting RNA pellets were washed with 70% ethanol, air-dried, and resuspended in RNase-free water. RNA integrity was assessed using the Fragment Analyzer system (Agilent, Santa Clara, USA) before RNA sequencing library construction.

Genome and transcriptome sequencing

A preliminary draft genome assembly was generated by first constructing a stLFR (single-tube long fragment read) sequencing library. This library was prepared using 10 ng of high-molecular-weight DNA and the MGIEasy stLFR Library Prep Kit (MGI Tech, Shenzhen, China), following the manufacturer's protocol. Briefly, this process involved transposon-mediated insertion into the HMW DNA, incubation with

clonally barcoded beads for random priming, and subsequent fragmentation into sub-fragments (<1 kb). Following adapter ligation and PCR amplification, the library was ready for sequencing. For transcriptome analysis, 200 ng of total RNA was used to generate a library with the MGIEasy RNA Library Prep Kit v3.0 (MGI Tech, Shenzhen, China), adhering to the manufacturer's instructions. Both the stLFR and RNA libraries were then sequenced on the DNBSEQ-G400 platform using the MGISEQ-2000RS Sequencing Flow Cell v3.0 (MGI Tech, Shenzhen, China).

Hi-C library preparation and sequencing

To achieve chromosome-level scaffolding of the initial assembly, Biomarker Technologies (Beijing, China) utilized a chromosome conformation capture (Hi-C) technique. For Hi-C library preparation using *T. oliveri* tissue, the following procedure was employed. Briefly, chromatin from fresh tissue was crosslinked with formaldehyde, and the fixed chromatin was digested with the restriction endonuclease HindIII [27]. The resulting fragments were then treated to incorporate biotinylated nucleotides at their 5′ ends, followed by ligation to form chimeric junctions representing spatially proximate chromatin regions. After reversing the crosslinks, the DNA was purified and sheared into fragments ranging from 300 to 700 base pairs. Biotinylated fragments were then enriched using streptavidin beads. The purified Hi-C fragments were used to construct Illumina-compatible sequencing libraries, which were subsequently sequenced on the Illumina HiSeq X Ten (PE150; Illumina, San Diego, CA, USA).

Genome assembly and Hi-C scaffolding

Preliminary drafts assemblies for both genomes were generated from 150-bp paired-end stLFR sequencing data using stLFRdenovo v1.0.5 with default settings (https://github.com/BGI-biotools/stLFRdenovo/releases/tag/v1.0.5, accessed on 1 March 2025). Subsequently, the initial draft assembly of *T. oliveri* was scaffolded to chromosome level utilizing Hi-C data. This Hi-C scaffolding was conducted by Biomarker Technologies Corporation (Beijing, China). The draft assembly and Hi-C reads were processed using HiRise v2.1.9, a pipeline optimized for proximity ligation data [28]. Hi-C reads were aligned to the draft assembly using BWA v0.7.17 [29] while data filtering and quality assessment were performed with HiC-Pro v2.10.0 [30]. The HiRise software analyzed Hi-C read pair distributions to detect and correct misjoins, identify prospective joins, and produce a chromosome-level assembly. Manual inspection and Hi-C visualization tools were employed to further refine the assembly. *T. siamensis* was subsequently scaffolded using the *T. oliveri* chromosome-level assembly as a reference with the RagTag software v1.1.0 (https://github.com/malonge/RagTag; last accessed: 7 February 2025) [31] . Both *T. oliveri* and *T. siamensis* genome assemblies have been deposited at NCBI under accession numbers JAWCWW000000000 and JBEFOJ000000000, respectively. The raw stLFR reads for *T. oliveri* and *T. siamensis* are available in the NCBI SRA database under accession numbers SRR32915794 and SRR32915889, respectively. The transcriptome data for *T. oliveri* were submitted under SRR33015685 and SRR33015684. The transcriptome data for *T. siamensis* were submitted under SRR33015804 and SRR33015803.

Genome size estimation

Genome size was estimated using two complementary approaches. First, the k-mer analysis was performed on raw stLFR reads using Jellyfish v2.2.10, and the resulting distributions were visualized with GenomeScope v2.0 [32] (k = 21; http://genomescope.org/genomescope2.0/; accessed March 18, 2025). Second, nuclear DNA content was estimated using flow cytometry. Fresh leaf tissues from *T. oliveri* and *T. siamensis* were processed following the protocol described in [33], using Galbraith's buffer [34] for nuclear isolation. Nuclei were stained with 50 µg/mL propidium iodide (Thermo Fisher Scientific). Maize (*Zea mays*) leaf sample was used as the reference standard for DNA content.

Quality assessment of the genome assembly

To evaluate the quality of the final *T. oliveri* and *T. siamensis* assemblies, both short-read DNA (stLFR) and RNA sequencing data were mapped to each respective assembly. Specifically, stLFR reads were aligned using BWA v0.7.17 [29], and RNA sequencing reads were aligned using HISAT2 v2.2.0 [35], Furthermore, assembly completeness was assessed using the Benchmarking Universal Single-Copy Orthologs (BUSCO) pipeline v5.4.4 [36], which examined the presence of conserved orthologous genes against the plant-specific Embryophyta OrthoDB database release 10 [37].

Repetitive sequence identification

Repetitive elements were identified through both de novo and homology-based approaches. First, we generated a de novo repeat library using RepeatModeler (v2.0.3) [38], which incorporates multiple algorithms (RECON, RepeatScout and LtrHarvest/Ltr_retriever) to detect and characterize repeat boundaries [39, 40]. The resulting consensus sequences were aligned against the NCBI GenBank non-

redundant protein database (nr) via BLASTX (e-value ≤ $1e^{-6}$) to confirm the absence of large, non-TE protein-coding families. For homology-based identification, the assembled genome was scanned with RepeatMasker v4.0.9_p2 [41] using the RepBase plant repeat database [42].

Gene prediction and annotation

To annotate protein-coding sequences, we employed both Evidence Modeler (EVM) software version 1.1.1 [43] and the MAKER2 pipeline [44] to identify protein-coding genes in the masked genome assembly. These approaches combined homology-based, RNA-based, and ab initio predictions. Transcript-based gene prediction was performed using RNA-seq data derived from leaf and root tissues. Raw reads were assembled using Trinity v2.9.1 [45] and clustered at 95% identity with CD-HIT v4.8.1 [46, 47]. For each cluster, the longest open reading frame (ORF) was identified and subsequently aligned to the assembled genome using PASA v2.5.3 [48] and GMAP v2020-09-12 [49]. To aid in gene annotation, protein sequences from related bamboo species, specifically *Dendrocalamus latiflorus* (forestry.fafu.edu.cn/pub/Dla), *Dendrocalamus sinicus*, *Bonia amplexicaulis*, *Guadua angustifolia*, *Olyra latifolia*, and *Raddia guianensis* (all accessible via https://genomevolution.org/coge), were downloaded from publicly available databases and aligned to the genome assembly using AAT [50]. Ab initio gene predictions were generated with AUGUSTUS v3.2.1 [51], trained on data from the same reference species plus PASA transcript alignments. EVM was then used to integrate these lines of evidence—transcript, homology, and ab initio—assigning weights of 5 for PASA, 1 for GMAP, 0.5 for AAT, and 0.1 for AUGUSTUS.

In parallel, the MAKER2 pipeline was employed to merge ab initio predictions (using SNAP [52] and AUGUSTUS), protein homology, and transcript-based evidence. After an initial MAKER run with default parameters, the resulting gene models were used to retrain SNAP and AUGUSTUS, followed by a second MAKER iteration to refine predictions. Low-confidence models were removed or flagged for manual inspection, and the final consensus gene models were output in GFF format. GFF files exhibiting the highest BUSCO completeness scores were ultimately selected for downstream analyses, ensuring robust and high-quality gene annotations. All predicted genes were functionally annotated using OmicsBox (v2.0.10) (https://www.biobam.com/download-omicsbox/; last accessed on [20 March 2025]). Protein sequences were aligned against the GenBank's non-redundant (NR) databases via BLASTP [53] with an e-value cutoff of 10^{-5}. Gene Ontology (GO) terms were retrieved and assigned to the predicted gene models, while enzyme codes (EC) were extracted and mapped to KEGG pathway annotations. To annotate noncoding RNAs (ncRNAs), tRNAs were predicted using tRNAscan-SE v2.0 [54], and rRNA sequences were identified by aligning known rRNA references from closely related species using BLAST. Other ncRNAs, such as miRNAs and snRNAs, were identified by searching against the Rfam v14.1 database [55] with Infernal v1.1.5 [56] using default parameters.

Comparative genomics and phylogenetic analyses

To investigate the evolutionary relationships of *T. oliveri* and *T. siamensis* within the grass family, a comparative genomics study was conducted. OrthoFinder v2.5.5 [57] was used to identify orthologous groups among ten grass species, including nine bamboo species (*B. amplexicaulis*, *G. angustifolia*, *P. edulis* (Moso bamboo), *T. oliveri*,

T. siamensis, *O. latifolia*, *R. guianensis, D. sinicus* and *D. latiflorus*), other grasses (*Brachypodium distachyon* (a model grass species), and a cereal crop (*Oryza sativa* (rice)). A set of single-copy orthologous proteins was extracted, and these sequences were aligned and trimmed using MUSCLE v3.8 [58] and trimAl [59], respectively. Catsequences software (https://github.com/ChrisCreevey/catsequences) was used to concatenate the alignment blocks. The best substitution model for each block was evaluated using ModelTest-NG [60]. A maximum-likelihood phylogenetic tree was constructed using RAxML-NG [61] with 1000 bootstrap. *O. sativa* was designated as an outgroup.

The divergence time of the 11 species was estimated using BEAST v.2.7.7 [62]. Two independent MCMC tree searches were run for 10,000,000 generations, with a sampling frequency of 1,000 generations. The estimations were run under JTT substitution model together with optimized relaxed clock model and Birth-Death tree prior. The Bambusoideae cf. Chusquea fossil (35-90 Mya;[63]) and the fossilized phytoliths and cuticle of Ehrhartoideae-Oryzeae (67-90 Mya;[64]) were used to calibrate the crown node of Bambusoideae and BEP clade respectively following [65]. The crown node of Bambuseae estimated to be 28.24 (19.82-38.95) Mya by Zhang et al. (2016) was included as a secondary calibration point. To assess the convergence of parameters, Tracer v1.7.1 [66] was utilized to check the effective sample sizes (ESSs). ESSs of all parameters were above 200 after a burn-in period of 25%. The maximum clade credibility tree was generated by TreeAnnotator v2.7.7 [62].

Gene family expansions and contractions were identified using CAFE v5 [67], to detect significant changes in gene family size across the phylogeny ($p < 0.05$). This analysis employed a probabilistic model based on a time-calibrated phylogenetic tree and estimated gene birth-death rates (λ) using a maximum-likelihood approach.

Functionally, Gene Ontology (GO) analyses were performed to annotate expanded and contracted gene families.

The analysis of genome synteny

Collinearity analyses were performed using McscanX [68] to investigate syntenic relationships within the *T. oliveri* and *T. siamensis* genomes, and between *T. oliveri* and *T. siamensis*, *B. amplexicaulis*, *D. sinicus*, *D. latiflorus*, *O. latifolia*, *P. edulis*, and *O. sativa*. Putative paralogous gene pairs were identified by aligning *T. oliveri* amino acid sequences against themselves and those of *T. siamensis* using BLASTP with an E-value threshold of 10^{-10}. Intragenic homeologous blocks were defined as regions containing ten or more collinear or nearly collinear paralogous gene pairs, with a maximum of six intervening non-paralogous genes. The resulting intragenic homeologous blocks were visualized using CIRCOS v0.69.8 [69]. The synteny of Thyrsostachys subgenome and *Oryza sativa* was examined using NGenomeSyn 1.41 [70] to identify similarities in their genomic structures.

Subgenome Identification

To facilitate subgenome classification within the newly assembled *T. oliveri* and *T. siamensis* genomes, the established subgenome assignments of the *D. sinicus* reference genome, previously determined via allele-aware chromosome-level analysis [71] , were adopted as a reference framework. Briefly, protein-coding genes from the *T. oliveri* and *T. siamensis* assemblies were aligned to those of the *D. sinicus* reference using the jcvi synteny pipeline (v1.1.17) [72]. Syntenic gene blocks were identified, with the '–quota' parameter adjusted to reflect genome ploidy. By comparing the resulting syntenic blocks to the previously characterized subgenomes of *D. sinicus*, subgenome identities were inferred for the *T. oliveri* and *T. siamensis* assemblies. This

methodology enabled the projection of the established *D. sinicus* subgenome structure onto the target genomes, thereby minimizing methodological redundancy.

Expression bias between subgenomes

To investigate subgenome-specific gene expression in hexaploid *T. oliveri* and *T. siamensis*, we utilized 1:1:1 gene triads, identified using the methodology of [24]. Briefly, we defined a triad as expressed when the sum of the A, B and C subgenome homoeologs had transcript per million (TPM)> 0.5 and standardized the relative expression of each homoeolog across the triad. The ternary diagrams were plotted using the R package ggtern [73]. To categorize homoeolog expression bias, we employed a method analogous to that used in wheat [74]. Ideal normalized expression biases for six distinct categories were defined. The Euclidean distance (calculated using the R function rdist) between the observed normalized expression of each triad and each ideal category was determined. Each triad was subsequently assigned to the category with the shortest Euclidean distance, representing its homoeolog expression bias, and this process was repeated for for both leaf and root tissues.

Results

Genome assembly and evaluation

High-coverage stLFR libraries generated 408,544,881 reads (81.71 Gb) for *T. oliveri* and 401,766,290 reads (80.35 Gb) for *T. siamensis* (Table S1). Genome size estimates for *T. oliveri* were 1.088 Gb by k-mer analysis and 1.226 Gb by flow cytometry, yielding an average of 1.157 Gb; for *T. siamensis*, estimated were 1.097 Gb (k-mer) and 1.247 Gb (flow cytometry), with a mean of 1.172 Gb (Fig. S1–S2). These values correspond to ~71× and ~69× physical coverage, respectively. De novo

assembly of the stLFR reads produced 32,505 contigs (N50 = 2.45 Mb; 990 Mb) for *T. oliveri* and 77,021 contigs (N50 = 0.32 Mb; 1.14 Gb) for *T. siamensis* (Table 1). Heterozygosity estimates were 0.98% for *T. oliveri* and 2.67% for *T. siamensis* (Fig. S2).

For *T. oliveri*, 351 million Hi-C read pairs raised the scaffold N50 to 22.18 Mb and anchored 81.4% (804 Mb) of sequence into 35 pseudo-chromosomes, matching 2n = 6x = 70 (Fig. 1A and Fig. S7). For *T. siamensis*, RagTag scaffolding against the *T. oliveri* reference produced a 19.45 Mb scaffold N50 with 69.2% (789 Mb) of sequence anchored (Table 1). Each assembly was partitioned into three homoeologous subgenomes (A, B, C). Extensive intra- and inter-subgenomic collinearity and large blocks aligning to the 12 *O. sativa* chromosomes are shown in Fig. 1A-C.

BUSCO analysis (Embryophyta odb10; 1,614 genes) recovered 97.4% complete genes in *T. oliveri* and 95.2% in *T. siamensis* (Table 1). Read-mapping rates further supported assembly accuracy: 98.83% / 98.10% of stLFR reads and 90.22% / 87.62% of RNA-seq reads mapped back to the *T. oliveri* and *T. siamensis* genomes, respectively. Together, these results confirm that both *Thyrsostachys* genomes are high-quality, chromosome-scale resources suitable for comparative and functional genomics.

Table 1. Assembly statistics of *T. oliveri* and *T. siamensis*

	T. oliveri	***T. siamensis***

	stLFR	Hi-C	stLFR	RagTag
N50 scaffold size (bases)	2,447,080	22,175,826	319,778	19,454,152
L50 scaffold number	86	18	390	23
N75 scaffold size (bases)	407,938	15,134,499	13,857	34,829
L75 scaffold number	313	31	7,916	629
N90 scaffold size (bases)	9,250	9,256	4,892	6,105
L90 scaffold number	5,845	4,941	29,083	15,413
Total (bases)	990,067,507	990,098,900	1,138,811,672	1,140,691,237
Number of scaffolds	32,505	31,592	77,021	57,746
Number of scaffolds ≥ 100 kb	566	51	964	119
Number of scaffolds ≥ 1 Mb	187	38	139	49
Number of scaffolds ≥ 10 Mb	10	35	5	36
Longest scaffold (bases)	15,491,168	40,971,832	14,091,541	40,067,183
GC content (%)	43.41	43.41	43.19	43.19
BUSCO evaluation (% completeness)	97.4	97.4	94.6	95.2

Genome annotation

To annotate the *Thyrsostachys* genomes, we employed an integrated pipeline combining ab initio prediction, RNA-seq supported evidence, and protein homology evidence. This analysis revealed distinct differences in the genomic composition of the two bamboo species. *T. oliveri* contained 51,191 predicted gene models, of which 48,070 were protein-coding; *T. siamensis* exhibited 67,483 predicted models with

59,683 protein-coding genes. Average genomic GC content was similar between species (43.41 % in *T. oliveri*, 43.19 % in T. *siamensis*), with exons enriched in GC (53.4 % and 54.7 %, respectively) and introns lower (39.8 % and 39.0 %) (Table 2).

Table 2. Annotation statistics for *T. oliveri* and *T. siamensis*

	T. oliveri	***T. siamensis***
Number of predicted gene models	51,191	67483
Total gene length (Mb)	146.26	179.99
Average gene size (nt)	2857	2667
Average number of exons/gene	5.04	4.27
Total exon length (Mb)	55.98	71.55
Average exon length (nt)	216.8	248.2
GC content of exons (%)	53.4	54.69
Average number of Introns/gene	4.04	3.27
Total intron length (Mb)	90.33	108.51
Average intron length (nt)	436.4	491.5
GC content of introns (%)	39.81	38.99

Of these protein-coding genes, 93.9 % in *T. oliveri* and 88.4 % in *T. siamensis* had best hits in the NCBI non-redundant (NR) database. GO classifications were obtained for 72.4 % and 77.8 % of genes, respectively, and 32.2 % versus 28.1 % were assigned to at least one KEGG pathway (Table S2).

GO terms were assigned to 36,541 genes in *T. oliveri* and 52,475 genes in *T. siamensis* (Table S2). In the biological-process category, *T. oliveri* was enriched for

regulation of DNA-templated transcription, transmembrane transport, and protein ubiquitination, whereas *T. siamensis* showed regulation of DNA-templated transcription, regulation of transcription by RNA polymerase II, and chromatin remodeling. Membrane, nucleus, and cytoplasm dominated the cellular-component category in both species, while ATP binding, metal/zinc-ion binding, and DNA binding dominated the molecular-function category (Fig. S3–S4).

For the non-coding RNAs, we also identified 78,048 microRNAs, 1,009 transfer RNAs, 320 ribosomal RNAs and 13,420 small nuclear RNAs in the *T. oliveri* genome (Table S3). Similarly, the *T. siamensis* genome contained 36,297 microRNAs, 1,049 transfer RNAs, 336 ribosomal RNAs, and 11,678 small nuclear RNAs (Table S4).

Identification of repetitive elements

Comparative analysis of repetitive elements in the two *Thyrsostachys* genomes reveals both shared architecture and lineage-specific variation. We grouped repeats into known classes (e.g., LTR, LINE, DNA transposons) and an unclassified category (Other) for sequences lacking clear annotation. Repeats constitute 50.89% of the *T. oliveri* assembly (Table 3) and 48.78% of the *T. siamensis* assembly (Table 4). In *T. oliveri*, unclassified repeats are most abundant (64.55% of all repeats), followed by retrotransposons (24.94%). Within retrotransposons, LTR elements of the Copia and Gypsy superfamilies contribute 13.09% and 9.86% of the genome, respectively. *T. siamensis* exhibits a similar profile: unclassified repeats dominate (67.88%), with Copia and Gypsy elements contributing 13.00% and 8.20% of the genome, respectively. The overall repeat content is approximately two percentage points lower in *T. siamensis*, mainly owing to its smaller Gypsy fraction.

Table 3. Repeat elements in the *T. oliveri* genome assembly.

Types of repeats	Bases (Mb)	% of the assembly	% of total repeats
**DNA transposons:**	47.68	4.81	9.46
**Retrotransposons:**			
LINE	8.47	0.85	1.68
SINE	0.09	0.00	0.00
LTR: *Copia*	65.94	6.66	13.09
LTR: *Gypsy*	49.68	5.02	9.86
LTR: Others	1.56	0.16	0.31
**Simple sequence repeats:**	5.31	0.54	1.05
**Others:**	325.08	32.85	64.55
**Total**	503.81	50.89	

Table 4. Repeat elements in the *T. siamesis* genome assembly.

Types of repeats	Bases (Mb)	% of the assembly	% of total repeats
**DNA transposons:**	45.77	4.01	8.22
**Retrotransposons:**			
LINE	9.09	0.80	1.63

SINE	0.00	0.00	0.00
LTR: *Copia*	72.08	6.32	13.00
LTR: *Gypsy*	45.62	4.00	8.20
LTR: Others	0.31	0.03	0.05
**Simple sequence repeats:**	5.70	0.50	1.02
**Others:**	377.90	33.12	67.88
**Total**	556.47	48.78	

Phylogenetic and comparative genomics analyses

To estimate the relative timing of divergence and WGD events in the Thyrsostachys lineage, we first calculated four-fold degenerate transversion (4DTv) distances for orthologous and paralogous gene pairs (Fig. 2A). The 4DTv distance between *T. oliveri* and *T. siamensis* (0.0164) was lower than that between *T. oliveri* and *D. sinicus* (0.0178), *B. amplexicaulis* (0.0180), *P. edulis* (0.0516), or *O. latifolia* (0.0810), indicating a closer affinity of the two Thyrsostachys species. Analysis of 28,430 paralogous pairs in *T. oliveri* revealed 4DTv peaks at 0.056 and 0.208, and 28,061 paralogous pairs in *T. siamensis* showed peaks at 0.059 and 0.231, consistent with a relatively recent whole-genome duplication in both species.

Next, we reconstructed a maximum-likelihood phylogeny from 605 single-copy orthologues sampled from Thyrsostachys and eight reference grasses (*B. amplexicaulis*, *P. edulis*, *D. latiflorus*, *D. sinicus*, *G. angustifolia*, *R. guianensis*, *B. distachyon*, and *O. sativa*) (Fig. 2B). The two Thyrsostachys species form a clade

sister to the Dendrocalamus pair, with a crown age of 5.39 million years ago (Mya) and a stem divergence from Dendrocalamus at 10.55 Ma

Orthogroup clustering placed 436,476 of 469,186 proteins (93 %) into 37,153 families. A core set of 12,894 families is shared by all 11 species (Fig. 2C), while species-unique families number 319 in *T. oliveri*, enriched for root-development and plasmodesmata genes, and 1,204 in *T. siamensis*, highlighting histone-binding and proteasome pathways (Tables S5–S6).

Finally, gene-copy profiling revealed that hexaploid *D. latiflorus* has undergone the greatest expansion of gene families, followed by *T. siamensis*, with *T. oliveri* showing fewer duplications (Fig. 2D). In *T. oliveri*, 313 families expanded and 637 contracted: in *T. siamensis*, 605 expanded and 354 contracted. GO enrichment of T. oliveri expansions highlights protein-kinase activity, defense response, and monooxygenase activity (Fig. S5), whereas T. siamensis expansions are enriched for ADP-binding, carbohydrate-binding, and DNA-integration functions (Fig. S6).

Homoeolog expression patterns

From RNA-seq of leaf and root (three biological replicates each), we identified 2,197 homoeologous triads in *T. oliveri* and 2,054 triads in *T. siamensis*. Triads in each tissue with a combined expression of A, B, and C homoeolog expression greater than 0.5 TPM were designated “expressed” and classified into six relative-expression categories (Fig. 3; Table S7). Balanced expression was observed in 36.8 % of expressed *T. oliveri* triads and 33.8% of expressed *T. siamensis* triads, whereas

single-homoeolog dominance was the least common (23.4% and 24.6%, respectively). Single-homoeolog suppression affected 39.9% of expressed *T. oliveri* triads and 41.8% of expressed *T. siamensis* triads. Within the dominance categories, B-homoeolog dominance was rare (7.3% in *T. oliveri*; 7.8% in *T. siamensis*), while A- and C-homoeolog dominance showed slightly higher frequencies. Among the suppression categories, B-homoeolog suppression was most frequent in *T. oliveri* (14.5%), whereas C-homoeolog suppression led in *T. siamensis* (14.6%) (Table S7).

Discussion

Bamboo is one of the world's most important non-timber forest resources and a crucial component of forest ecosystems. Despite its significance, only a limited number of bamboo genomes have been sequenced to date, resulting in limited knowledge of bamboo biological mechanisms and hindering progress in understanding genome evolution and the potential for molecular breeding. In this study, we sequenced and assembled chromosome-level genomes of two bamboo species, *T. oliveri* and *T. siamensis*, which resulted in the first reference genome for the *Thyrsostachys* genus. By combining stLFR sequencing technology with the Hi-C technique, we achieved a chromosome-scale assembly for *T. oliveri* and used RagTag scaffolding to assemble the genome of *T. siamensis*. The assembled genome sizes of *T. oliveri* and *T. siamensis* were 990.1 Mb (N50 = 22.18 Mb) and 1.14 Gb (N50 = 19.45 Mb), respectively. The assemblies were anchored into 35 pseudochromosomes, and subgenome partitioning identified three distinct subgenomes, supporting a hexaploid structure (2n = 6x = 70). Compared with previously reported hexaploid bamboo genome assemblies, the genome sizes of *T.*

oliveri and *T. siamensis* are smaller than those of *D. latiflorus* (1,368 Mb, 1C) [22] and *D. brandisii* (1,378 Mb, 1C)[21], but larger than that of *B. amplexicaulis* (848 Mb) [9]. Based on the BUSCO assessment, the completeness of the gene space in the *T. oliveri* and *T. siamensis* genomes was estimated at 97.4% and 95.2%, respectively, indicating that the current assemblies cover most of their genomes. The assembly size of *T. oliveri* is slightly smaller than the estimated genome size based on both k-mer analysis and flow cytometry (1.157 Gb). This slight difference likely reflects unassembled repetitive portions of the genome. In contrast, the assembly size of *T. siamensis* is close to the estimates from both methods (1.172 Gb). These results further indicate that the assemblies are largely representative of the complete genomes and demonstrate high quality.

The genome annotations of *T. oliveri* and *T. siamensis* contained 51,191 and 67,483 predicted gene models, respectively, with *T. oliveri* having fewer predicted gene models than *T. siamensis*. Compared with other bamboo genomes, the number of annotated genes in the haploid assembly of *T. oliveri* is similar to that reported for *P. edulis* (51,074) [13] and higher than that of *B. amplexicaulis* (47,056) [9]. In contrast, the 67,483 genes in the haploid *T. siamensis* assembly correspond to roughly half the total counts observed in diploid-level assemblies: 135,231 genes for *D. latiflorus* [22] and 126,817 genes for *D. brandisii* [21], both of which were assembled at 2n = 70. The proportions of repetitive sequences identified in our genome assemblies were 50.89% for *T. oliveri* and 48.78% for *T. siamensis*. The composition of repeat types was highly similar between the two species, which is unsurprising given their close evolutionary relationship. These findings reveal both the conserved and dynamic aspects of repetitive element evolution in *Thyrsostachys*. The observed similarities suggest a shared ancestral repertoire of repetitive elements. In contrast, the

differences in abundance and composition indicate that these elements have undergone independent evolutionary changes in the two species, likely in response to differing evolutionary pressures influencing their genomes. The content of repetitive elements in our genome assemblies is slightly lower than that reported for moso bamboo [13, 23], *D. latiflorus* [22], and *D. brandisii* [21]. LTR elements, which represent over 60% of repetitive sequences, are the predominant retrotransposon classes in our bamboo assemblies. Consistent with previous studies, LTR retroelements are also the most common elements in bamboo genomes [13, 21, 22].

A phylogenetic analysis based on sequence information from single-copy orthologous genes revealed that *T. oliveri* and *T. siamensis* are the most closely related, having diverged approximately 5.39 Mya. The analyses of accumulated nucleotide divergence at 4DTv values indicated that the genomes of the hexaploid bamboos *T. oliveri* and *T. siamensis* have undergone a recent whole-genome duplication event, which likely occurred in their common ancestor prior to the speciation event. Identifying genes that differ between bamboo and closely related species is important for uncovering the phenotypic specificity of bamboo and mechanisms underlying the adaptive divergence of related species. Gene family analysis revealed that 12,894 gene families were shared among 11 species, while 319 and 1,204 species-specific gene families were identified in *T. oliveri* and *T. siamensis*, respectively. These gene families, identified as unique to each species, may represent characteristic genomic features and contribute to species-specific traits. Some of these genes are potentially involved in important biological processes, for example, the regulation of root development and carbon metabolism in *T. oliveri*, and protein degradation in *T. siamensis*. The expansion and contraction of gene families play critical roles in driving phenotypic diversification and enhancing specialized traits in

plants [75]. In our analysis, *T. oliveri* was found to have fewer expanded gene families and more contracted gene families compared to *T. siamensis*. In contrast, *T. siamensis* exhibited a greater number of expanded gene families and fewer contractions. Given that *T. siamensis* also had a higher overall gene count, it is likely that the expanded families in this species contain more gained genes than were lost through contraction, potentially contributing to its distinct genomic and phenotypic features. By examining the expanded gene families in *T. oliveri*, we found enrichment in genes associated with protein kinase activity and monooxygenase activity. In *T. siamensis*, expanded gene families were enriched in functions related to ADP binding and protein dimerization activity. These species-specific expansions and contractions of gene families provide insights into the phenotypic characteristics and distinct evolutionary pressures that have shaped the genomes of these two *Thyrsostachys* species.

Allopolyploidy is a defining feature of many bamboo species. Previous studies have shown that duplicated gene pairs may display homoeolog expression bias in several allopolyploid species [76-80], in which bias refers to the preferential expression of one homoeolog relative to the other [81]. In our hexaploid *Thyrsostachys* species, single-homoeolog suppression was the most frequent expression category, affecting 39.9% of expressed triads in *T. oliveri* and 41.8% in *T. siamensis*. This pattern has also been observed in other woody bamboos, in which single-homoeolog suppression tends to be more common [71]. Balanced expression accounted for 36.8% of triads in *T. oliveri* and 33.8% in *T. siamensis*, consistent with coordinated subgenome regulation observed in hexaploid bamboo species [71]. Single-homoeolog dominance was relatively uncommon (<25%). Among the dominant triads, the B-subgenome was the least frequently dominant, while A- and C-subgenomes showed comparable but

slightly higher levels of dominance. These findings are consistent with previous reports in *M. baccifera*, *B. amplexicaulis* and *D. sinicus* bamboo species [71]. Notably, suppression biases differed between species: B-subgenome suppression predominated in *T. oliveri*, whereas C-subgenome suppression was more frequent in *T. siamensis*, suggesting lineage-specific regulatory divergence. Together, these findings indicate that, while balanced expression remains substantial, single-homoeolog suppression is a major driver of subgenome-specific expression and may underlie functional differentiation in these two *Thyrsostachys* species.

Data Availability

Both *T. oliveri* and *T. siamensis* genome assemblies have been deposited at NCBI under accession numbers JAWCWW000000000 and JBEFOJ000000000, respectively. The raw stLFR reads for *T. oliveri* and *T. siamensis* are available in the NCBI SRA database under accession numbers SRR32915794 and SRR32915889, respectively. The transcriptome data for *T. oliveri* were submitted under SRR33015685 and SRR33015684. The transcriptome data for *T. siamensis* were submitted under SRR33015804 and SRR33015803.

Abbreviations

Hi-C: chromosome conformation capture; stLFR: single-tube long-fragment read ;4DTv: four-fold degenerate transversion; BLASTP: Basic Local Alignment Search Tool for protein; bp: base pair; BUSCO: Benchmarking Universal Single-Copy Orthologues; EVM: EvidenceModeler; GO: Gene Ontology; kb: kilobase; Gb:

gigabase; KEGG: Kyoto Encyclopedia of Genes and Genomes; LTR: long terminal repeat; Mb: megabase; Mya: million years ago;

Additional files

Supplementary Table S1. Summary of sequencing data from stLFR and Hi-C platforms.

Supplementary Table S2. Functional annotations of *T. oliveri* and *T. siamensis* protein-coding genes.

Supplementary Table S3. Noncoding RNA in the *T. oliveri* genome.

Supplementary Table S4. Noncoding RNA in the *T. siamensis* genome.

Supplementary Table S5. GO term enrichment analyses for gene families specific to *T. oliveri.*

Supplementary Table S6. GO term enrichment analyses for gene families specific to *T. siamensis.*

Supplementary Table S7. Percentage of syntenic triads assigned to the six homoeolog expression bias categories.

Supplementary Fig. S1 Flow-cytometric estimation of nuclear DNA content in *Thyrsostachys* species. Nuclear DNA content was measured by propidium-iodide (PI) staining of nuclei isolated from fresh leaf tissue of (A) *T. siamensis* and (B) *T. oliveri.*

Supplementary Fig. S2 Genome-survey k-mer distributions for hexaploid *Thyrsostachys*.

Supplementary Fig. S3 GO annotation statistics for *T. oliveri*.

Supplementary Fig. S4 Annotation statistics for T. *siamensis*.

Supplementary Fig. S5A GO term enrichment analyses for expanded gene families in *T. oliveri.* (A) Enriched biological processes among expanded gene families in *T. oliveri.* (B) Enriched cellular components among expanded gene families in *T. oliveri.* (C) Enriched molecular functions among expanded gene families in *T. oliveri.*

Supplementary Fig. S5B GO term enrichment analyses for contracted gene families in *T. oliveri*. (A) Enriched biological processes among contracted gene families in *T. oliveri*. (B) Enriched molecular functions among contracted gene families in *T. oliveri*.

Supplementary Fig. S6A GO term enrichment analyses for expanded gene families in *T. siamensis*. (A) Enriched biological processes among expanded gene families in *T. siamensis*. (B) Enriched cellular components among expanded gene families in *T. siamensis*. (C) Enriched molecular functions among expanded gene families in T. *siamensis*.

Supplementary Fig. S6B GO term enrichment analyses for contracted gene families in *T. siamensis*. (A) Enriched biological processes among contracted gene families in *T. siamensis*. (B) Enriched cellular components among contracted gene families in *T. siamensis*. (C) Enriched molecular functions among expanded gene families in *T. siamensis*.

Supplementary Fig. S7 Genome-wide Hi-C contact map of hexaploid *T. oliveri*.

Funding

This work was supported by the National Science and Technology Development Agency (NSTDA), grant number P2251271.

Competing Interests

The authors declare that they have no competing interests.

Author Contributions

Research study was designed by C.N., S.K., W.P. and S.T. Sample collection and laboratory work (DNA/RNA extraction, sequencing library preparation, flow cytometry) were performed by S.K., S.U-t., D.S., P.W., and S.S. Bioinformatics analyses were performed by C.N., P.P., and C.S. The manuscript was written and revised by C.N., S.K. and W.P. All authors read and approved the final manuscript.

Reference

1. Zhang H, Zhuang S, Sun B, Ji H, Li C and Zhou S. Estimation of biomass and carbon storage of moso bamboo (Phyllostachys pubescens Mazel ex Houz.) in southern China using a diameter–age bivariate distribution model. Forestry: An International Journal of Forest Research. 2014;87 5:674-82. doi:10.1093/forestry/cpu028.
2. Jember AA, Taye MA, Gebeyehu G, Mulu G, Long TT, Jayaraman D and Abebe S. Carbon stock potential of highland bamboo plantations in northwestern Ethiopia. Carbon Balance and Management. 2023;18 1:3. doi:10.1186/s13021-023-00224-2.
3. Basak M, Dutta S, Biswas S, Chakraborty S, Sarkar A, Rahaman T, et al. Genomic insights into growth and development of bamboos: what have we learnt and what more to discover? Trees. 2021;35 6:1771-91. doi:10.1007/s00468-021-02197-6.
4. Du H, Mao F, Li X, Zhou G, Xu X, Han N, et al. Mapping Global Bamboo Forest Distribution Using Multisource Remote Sensing Data. IEEE Journal of Selected Topics in Applied Earth Observations and Remote Sensing. 2018;11 5:1458-71. doi:10.1109/JSTARS.2018.2800127.
5. Food and Agriculture Organization of the United N. Putting bamboo on the map. 2021.

6. Soreng RJ, Peterson PM, Romaschenko K, Davidse G, Teisher JK, Clark LG, et al. A worldwide phylogenetic classification of the Poaceae (Gramineae) II: An update and a comparison of two 2015 classifications. Journal of Systematics and Evolution. 2017;55 4:259-90. doi:https://doi.org/10.1111/jse.12262.

7. Akinlabi ET, Anane-Fenin K and Akwada DR. Bamboo Taxonomy and Distribution Across the Globe. In: Akinlabi ET, Anane-Fenin K and Akwada DR, editors. Bamboo: The Multipurpose Plant. Cham: Springer International Publishing; 2017. p. 1-37.

8. Zhou M, Xu C, Shen L, Xiang W and Tang D. Evolution of genome sizes in Chinese Bambusoideae (Poaceae) in relation to karyotype. Trees. 2017;31 1:41-8. doi:10.1007/s00468-016-1453-y.

9. Guo Z-H, Ma P-F, Yang G-Q, Hu J-Y, Liu Y-L, Xia E-H, et al. Genome Sequences Provide Insights into the Reticulate Origin and Unique Traits of Woody Bamboos. Molecular Plant. 2019;12 10:1353-65. doi:https://doi.org/10.1016/j.molp.2019.05.009.

10. Clark L, Londoño X and Ruiz-Sanchez E. Bamboo taxonomy and habitat. Bamboo: The plant and its uses. 2015:1-30.

11. Group BP. An updated tribal and subtribal classification of the bamboos (Poaceae: Bambusoideae). The Journal of the American Bamboo Society. 2012:1.

12. Zhao H, Zhao S, Bamboo INf, Rattan, Fei B, Liu H, et al. Announcing the Genome Atlas of Bamboo and Rattan (GABR) project: promoting research in evolution and in economically and ecologically beneficial plants. GigaScience. 2017;6 7:gix046.

13. Zhao H, Gao Z, Wang L, Wang J, Wang S, Fei B, et al. Chromosome-level reference genome and alternative splicing atlas of moso bamboo (Phyllostachys edulis). GigaScience. 2018;7 10 doi:10.1093/gigascience/giy115.

14. Schröder S. Thyrsostachys siamensis – Monastery Bamboo. 2020.

15. Sae-Long W, Chompoorat T, Limkatanyu S, Hansapinyo C, Buakla A, Sukontasukkul P, et al. Investigation on the tensile strength of Dendrocalamus sericeus, Phyllostachys makinoi, and Thyrsostachys oliveri bamboo:

Experiment and simulations. Case Studies in Construction Materials. 2024;20:e03205. doi:https://doi.org/10.1016/j.cscm.2024.e03205.

16. Thailand Environment I, Marueng V, Tanawat T, Pakdeelun W and Klung-ngoen W. *Bamboo Value Chain Analysis in Thailand*. 2021-09 2021. Bangkok: Thailand Environment Institute.
17. Banik RL. Thyrsostachys Gamble. Silviculture of South Asian Priority Bamboos. Singapore: Springer Singapore; 2016. p. 261-76.
18. Thyrsostachys oliveri. CABI Compendium. 2022; doi:10.1079/cabicompendium.53799.
19. Chaowana K, Wisadsatorn S and Chaowana P. Bamboo as a Sustainable Building Material—Culm Characteristics and Properties. Sustainability. 2021;13 13:7376.
20. Obsuwan K, Duangmanee A and Thepsithar C. In vitro propagation of a useful tropical bamboo, Thyrsostachys siamensis Gamble, through shoot-derived callus. Horticulture, Environment, and Biotechnology. 2019;60 2:261-7. doi:10.1007/s13580-018-00119-z.
21. Jiang J, Zhang Z, Bai Y, Wang X, Dou Y, Geng R, et al. Chromosomal-level genome and metabolome analyses of highly heterozygous allohexaploid Dendrocalamus brandisii elucidate shoot quality and developmental characteristics. J Integr Plant Biol. 2024;66 6:1087-105. doi:10.1111/jipb.13592.
22. Zheng Y, Yang D, Rong J, Liguang C, Zhu Q, He T, et al. Allele-aware chromosome-scale assembly of the allopolyploid genome of hexaploid Ma bamboo (Dendrocalamus latiflorus Munro). Journal of Integrative Plant Biology. 2022;64 doi:10.1111/jipb.13217.
23. Peng Z, Lu Y, Li L, Zhao Q, Feng Q, Gao Z, et al. The draft genome of the fast-growing non-timber forest species moso bamboo (Phyllostachys heterocycla). Nature Genetics. 2013;45 4:456-61. doi:10.1038/ng.2569.
24. Ma P-F, Liu Y-L, Guo C, Jin G, Guo Z-H, Mao L, et al. Genome assemblies of 11 bamboo species highlight diversification induced by dynamic subgenome dominance. Nature Genetics. 2024;56 4:710-20. doi:10.1038/s41588-024-01683-0.

25. Wang O, Chin R, Cheng X, Wu MKY, Mao Q, Tang J, et al. Efficient and unique cobarcoding of second-generation sequencing reads from long DNA molecules enabling cost-effective and accurate sequencing, haplotyping, and de novo assembly. Genome research. 2019;29 5:798-808.

26. Pootakham W, Sonthirod C, Naktang C, Yundaeng C, Yoocha T, Kongkachana W, et al. Genome assemblies of Vigna reflexo-pilosa (créole bean) and its progenitors, Vigna hirtella and Vigna trinervia, revealed homoeolog expression bias and expression-level dominance in the allotetraploid. GigaScience. 2023;12:giad050. doi:10.1093/gigascience/giad050.

27. Xie T, Zheng J-F, Liu S, Peng C, Zhou Y-M, Yang Q-Y and Zhang H-Y. De Novo Plant Genome Assembly Based on Chromatin Interactions: A Case Study of Arabidopsis thaliana. Molecular Plant. 2015;8 3:489-92. doi:https://doi.org/10.1016/j.molp.2014.12.015.

28. Putnam NH, O'Connell BL, Stites JC, Rice BJ, Blanchette M, Calef R, et al. Chromosome-scale shotgun assembly using an in vitro method for long-range linkage. Genome Res. 2016;26 3:342-50. doi:10.1101/gr.193474.115.

29. Li H and Durbin R. Fast and accurate short read alignment with Burrows–Wheeler transform. Bioinformatics. 2009;25 14:1754-60. doi:10.1093/bioinformatics/btp324.

30. Servant N, Varoquaux N, Lajoie BR, Viara E, Chen C-J, Vert J-P, et al. HiC-Pro: an optimized and flexible pipeline for Hi-C data processing. Genome Biology. 2015;16 1:259. doi:10.1186/s13059-015-0831-x.

31. Alonge M, Soyk S, Ramakrishnan S, Wang X, Goodwin S, Sedlazeck FJ, et al. RaGOO: fast and accurate reference-guided scaffolding of draft genomes. Genome Biology. 2019;20 1:224. doi:10.1186/s13059-019-1829-6.

32. Vurture GW, Sedlazeck FJ, Nattestad M, Underwood CJ, Fang H, Gurtowski J and Schatz MC. GenomeScope: fast reference-free genome profiling from short reads. Bioinformatics. 2017;33 14:2202-4. doi:10.1093/bioinformatics/btx153.

33. DoleŽEl J and BartoŠ JAN. Plant DNA Flow Cytometry and Estimation of Nuclear Genome Size. Annals of Botany. 2005;95 1:99-110. doi:10.1093/aob/mci005.

34. Galbraith Dw Fau - Harkins KR, Harkins Kr Fau - Maddox JM, Maddox Jm Fau - Ayres NM, Ayres Nm Fau - Sharma DP, Sharma Dp Fau - Firoozabady E and Firoozabady E. Rapid flow cytometric analysis of the cell cycle in intact plant tissues. 0036-8075 (Print).
35. Kim D, Paggi JM, Park C, Bennett C and Salzberg SL. Graph-based genome alignment and genotyping with HISAT2 and HISAT-genotype. Nature Biotechnology. 2019;37 8:907-15. doi:10.1038/s41587-019-0201-4.
36. Manni M, Berkeley MR, Seppey M, Simão FA and Zdobnov EM. BUSCO Update: Novel and Streamlined Workflows along with Broader and Deeper Phylogenetic Coverage for Scoring of Eukaryotic, Prokaryotic, and Viral Genomes. Molecular Biology and Evolution. 2021;38 10:4647-54. doi:10.1093/molbev/msab199.
37. Kriventseva EV, Tegenfeldt F, Petty TJ, Waterhouse RM, Simão FA, Pozdnyakov IA, et al. OrthoDB v8: update of the hierarchical catalog of orthologs and the underlying free software. Nucleic Acids Research. 2015;43 D1:D250-D6. doi:10.1093/nar/gku1220.
38. Flynn JM, Hubley R, Goubert C, Rosen J, Clark AG, Feschotte C and Smit AF. RepeatModeler2 for automated genomic discovery of transposable element families. Proceedings of the National Academy of Sciences. 2020;117 17:9451-7. doi:10.1073/pnas.1921046117.
39. Price AL, Jones NC and Pevzner PA. De novo identification of repeat families in large genomes. Bioinformatics. 2005;21 suppl_1:i351-i8. doi:10.1093/bioinformatics/bti1018.
40. Bao Z and Eddy SR. Automated de novo identification of repeat sequence families in sequenced genomes. 1088-9051 (Print).
41. Tempel S. Using and Understanding RepeatMasker. In: Bigot Y, editor. Mobile Genetic Elements: Protocols and Genomic Applications. Totowa, NJ: Humana Press; 2012. p. 29-51.
42. Jurka J, Kapitonov VV, Pavlicek A, Klonowski P, Kohany O and Walichiewicz J. Repbase Update, a database of eukaryotic repetitive elements. Cytogenetic and Genome Research. 2005;110 1-4:462-7. doi:10.1159/000084979.
43. Haas BJ, Salzberg SL, Zhu W, Pertea M, Allen JE, Orvis J, et al. Automated eukaryotic gene structure annotation using EVidenceModeler and the

Program to Assemble Spliced Alignments. Genome Biology. 2008;9 1:R7. doi:10.1186/gb-2008-9-1-r7.

44. Holt C and Yandell M. MAKER2: an annotation pipeline and genome-database management tool for second-generation genome projects. BMC Bioinformatics. 2011;12 1:491. doi:10.1186/1471-2105-12-491.

45. Haas BJ, Papanicolaou A, Yassour M, Grabherr M, Blood PD, Bowden J, et al. De novo transcript sequence reconstruction from RNA-seq using the Trinity platform for reference generation and analysis. Nature Protocols. 2013;8 8:1494-512. doi:10.1038/nprot.2013.084.

46. Li W and Godzik A. Cd-hit: a fast program for clustering and comparing large sets of protein or nucleotide sequences. Bioinformatics. 2006;22 13:1658-9. doi:10.1093/bioinformatics/btl158.

47. Fu L, Niu B Fau - Zhu Z, Zhu Z Fau - Wu S, Wu S Fau - Li W and Li W. CD-HIT: accelerated for clustering the next-generation sequencing data. 1367-4811 (Electronic).

48. Haas BJ, Delcher AL, Mount SM, Wortman JR, Smith Jr RK, Hannick LI, et al. Improving the Arabidopsis genome annotation using maximal transcript alignment assemblies. Nucleic Acids Research. 2003;31 19:5654-66. doi:10.1093/nar/gkg770.

49. Wu TD and Watanabe CK. GMAP: a genomic mapping and alignment program for mRNA and EST sequences. Bioinformatics. 2005;21 9:1859-75. doi:10.1093/bioinformatics/bti310.

50. Huang X, Adams MD, Zhou H and Kerlavage AR. A Tool for Analyzing and Annotating Genomic Sequences. Genomics. 1997;46 1:37-45. doi:https://doi.org/10.1006/geno.1997.4984.

51. Stanke M, Steinkamp R, Waack S and Morgenstern B. AUGUSTUS: a web server for gene finding in eukaryotes. Nucleic Acids Research. 2004;32 suppl_2:W309-W12. doi:10.1093/nar/gkh379.

52. Korf I. Gene finding in novel genomes. BMC Bioinformatics. 2004;5 1:59. doi:10.1186/1471-2105-5-59.

53. Gish W and States DJ. Identification of protein coding regions by database similarity search. Nature Genetics. 1993;3 3:266-72. doi:10.1038/ng0393-266.

54. Chan Patricia P, Lin Brian Y, Mak Allysia J and Lowe Todd M. tRNAscan-SE 2.0: improved detection and functional classification of transfer RNA genes. Nucleic Acids Research. 2021;49 16:9077-96. doi:10.1093/nar/gkab688.

55. Griffiths-Jones S, Moxon S, Marshall M, Khanna A, Eddy SR and Bateman A. Rfam: annotating non-coding RNAs in complete genomes. Nucleic Acids Research. 2005;33 suppl_1:D121-D4. doi:10.1093/nar/gki081.

56. Nawrocki EP and Eddy SR. Infernal 1.1: 100-fold faster RNA homology searches. Bioinformatics. 2013;29 22:2933-5. doi:10.1093/bioinformatics/btt509.

57. Emms DM and Kelly S. OrthoFinder: phylogenetic orthology inference for comparative genomics. Genome Biology. 2019;20 1:238. doi:10.1186/s13059-019-1832-y.

58. Edgar RC. MUSCLE: a multiple sequence alignment method with reduced time and space complexity. BMC Bioinformatics. 2004;5 1:113. doi:10.1186/1471-2105-5-113.

59. Capella-Gutiérrez S, Silla-Martínez JM and Gabaldón T. trimAl: a tool for automated alignment trimming in large-scale phylogenetic analyses. Bioinformatics. 2009;25 15:1972-3. doi:10.1093/bioinformatics/btp348.

60. Darriba D, Posada D, Kozlov AM, Stamatakis A, Morel B and Flouri T. ModelTest-NG: A New and Scalable Tool for the Selection of DNA and Protein Evolutionary Models. Molecular Biology and Evolution. 2020;37 1:291-4. doi:10.1093/molbev/msz189.

61. Kozlov AM, Darriba D, Flouri T, Morel B and Stamatakis A. RAxML-NG: a fast, scalable and user-friendly tool for maximum likelihood phylogenetic inference. Bioinformatics. 2019;35 21:4453-5. doi:10.1093/bioinformatics/btz305.

62. Bouckaert R, Vaughan TG, Barido-Sottani J, Duchêne S, Fourment M, Gavryushkina A, et al. BEAST 2.5: An advanced software platform for Bayesian evolutionary analysis. PLOS Computational Biology. 2019;15 4:e1006650. doi:10.1371/journal.pcbi.1006650.

63. Strömberg CAE. Decoupled taxonomic radiation and ecological expansion of open-habitat grasses in the Cenozoic of North America. Proceedings of the National Academy of Sciences. 2005;102 34:11980-4. doi:10.1073/pnas.0505700102.

64. Prasad V, Strömberg CAE, Leaché AD, Samant B, Patnaik R, Tang L, et al. Late Cretaceous origin of the rice tribe provides evidence for early diversification in Poaceae. Nature Communications. 2011;2 1:480. doi:10.1038/ncomms1482.
65. Zhang X-Z, Zeng C-X, Ma P-F, Haevermans T, Zhang Y-X, Zhang L-N, et al. Multi-locus plastid phylogenetic biogeography supports the Asian hypothesis of the temperate woody bamboos (Poaceae: Bambusoideae). Molecular Phylogenetics and Evolution. 2016;96:118-29. doi:https://doi.org/10.1016/j.ympev.2015.11.025.
66. Rambaut A, Drummond AJ, Xie D, Baele G and Suchard MA. Posterior Summarization in Bayesian Phylogenetics Using Tracer 1.7. Systematic Biology. 2018;67 5:901-4. doi:10.1093/sysbio/syy032.
67. Mendes FK, Vanderpool D, Fulton B and Hahn MW. CAFE 5 models variation in evolutionary rates among gene families. Bioinformatics. 2021;36 22-23:5516-8. doi:10.1093/bioinformatics/btaa1022.
68. Wang Y, Tang H, DeBarry JD, Tan X, Li J, Wang X, et al. MCScanX: a toolkit for detection and evolutionary analysis of gene synteny and collinearity. Nucleic Acids Research. 2012;40 7:e49-e. doi:10.1093/nar/gkr1293.
69. Krzywinski M, Schein J Fau - Birol I, Birol I Fau - Connors J, Connors J Fau - Gascoyne R, Gascoyne R Fau - Horsman D, Horsman D Fau - Jones SJ, et al. Circos: an information aesthetic for comparative genomics. 1549-5469 (Electronic).
70. He W, Yang J, Jing Y, Xu L, Yu K and Fang X. NGenomeSyn: an easy-to-use and flexible tool for publication-ready visualization of syntenic relationships across multiple genomes. Bioinformatics. 2023;39 3:btad121. doi:10.1093/bioinformatics/btad121.
71. Ma PF, Liu YL, Guo C, Jin G, Guo ZH, Mao L, et al. Genome assemblies of 11 bamboo species highlight diversification induced by dynamic subgenome dominance. Nat Genet. 2024;56 4:710-20. doi:10.1038/s41588-024-01683-0.
72. tanghaibao: jcvi: JCVI utility libraries (v0.5.7). (2017).
73. Hamilton NE and Ferry M. ggtern: Ternary Diagrams Using ggplot2. Journal of Statistical Software, Code Snippets. 2018;87 3:1 - 17. doi:10.18637/jss.v087.c03.

74. Ramírez-González RH, Borrill P, Lang D, Harrington SA, Brinton J, Venturini L, et al. The transcriptional landscape of polyploid wheat. Science. 2018;361 6403:eaar6089. doi:10.1126/science.aar6089.
75. Kang M, Wu H, Yang Q, Huang L, Hu Q, Ma T, et al. A chromosome-scale genome assembly of Isatis indigotica, an important medicinal plant used in traditional Chinese medicine. Horticulture Research. 2020;7 1:18. doi:10.1038/s41438-020-0240-5.
76. Li A, Liu D, Wu J, Zhao X, Hao M, Geng S, et al. mRNA and small RNA transcriptomes reveal insights into dynamic homoeolog regulation of allopolyploid heterosis in nascent hexaploid wheat. The Plant Cell. 2014;26 5:1878-900.
77. Akhunova AR, Matniyazov RT, Liang H and Akhunov ED. Homoeolog-specific transcriptional bias in allopolyploid wheat. BMC genomics. 2010;11:1-16.
78. Yoo M, Szadkowski E and Wendel J. Homoeolog expression bias and expression level dominance in allopolyploid cotton. Heredity. 2013;110 2:171-80.
79. Wu J, Lin L, Xu M, Chen P, Liu D, Sun Q, et al. Homoeolog expression bias and expression level dominance in resynthesized allopolyploid Brassica napus. BMC Genomics. 2018;19 1:586. doi:10.1186/s12864-018-4966-5.
80. Combes MC, Cenci A, Baraille H, Bertrand B and Lashermes P. Homeologous gene expression in response to growing temperature in a recent Allopolyploid (Coffea arabica L.). J Hered. 2012;103 1:36-46. doi:10.1093/jhered/esr120.
81. Grover CE, Gallagher JP, Szadkowski EP, Yoo MJ, Flagel LE and Wendel JF. Homoeolog expression bias and expression level dominance in allopolyploids. New Phytologist. 2012;196 4:966-71. doi:https://doi.org/10.1111/j.1469-8137.2012.04365.x.

Figure titles and legends

Figure 1 Genomic landscape and comparative collinearity of hexaploid *Thyrsostachys* and *Oryza sativa*. (A) Circos plot of the hexaploid *Thyrsostachys oliveri* assembly. The outermost ring shows chromosome ideograms for subgenome A (purple; 12 chromosomes), subgenome B (yellow; 12 chromosomes), and subgenome C (orange; 11 chromosomes). From the outermost data track inward:

1. Repeat content—proportion of bases covered by repetitive sequences in 500-kb windows.
2. Gene density—number of genes per 500-kb windows
3. GC content—percentage of G + C bases per 500-kb window.

(B) Circos plot of the *T. siamensis* assembly, formatted identically to panel A.

(C) Global collinearity between the 12 chromosomes of *O. sativa* and the A, B and C subgenomes of *T. oliveri* (left) and *T. siamensis* (right).

Figure 2 Evolutionary analysis of the hexaploid *Thyrsostachys* genome. (A) Density plot showing pairwise four-fold synonymous (degenerative) third-codon transversion (4DTv) values between the indicated pairs of genomes. (B) Phylogenetic tree of 11 plant species, with the predicted divergence time shown as each branch. Adjacent pie charts show numbers of gene family expansion (shown in light green) and contraction (shown in dark green) among 11 species. The size and color of the circles represent the proportion of genes. (C) Flower-petal plot showing orthologous gene-family sharing among the 11 species (center circle) and species-specific gene families (individual petals). (D) Stacked bar chart of gene-family copy-number distributions across the 11 species. Bars are partitioned into categories (0, 1, 2, 3, 4, >4 copies); colors correspond to each copy-number class (legend at right).

Figure 3 Homoeolog expression patterns in hexaploid *Thyrsostachys* genome. Ternary plots of relative expression contributions of the A, B and C subgenome homoeologs in (A) *T. oliveri* and (B) *T. siamensis*. Each point represents one expressed gene triad (sum TPM > 0.5) in either leaf (×) or root (○) tissue; its position reflects the proportion of total expression contributed by each homoeolog.

- Vertices (red, blue or green) indicate single-homoeolog dominance (A-, B- or C-dominant).
- Edges (orange, light-blue or light-green) indicate single-homoeolog suppression (A-, B- or C-suppressed).
- Center (grey) indicates balanced expression among all three homoeologs.

Percentages in the legend denote the fraction of expressed triads in each category.

Supplementary Figure titles and legends

Supplementary Table S1. Summary of sequencing data from stLFR and Hi-C platforms.

Supplementary Table S2. Functional annotations of *T. oliveri* and *T. siamensis* protein-coding genes.

Supplementary Table S3. Noncoding RNA in the *T. oliveri* genome.

Supplementary Table S4. Noncoding RNA in the *T. siamensis* genome.

Supplementary Table S5. GO term enrichment analyses for gene families specific to *T. oliveri.*

Supplementary Table S6. GO term enrichment analyses for gene families specific to *T. siamensis.*

Supplementary Table S7. Percentage of syntenic triads assigned to the six homoeolog expression bias categories.

Supplementary Fig. S1 Flow-cytometric estimation of nuclear DNA content in *Thyrsostachys* species. Nuclear DNA content was measured by propidium-iodide (PI) staining of nuclei isolated from fresh leaf tissue of (A) *T. siamensis* and (B) *T. oliveri.*

Supplementary Fig. S2 Genome-survey k-mer distributions for hexaploid *Thyrsostachys*.

Supplementary Fig. S3 GO annotation statistics for *T. oliveri*.

Supplementary Fig. S4 Annotation statistics for T*. siamensis*.

Supplementary Fig. S5A GO term enrichment analyses for expanded gene families in *T. oliveri.* (A) Enriched biological processes among expanded gene families in *T. oliveri.* (B) Enriched cellular components among expanded gene families in *T. oliveri.* (C) Enriched molecular functions among expanded gene families in *T. oliveri.*

Supplementary Fig. S5B GO term enrichment analyses for contracted gene families in *T. oliveri*. (A) Enriched biological processes among contracted gene families in *T. oliveri*. (B) Enriched molecular functions among contracted gene families in *T. oliveri*.

Supplementary Fig. S6A GO term enrichment analyses for expanded gene families in *T. siamensis*. (A) Enriched biological processes among expanded gene families in *T. siamensis*. (B) Enriched cellular components among expanded gene families in *T.*

siamensis. (C) Enriched molecular functions among expanded gene families in T. *siamensis*.

Supplementary Fig. S6B GO term enrichment analyses for contracted gene families in *T. siamensis*. (A) Enriched biological processes among contracted gene families in *T. siamensis*. (B) Enriched cellular components among contracted gene families in *T. siamensis*. (C) Enriched molecular functions among expanded gene families in *T. siamensis*.

Supplementary Fig. S7 Genome-wide Hi-C contact map of hexaploid *T. oliveri*.

Click here to access/download;Figure;Fig.1.png

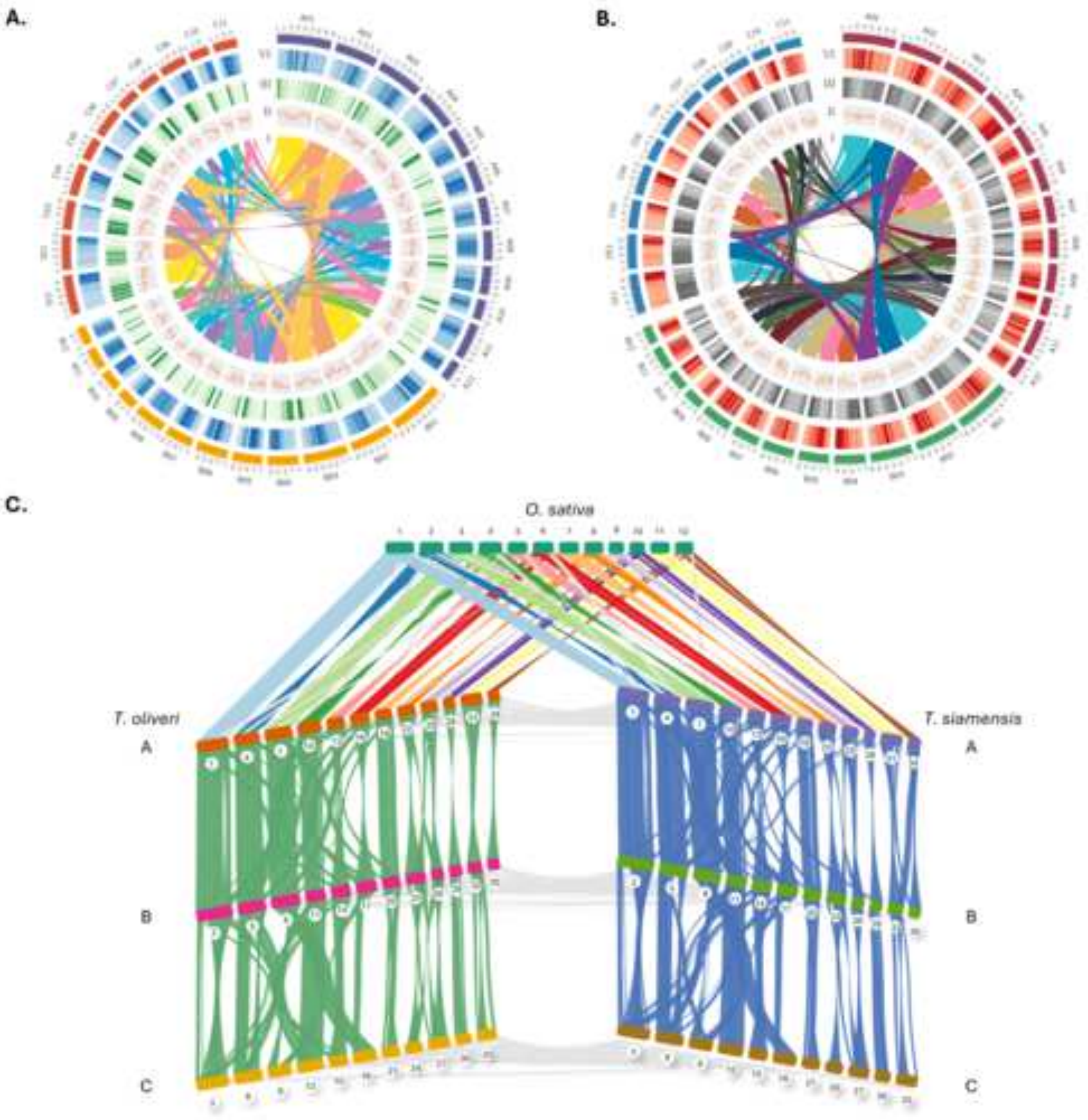

Figure Click here to access/download;Figure;Fig.2.png

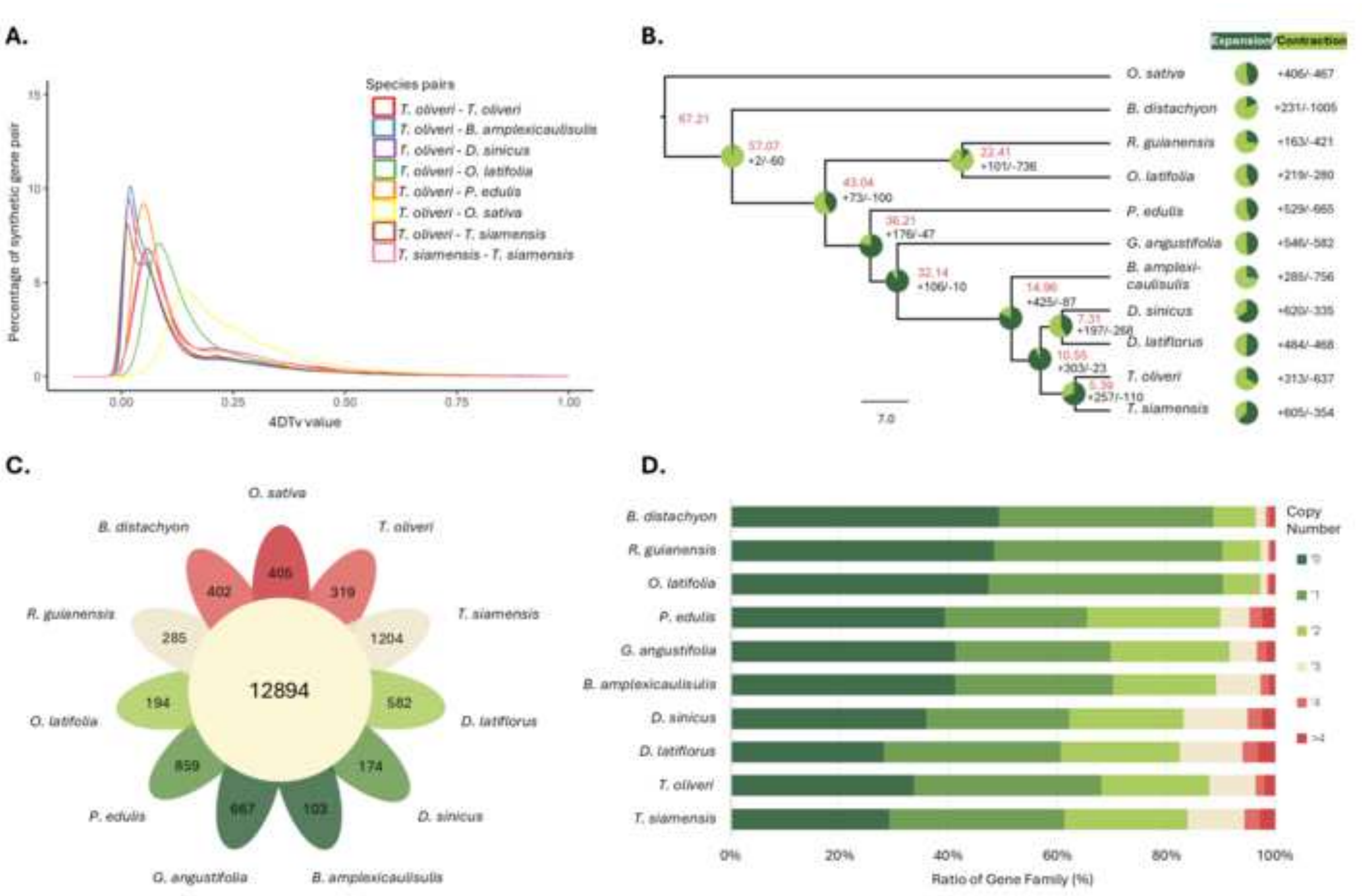

Figure

Click here to access/download;Figure;Fig.3.png

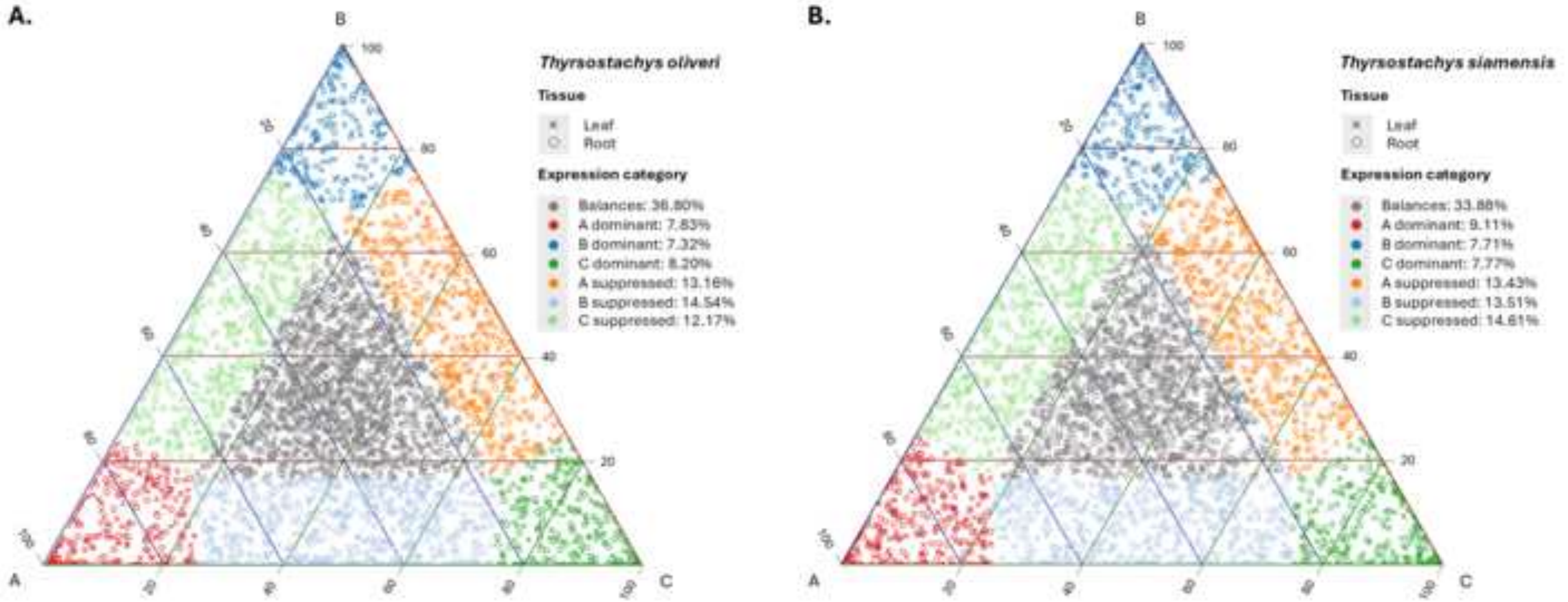

Click here to access/download
Supplementary Material
Supplementary Table.docx

Click here to access/download
Supplementary Material
Supplementary Fig. S1.PNG

Click here to access/download
Supplementary Material
Supplementary Fig. S2.png

Click here to access/download
Supplementary Material
Supplementary Fig. S3.PNG

Click here to access/download
Supplementary Material
Supplementary Fig. S4.png

Click here to access/download
Supplementary Material
Supplementary Fig. S5A.PNG

Click here to access/download
Supplementary Material
Supplementary Fig. S5B.PNG

Click here to access/download
Supplementary Material
Supplementary Fig. S6A.PNG

Click here to access/download
Supplementary Material
Supplementary Fig. S6B.PNG

Click here to access/download
Supplementary Material
Supplementary Fig. S7.jpg
