# Supplementary material for: Chromosome-level assemblies of two hexaploid bamboos, Thyrsostachys oliveri and Thyrsostachys siamensis, provide a foundation for functional and comparative genomics studies
Source: Gigascience. 2025 Nov 17;14:giaf142. doi: 10.1093/gigascience/giaf142 (PMC12699669; doi:10.1093/gigascience/giaf142)
Supplement: giaf142_GIGA-D-25-00232_Revision_1 [file giaf142_giga-d-25-00232_revision_1.pdf]

GigaScience

# Chromosome-level assemblies of two hexaploid bamboos Thyrsostachys oliveri and Thyrsostachys siamensis provide a foundation for functional and comparative genomics studies

--Manuscript Draft--

| | |
|---|---|
| **Manuscript Number:** | GIGA-D-25-00232R1 |
| **Full Title:** | Chromosome-level assemblies of two hexaploid bamboos Thyrsostachys oliveri and Thyrsostachys siamensis provide a foundation for functional and comparative genomics studies |
| **Article Type:** | Research |
| **Funding Information:** | National Science and Technology Development Agency (P2251271) — Dr. Wirulda Pootakham |
| **Abstract:** | Background<br>Bamboo is an important non-timber forest resource with significant ecological and economic value. However, genomic resources for several bamboo lineages remain scarce.<br>Results<br>We present the first chromosome-scale genome assemblies for two economically important Thai woody bamboos, Thyrsostachys oliveri and Thyrsostachys siamensis. Using single-tube long-fragment read (stLFR) sequencing integrated with chromosome conformation capture (Hi-C) scaffolding, we assembled 35 pseudochromosomes spanning 990Mb (N50 = 22.18Mb) for T. oliveri and 1.14Gb (N50 = 19.45Mb) for T. siamensis, respectively. The T. oliveri and T. siamensis genome assemblies contain 51,191 and 67,483 predicted genes, with repeat contents of 50.9% and 48.8%, respectively. BUSCO completeness scores reached 97.4% for T. oliveri and 95.2% for T. siamensis, indicating near-complete coverage of the conserved gene space. Comparative analyses revealed that Thyrsostachys formed a sister group to Dendrocalamus, and the two Thyrsostachys species diverged approximately 5.4 million years ago. Both species have undergone a recent whole-genome duplication event. Gene family analysis identified species-specific gene families associated with root development and carbohydrate metabolism in T. oliveri, and with histone binding and proteasome pathways in T. siamensis. Moreover, homoeolog suppression was more frequent than single-homoeolog dominance, and subgenome-specific expression biases differed between species.<br>Conclusions<br>These genome assemblies fill a critical gap in bamboo genomics and provide a foundation for evolutionary, functional, and breeding research in hexaploid woody bamboos. They also serve as valuable reference genomes for comparative studies within and across bamboo species. |
| **Corresponding Author:** | Wirulda Pootakham<br>NSTDA: National Science and Technology Development Agency<br>Khlong Luang, Pathum Thani THAILAND |
| **Corresponding Author Secondary Information:** | |
| **Corresponding Author's Institution:** | NSTDA: National Science and Technology Development Agency |
| **Corresponding Author's Secondary Institution:** | |
| **First Author:** | Chaiwat Naktang |
| **First Author Secondary Information:** | |
| **Order of Authors:** | Chaiwat Naktang |
| | Supaporn Khanbo |
| | |

Powered by Editorial Manager® and ProduXion Manager® from Aries Systems Corporation

| | |
|---|---|
| | Poompat Phadphon |
| | Sonicha U-thoomporn |
| | Duangjai Sangsakru |
| | Chutima Sonthirod |
| | Pitchaporn Waiyamitra |
| | Sarawood Sungkaew |
| | Sithichoke Tangphatsornruang |
| | Wirulda Pootakham |
| **Order of Authors Secondary Information:** | |
| **Response to Reviewers:** | A Point-by-Point Response to Editor and Reviewer Comments.<br>Reviewer reports:<br><br>Reviewer #1: This manuscript by Naktang et al. presents the first chromosome-level genome assemblies for two economically important bamboo species, Thyrsostachys oliveri and T. siamensis. The generation of genomic resources for non-model, complex polyploid species like bamboo is a valuable endeavor for the plant genomics community.<br>However, despite the potential importance of the data, the manuscript in its current form suffers from several major deficiencies in its analytical depth, methodological reporting, and biological interpretation. The claims of producing "high-quality" genomes are not fully substantiated by state-of-the-art metrics and seem overstated in the current era of genomics. Furthermore, the comparative and functional analyses are largely superficial and fail to leverage this rich dataset to its full potential. The manuscript requires a substantial and thorough revision to address these fundamental issues before it can be considered for publication.<br><br>1. The claim of a "high-quality" assembly needs to be tempered. The assemblies are based on 150 bp short-read data (via stLFR), which inherently struggles to resolve complex repetitive regions. In the current era of Telomere-to-Telomere (T2T) genomics, assemblies derived from short reads cannot be unconditionally described as "high-quality" without significant caveats. The authors must discuss these limitations in the Discussion section.<br>Response: We thank the reviewer for this insightful comment. We agree and have revised the manuscript to temper our wording and to explicitly acknowledge short-read limitations. We now describe the assemblies as “chromosome-scale assemblies” rather than “high-quality.” We also added independent quality metrics to Supplementary Table S12: LAI = 7.75 / 7.10, Merqury QV = 55.85 / 52.5, and proteome-mode BUSCO completeness = 86.0% / 83.0% (embryophyta_odb10). We note in the Discussion that LAI < 10 indicates moderate repeat continuity and that, despite high per-base QV, long LTR-RTs and other complex repeats remain challenging for short reads in a hexaploid background. We have revised the Discussion section, and in lines 555–566 of the revised manuscript, we have added the following text:<br><br>“Compared with PacBio HiFi references, our stLFR+Hi-C assemblies achieve chromosome-scale, scaffold N50s and comparable BUSCO completeness (Supplementary Table S12) [22, 26, 78], while exhibiting lower repeat continuity (LAI) and base-level QV, as expected for short-read barcoded libraries. These trade-offs primarily affect long repetitive regions and do not preclude the gene-space and comparative analyses reported here. While Merqury QV indicates very high per-base accuracy, our LAI scores (<10) point to moderate repeat continuity, consistent with assemblies derived from 150-bp stLFR short reads. Long LTR retrotransposons and other complex or highly similar repeats are likely fragmented or collapsed, and some homoeologous regions in this hexaploid context may remain under-resolved. Future long-read (e.g., HiFi/ONT) and T2T scaffolding would further improve structural completeness and repeat resolution.”<br>2. The suite of genomic evaluation metrics is insufficient. Other standard measures, such as the LTR Assembly Index (LAI) for structural integrity and a base-level quality score (QV) from tools like Merqury, should be included to fully substantiate the quality claims. |

Powered by Editorial Manager® and ProduXion Manager® from Aries Systems Corporation

Response: Thank you for this suggestion. We have added LAI and Merqury QV metrics to Supplementary Table S12 and now report them in the Results section (lines 355–361). Whole-genome LAI scores are 7.75 for T. oliveri and 7.10 for T. siamensis, indicating moderate repeat continuity. Merqury base-level quality scores are QV 55.85 and QV 52.5, respectively. We also tempered our wording to describe the assemblies as “chromosome-scale assemblies,” and, in the Discussion section (lines 557–566), added a brief note indicating that LAI < 10 reflects limited resolution of long LTR retrotransposons relative to long-read/T2T assemblies.

3. The authors report genome-mode BUSCO scores but omit the equally important proteome-mode BUSCO scores. The latter directly assesses the completeness of the final gene annotation set and is essential for evaluating the annotation quality.
Response: Thank you for the suggestion. We have added proteome-mode BUSCO (on the predicted protein sets) alongside genome-mode, and we report both in the Results section (lines 354–356) and in Supplementary Table S12.

4. The reporting in Table 1 could be improved. The initial contig N50 is a more informative and standard metric for assessing baseline assembly fragmentation than the scaffold N75. It should be included for a direct comparison with the final scaffold N50.
Response: We revised Table 1 to include the initial contig N50 (pre-scaffold stLFR) alongside the final scaffold N50 (Hi-C for T. oliveri; RagTag for T. siamensis), enabling a direct baseline-to-final comparison. Specifically, contig N50s are 2,447,080 bp (T. oliveri) and 319,778 bp (T. siamensis), and final scaffold N50s are 22,175,826 bp and 19,454,152 bp, respectively. We retained Scaffold N75/L75/N90/L90 for completeness but now label all metrics by assembly stage (stLFR pre-scaffold vs Hi-C/RagTag final) to avoid ambiguity.

5. The manuscript lacks critical details regarding the manual curation and visualization of the Hi-C assembly. The authors state that "Manual inspection and Hi-C visualization tools were employed", but do not specify the criteria used for manual refinement or the names of the software. Furthermore, the methods should explicitly state how the Hi-C contact map in Supplementary Fig. S7 was generated and plotted. This information is essential for reproducibility.
Response: Thank you for this comment. The Methods (lines 168–175) have been revised to specify the software, the manual-refinement criterion, and how the contact map in Supplementary Fig. S3 was generated and plotted. Briefly, the genome-wide Hi-C contact matrix was generated with LACHESIS (Burton et al., 2013) by Biomarker Technologies (BMK). Manual inspection and limited adjustments were performed by BMK in CGAP (their in-house Hi-C visualization software), which displays higher interaction intensities as darker colors. As an example of the criterion used, when the interaction between Position 1 and Position 3 was stronger than that between Position 2 and Position 3 within a local block, the small interval between Positions 1 and 2 was flipped to restore the expected near-diagonal cis signal. Supplementary Fig. S3A shows the map before this flip and S3B shows the result after the flip. These details have been added to the Methods and the Supplementary figure legend for reproducibility.

6. The raw data appears to be inaccessible. The SRA Experiment accession SRX28190747 (corresponding to run SRR32915794 for T. oliveri) is listed as not public.
Response: Thank you for flagging this. The SRA records have now been set to public (as of 4 September 2025). The T. oliveri stLFR dataset SRX28190747 / SRR32915794 is visible and downloadable, and all other runs have likewise been released.

7. The comparative genomic analysis is disappointingly limited. While the authors cite the landmark Ma et al. (2024) paper and adopt its methodology for triad identification, they stop short of the most critical comparison. A fundamental, yet missing, analysis is to determine if the A, B, and C subgenomes in Thyrsostachys are orthologous to the core A, B, and C subgenomes of other hexaploid bamboos described in that study.
Response: We agree and now provide a subgenome-level orthology test across hexaploid bamboos. We compiled representative proteomes for Thyrsostachys (this study) and three hexaploids—Melocanna baccifera, Bonia amplexicaulis, and Dendrocalamus sinicus and ran OrthoFinder to infer orthogroups and gene trees. For

Powered by Editorial Manager® and ProduXion Manager® from Aries Systems Corporation

single-copy orthogroups represented across species, we labeled tips by subgenome (A/B/C) and species and assessed whether sequences cluster by subgenome across taxa (i.e., A-of-Thyrsostachys groups with A-of-Melocanna, A-of-Bonia, A-of-Dendrocalamus; analogously for B and C). The resulting trees show consistent A-with-A, B-with-B, and C-with-C clustering (see Supplementary Fig. S11), supporting orthology between the Thyrsostachys subgenomes and the core A/B/C subgenomes reported previously. We have added this result to the Results section (lines 437–445).

8. The analysis focuses heavily on species-specific differences, neglecting the evolution of the Thyrsostachys genus as a lineage. At a minimum, the authors should identify and discuss the gene families that are specific to the genus (shared by both species but absent in other taxa) and those that expanded or contracted in the common ancestor of Thyrsostachys.
Response: We thank the reviewer for this insightful suggestion. In the revised manuscript, we have expanded our analysis to include gene family evolution at the Thyrsostachys crown node, thereby addressing lineage-level changes in addition to species-specific differences. Detailed explanations of the results are provided in lines 458–472, with additional discussion in lines 612–616.

9. The whole-genome duplication (WGD) analysis, only relying on a 4DTv distribution plot. An analysis using synonymous substitution rates (Ks) derived from syntenic gene blocks would provide a clearer signal and is standard practice for a more robust WGD inference.
Response: We agree. We added a Ks-based analysis using syntenic anchor pairs from MCScanX (add_ka_and_ks_to_collinearity.pl). The Ks show clear bimodality with peaks at 0.150/0.516 in T. oliveri and 0.152/0.533 in T. siamensis, mirroring the 4DTv peaks (0.056/0.208 and 0.059/0.231, respectively). Inter-species Ks values recapitulate the same distance ranking as 4DTv (lowest between T. oliveri and T. siamensis), reinforcing the inference of a shared, relatively recent WGD. These additions have been incorporated into the Methods (lines 294–299), Results (lines 419–430, Fig. 2A; Supplementary Fig. S10;), and Discussion (lines 583–585) sections of the revised manuscript.

10. The biological context is underdeveloped. The manuscript provides only general statements about bamboo's utility but fails to frame the genomic analysis around any specific, unique biological or ecological traits of the Thyrsostachys genus.
Response: We appreciate your constructive feedback. In the revised Introduction, we now include specific details about the biological distinctiveness of Thyrsostachys oliveri and T. siamensis. These additions strengthen the rationale for conducting the genomic analyses presented in this study. This context has been incorporated into the Introduction section (lines 78–105).

11. The analysis of homeolog expression is merely descriptive and lacks biological interpretation. To provide meaningful insight, the authors must perform functional enrichment analyses (e.g., GO/KEGG) on the genes within each expression bias category (e.g., A-dominant, C-suppressed).
Response: We agree and have added a full functional-enrichment analysis stratified by homoeolog bias category. Specifically, we performed GO enrichment tests for all six categories (A/B/C-dominant; A/B/C-suppressed) in both tissues (leaf, root) of T. oliveri and T. siamensis. The new Results subsection “Functional enrichment of homoeolog bias categories” (lines 487–511) summarizes the biological interpretation, and Supplementary Table S10 provides full term lists and statistics.

12. The characterization of subgenome composition is incomplete. The authors should provide basic statistics, including the number of genes on each subgenome and the proportion that form 1:1:1 triads. Crucially, the manuscript must also quantify and discuss the genes that do not form triads (e.g., duplets, singlets, copy number variants), as the dynamics of gene loss and retention are central to polyploid evolution.
Response: We appreciate this suggestion and have added a comprehensive subgenome-composition analysis for both species (Results; Supplementary Table S11). We added a comprehensive subgenome-composition analysis for both species. Using the A–B–C subgenome assignments, we recorded, for each homoeolog correspondence set, whether copies were present in subgenomes A, B, and C and summarized these subgenome-presence classes as: triads (present in A, B, and C;

Powered by Editorial Manager® and ProduXion Manager® from Aries Systems Corporation

1:1:1), duplets (present in exactly two subgenomes), singletons (present in one), and other multi-copy configurations (including CNVs; any class in which a present subgenome contributes >1 copy). This context has been incorporated into the Results section, under "Subgenome composition and gene retention in Thyrsostachys" (lines 512–532).

13. The authors should ensure that all supplementary figures and tables are cited within the main body of the text to properly link the evidence to the narrative.
Response: We have carefully reviewed the main text and ensured that all supplementary figures and tables are now appropriately cited at relevant points in the manuscript.

14. A comprehensive check of(Akhunova et al. 2010) the reference list is required. Several entries appear to be incomplete or incorrectly formatted (e.g., references [34], [40], [52]), with inconsistencies in style throughout the bibliography. The entire reference list must be carefully reviewed and formatted according to the journal's guidelines.

Response: We have thoroughly reviewed and revised the entire reference list to ensure that all entries are complete and consistently formatted according to the journal’s guidelines.

Reviewer #2: This article provides a valuable resource by establishing reference genomes for two Thyrsostachys bamboo species, contributing significantly to the fields of bioinformatics, molecular biology, and evolutionary research. The study comprehensively investigated the genomics, comparative genomics, and comparative transcriptomics of Thyrsostachys oliveri and T. siamensis. Despite the strong data presented in this article, there is space for improvement in the depth of research. I have outlined some comments for the authors to address and revise accordingly.

（1） Line 35: In cases where the genus name is repeated, it is abbreviated the second time. Please check and revise other texts in your manuscript.
Response: We have carefully reviewed and revised the manuscript accordingly.

（2） Line 76-85: Can the authors emphasize the urgent need for conducting genome sequencing of Thyrsostachys oliveri and T. siamensis? Alternatively, describe the unique features that require research in genomics, comparative genomics, and comparative transcriptomics. Of course, this is not to question the work you all do.
Response: We appreciate the reviewer’s thoughtful suggestion. We have revised the Introduction to emphasize the urgent need for genome sequencing of Thyrsostachys oliveri and T. siamensis and to explicitly highlight their unique morphological and ecological traits, along with the current lack of genomic resources for this genus. This additional context has been incorporated into the Introduction section (lines 78–105).

（3） Line 89: Phyllostachys heterocycla and P. edulis are one species. The latter scientific name is accepted.
Response: We have corrected it.

（4） Line 90: I think authors should check more details about the genomes which had been published. Genomes of several species are not the first-time publised in ma et al. (2024). The article about the genomes of Bambusa odashimae, Raddia distichophylla, and Phyllostachys violascens should also be cited.
Response: We have cited the appropriate articles reporting the genomes of Bambusa odashimae, Raddia distichophylla, and Phyllostachys violascens as recommended.

（5） Line 121: "high-molecular-weight" should be replaced with the abbreviation "HMW".
Response: We have revised it.

（6） Line 241-242 and Line 396-397: If the genus names do not appear consecutively, please use the full name.
Response: We have revised it.

| | |
|---|---|
| | ( 7 ) Line335: In Table 1, I think the authors should provided LTR Assembly Index (LAI) for assessing genome assembly quality. The LAI can be obtained from LTR_retriever.<br>Response: Thank you for this suggestion. We have added LAI and Merqury QV metrics and now report them in the Results section (lines 355–361). Whole-genome LAI scores are 7.75 for T. oliveri and 7.10 for T. siamensis, indicating moderate repeat continuity. Merqury base-level quality scores are QV 55.85 and QV 52.5, respectively (Supplementary Table S12). We also tempered our wording to describe the assemblies as “chromosome-scale assemblies,” and added a brief Discussion note (lines 555–566) acknowledging that LAI < 10 reflects limited resolution of long LTR retrotransposons relative to long-read/T2T assemblies.<br><br>( 8 ) Line 411: There are two Fig. S5s (Fig. S5A and Fig. S5B), but what your reference is Fig. S5. I think they should be revised as Fig. S5 nad Fig. S6, respectively. The comments are also for Fig. S6A and Fig. S6B.<br>Response: Thank you for pointing out the duplicated numbering. We have split the former panels into standalone supplementary figures and renumbered them as follows: former Fig. S5A → Fig. S12; former Fig. S5B → Fig. S13; former Fig. S6A → Fig. S14; former Fig. S6B → Fig. S15. All in-text citations and figure captions have been updated accordingly.<br><br>( 9 ) The font used in Supplementary Tables is rather strange and needs to be adjusted.<br>Response: We have revised the font style and formatting in all Supplementary Tables.<br><br>( 10 ) In Disscussion section, when discussing the expansion and contraction of gene families, authors should consider specific biological characteristics of these two bamboo species or their unique evolutionary status, and may also integrate discussions with other bamboo species, rather than speaking in general terms.<br>Response: We thank the reviewer for this helpful suggestion. We have revised the Discussion section to explicitly link gene family expansions and contractions with the unique biological traits and evolutionary context of T. oliveri and T. siamensis. Furthermore, we have compared these patterns with gene family dynamics reported in other woody bamboo species (Discussion, lines 598–608).<br><br>( 11 ) I think the authors should provide photos of Thyrsostachys oliveri and T. siamensis, whether they are included in the main text or in the supporting materials.<br><br>Response: Thank you for the suggestion. We have added photographs of Thyrsostachys oliveri and T. siamensis to the Supplementary Materials (Supplementary Fig. S1-S2).<br><br><br>Reviewer #3: This manuscripts presents chromosome-scale genomes of two economically significant Thai woody bamboos (Thyrsostachys oliveri and T. siamensis) using an integrated approach of single-tube long-fragment read (stLFR) sequencing and Hi-C scaffolding. The authors performed comprehensive assemble, gene annotation, evolutionary analyses, and preliminary investigation of homoeolog expression patterns. The release of these genomes, combined with previously published bamboo genomes, will facilitate deeper exploration of bamboo-specific biological traits. I recommend acceptance after minor revisions, with the following suggestions:<br><br>Lines 282-284: Clarify why D. sinicus was selected as the reference framework: Is this solely due to its allele-aware chromosome-level quality, or also because of its closer phylogenetic affinity with Thyrsostachys? Provide supporting evidence for this choice.<br><br>Response: We selected D. sinicus as the primary reference for three reasons:<br>1. Published plastome phylogenies place Thyrsostachys within Bambusinae and resolve T. siamensis as sister to Dendrocalamus birmanicus, indicating close affinity between the genera (consistent with our Fig. 2) [DOI: 10.1080/23802359.2021.1934138]<br>2. Assembly quality and ploidy match. D. sinicus is one of the hexaploid woody |

| | |
|---|---|
| | bamboos with a chromosome-level genome assembled using long reads plus Hi-C; hexaploid species in that study were consistently anchored to 35 pseudo-chromosomes. This provides a like-for-like framework for subgenome-aware synteny. [DOI: 10.1038/s41588-024-01683-0]<br>3. Genome-scale compatibility. Reported hexaploid bamboo genome sizes average ~1.12 Gb, matching our Thyrsostachys assemblies (~1.0–1.14 Gb), which facilitates 1:1 collinearity assessment. [DOI: 10.1038/s41588-024-01683-0]<br>We also validated this choice empirically: whole-genome dot plots between our Thyrsostachys assemblies and D. sinicus show extensive 1:1 collinearity with limited large-scale rearrangements (now Supplementary Fig. S4). Together, the close phylogenetic relationship, chromosome-scale hexaploid reference, and syntenic congruence justify using D. sinicus as the reference framework.<br>We added the following sentence to the Methods section (lines 305–308) for clarity: "We selected D. sinicus as the synteny framework owing to its close placement to Thyrsostachys in published plastome phylogenies [74], together with its chromosome-level hexaploid assembly and strong collinearity with our genomes (Supplementary Fig. S4)."<br><br>About stLFR Methodology: Given that most published bamboo genomes used PacBio long-read technology, please directly compare key assembly metrics (e.g., N50, BUSCO completeness) between your stLFR-based assemblies and phylogenetically proximate species from published bamboo genomes. This will explicitly demonstrate the competitiveness of stLFR with PacBio technology.<br>Response: We added Supplementary Table S12 benchmarking our chromosome-scale stLFR assemblies against representative PacBio genomes (the Phyllostachys edulis HiFi pangenome, P. violascens 'Prevernalis', and Dendrocalamus latiflorus). The table reports contiguity (contig/scaffold N50), anchoring, BUSCO (genome and proteome mode), LAI, and QV. In the Discussion (lines 555–566), we summarize the comparison and explicitly acknowledge technology-driven differences.<br><br><br>Lines 337-345: Strengthen the comparative analysis based on gene features (e.g., gene length, exon/intron length, GC content….) with the 11 recently published bamboo genomes, highlighting conserved and divergent features in T. oliveri and T. siamensis.<br><br>Response: Thank you for the suggestion. We have added a side-by-side comparison of gene features for T. oliveri and T. siamensis against the 11 recently published bamboo genomes in the Results section (lines 372–376), with the updated Supplementary Table S2 including species, gene count, average gene length, average exon number, median exon length, and average intron length. This highlights both conserved and divergent patterns:<br>Conserved: median exon size is tightly clustered across bamboos (~124–158 bp), and most species including T. oliveri have ~5 exons per gene.<br>Divergent in Thyrsostachys: average gene length is shorter in T. oliveri/T. siamensis (2.86/2.67 kb) than in other 11 published bamboo genome (~4.0–5.0 kb), and average intron length is also shorter (436/492 bp vs ~600–770 bp). T. siamensis has fewer exons per gene (4.27) than most bamboos (~5).<br><br>Lines 416-429: To reinforce the observed subgenome-specific expression biases, include one concrete example (e.g., evolutionarily significant gene) demonstrating subgenome-specific expression biases differing between species.<br>Response: We expanded the functional interpretation with GO enrichment across the six bias categories (Results: "Functional enrichment of homoeolog bias categories"). To provide the requested concrete example, we now highlight a mannose-6-phosphate isomerase (PMI; GO:0004476) triad that shows species-specific subgenome bias B-dominant in T. oliveri leaves but not B-dominant in T. siamensis.  Full term lists and statistics are provided in Supplementary Table S10.<br><br>Line 210: Please updated the outdated hyperlink to the currently accessible version: https://doi.org/10.6084/m9.figshare.24411913.v3<br>Response: Thank you for the suggestion. We have updated Line 210 to the versioned DOI and now cite the dataset as: "Figshare (DOI: 10.6084/m9.figshare.24411913.v3)."<br><br>About data Accessibility (Lines 161-168 & 199-217): While raw reads have been |

Powered by Editorial Manager® and ProduXion Manager® from Aries Systems Corporation

| | |
|---|---|
| | deposited, gene annotation files (GFF format) should be submitted alongside the genome assemblies to ensure full data utility.<br>Response: We have deposited the complete annotation sets in GigaDB alongside both assemblies, including GFF3, protein (.faa), CDS (.fna), functional-annotation tables, and a README with checksums. |
| **Additional Information:** | |
| **Question** | **Response** |
| Are you submitting this manuscript to a special series or article collection? | No |
| **Experimental design and statistics**<br><br>Full details of the experimental design and statistical methods used should be given in the Methods section, as detailed in our Minimum Standards Reporting Checklist. Information essential to interpreting the data presented should be made available in the figure legends.<br><br>Have you included all the information requested in your manuscript? | Yes |
| **Resources**<br><br>A description of all resources used, including antibodies, cell lines, animals and software tools, with enough information to allow them to be uniquely identified, should be included in the Methods section. Authors are strongly encouraged to cite Research Resource Identifiers (RRIDs) for antibodies, model organisms and tools, where possible.<br><br>Have you included the information requested as detailed in our Minimum Standards Reporting Checklist? | Yes |
| **Availability of data and materials**<br><br>All datasets and code on which the conclusions of the paper rely must be either included in your submission or deposited in publicly available repositories (where available and ethically appropriate), referencing such data using | Yes |

| | |
|---|---|
| a unique identifier in the references and in the “Availability of Data and Materials” section of your manuscript.<br><br>Have you have met the above requirement as detailed in our Minimum Standards Reporting Checklist? | |
| GigaScience has policies and guidelines in place for the use of generative AI-writing tools such as ChatGPT. If you have used such writing tools to assist with writing the manuscript this must be declared and cited in the text. Authors should not list AI-writing tools and other AI-assisted technologies as an author or co-author and should acknowledge that they are fully responsible for text generated or refined by AI-writing tools.<p><br><br>A summary of use (particularly in the introduction or among methods) needs to be included at the end of the paper, and the outputs should also be included as a supplementary file hosted in GigaDB or other open repositories. Please <a href=https://academic.oup.com/gigascience/pages/editorial_policies_and_reporting_standards target="_new" > read our guidelines for more information. </a> <p><br><br>By submitting to GigaScience, you are aware of the journal's AI-writing tools policy, and if you have declared use of such tools below, you have acknowledged this where appropriate in your manuscript and have made a summary of use and outputs available. </b><p><br><b>AI-assisted writing tools have been used in the preparation of this manuscript? | No |

Powered by Editorial Manager® and ProduXion Manager® from Aries Systems Corporation

Manuscript
Click here to access/download;Manuscript;GigaScience manuscript GIGA-D-25-00232 (bamboos)_final.docx



# Chromosome-level assemblies of two hexaploid bamboos *Thyrsostachys oliveri* and *Thyrsostachys siamensis* provide a foundation for functional and comparative genomics studies

Chaiwat Naktang[1, †], Supaporn Khanbo[1, †], Poompat Phadphon[1], Sonicha U-thoomporn[1], Duangjai Sangsakru[1], Chutima Sonthirod[1], Pitchaporn Waiyamitra[1], Sarawood Sungkaew[2], Sithichoke Tangphatsornruang[1], Wirulda Pootakham[1,*]

[1]National Omics Center, National Center for Genetic Engineering and Biotechnology, National Science and Development Agency, Pathum Thani, Thailand

[2]Department of Forest Biology, Faculty of Forestry, Kasetsart University, Bangkok, Thailand

[†]Equal contribution and shared first authorship.

*Corresponding author:

Wirulda Pootakham (wirulda@alumni.stanford.edu)

Wirulda Pootakham [0000-0001-6721-6453]

Chaiwat Naktang [0000-0003-1400-8508]

Poompat Phadphon [0000-0002-7425-8579]

Chutima Sonthirod [0009-0009-3116-0078]

Pitchaporn Waiyamitra [0000-0001-9694-8565]

Sarawood Sungkaew [0000-0003-0389-7223]

Sithichoke Tangphatsornruang [0000-0003-2673-0012]

## Abstract

### Background

Bamboo is an important non-timber forest resource with significant ecological and economic value. However, genomic resources for several bamboo lineages remain scarce.

**Results**

We present the first chromosome-scale genome assemblies for two economically important Thai woody bamboos, *Thyrsostachys oliveri* and *Thyrsostachys siamensis*. Using single-tube long-fragment read (stLFR) sequencing integrated with chromosome conformation capture (Hi-C) scaffolding, we assembled 35 pseudochromosomes spanning 990 Mb (N50 = 22.18 Mb) for *T. oliveri* and 1.14 Gb (N50 = 19.45 Mb) for *T. siamensis*, respectively. The *T. oliveri* and *T. siamensis* genome assemblies contain 51,191 and 67,483 predicted genes, with repeat contents of 50.9% and 48.8%, respectively. BUSCO completeness scores reached 97.4% for *T. oliveri* and 95.2% for *T. siamensis*, indicating near-complete coverage of the conserved gene space. Comparative analyses revealed that *Thyrsostachys* formed a sister group to *Dendrocalamus,* and the two *Thyrsostachys* species diverged approximately 5.4 million years ago. Both species have undergone a recent whole-genome duplication event. Gene family analysis identified species-specific gene families associated with root development and carbohydrate metabolism in *T. oliveri*, and with histone binding and proteasome pathways in *T. siamensis*. Moreover, homoeolog suppression was more frequent than single-homoeolog dominance, and subgenome-specific expression biases differed between species.

**Conclusions**

These genome assemblies fill a critical gap in bamboo genomics and provide a foundation for evolutionary, functional, and breeding research in hexaploid woody

bamboos. They also serve as valuable reference genomes for comparative studies within and across bamboo species.

**Keywords:** *Thyrsostachys oliveri*, *Thyrsostachys siamensis,* chromosome-scale genome assembly, Annotation, Hi-C, stLFR

## Introduction

Bamboo is one of the most important non-timber forest resources that are widely distributed across the subtropical and tropical regions of Asia, Africa, and Latin America [1-3], covering roughly 31–35 million ha (≈ 0.8–1% of global forest area) [4, 5]. Bamboos constitute the subfamily Bambusoideae of Poaceae (Gramineae) and include about 1,670 species in 125 genera [3, 6, 7]. They are classified into four monophyletic lineages with distinct ploidy levels: herbaceous bamboos (2n = 20–24, diploid); temperate woody bamboos (2n = 46–48, tetraploid); neotropical woody bamboos (2n= 40–48, tetraploid); and palaeotropical woody bamboos (2n = 70–72, hexaploid) [8-11]. Among these, woody bamboos show a wide range in genome sizes, chromosome number, and ploidy [8]. Because of rapid growth and ease of propagation, bamboos provide substantial economic and ecological benefits. Their uses span food, medicine, bio-energy, building timber, furniture, and handicrafts, and they also help restore degraded landscapes and mitigate climate change impacts [12-14].

Bamboo plays a significant economic role in many developing countries, particularly in Asia [15]. In Thailand, roughly 69 species across 17 genera have been recorded [14, 16]. *Thyrsostachys* is a small bamboo genus native to Thailand and

Myanmar, comprising only two species*:* *Thyrsostachys oliveri* (NCBI:txid338531) and *Thyrsostachys siamensis* (NCBI:txid338532). *T. oliveri* is a tropical clumping bamboo characterized by short rhizomes, straight and slender culms. These culms also have high branches with dense, small leaves, which give the entire bamboo cluster a slender and elegant appearance [17, 18]. Its culms are widely used locally for construction, furniture, and household products [17]. This bamboo is also cultivated as an ornamental plant [19]. *T. siamensis* is one of the most useful Thai bamboos, yielding wood and good edible shoots [19]. The species is characterized by its compact clumps, branching from upper mid-culm, and small leaves [19]. It grows rapidly, develops thicker culm walls, accumulates larger biomass, and supplies material for construction and handicrafts [14, 20]. Both species share a characteristic erect while differing in key traits such as culm wall thickness, branching patterns, and shoot productivity. Despite their ecological and economic significance, they lack genomic resources, leaving many key traits poorly understood at the molecular level.

Although reference genomes have been developed for multiple bamboo species including *Dendrocalamus brandisii* [21], *Dendrocalamus latiflorus* [22], *Phyllostachys edulis* [13, 23], *Bambusa odashimae* [24] , *Raddia distichophylla* [25], *Phyllostachys violascens* [26], and others at various ploidy levels [27], genomic resources for *T. oliveri* or *T. siamensis* have not yet been established. The absence of reference genomes for *Thyrsostachys* now represents a critical gap. Therefore, there is an urgent need to obtain a reference genome for the genus. Generating chromosome-scale reference genomes for this genus will therefore provide essential resources for investigating lineage-specific adaptations, uncovering the evolutionary history and enabling functional studies across multiple Bambusoideae lineages. These genomes will also help identify genes or proteins that control desirable traits, providing

a foundation for trait-specific genetic modification programs in bamboos and offering new insights into the genetic basis of growth form, morphological diversity, and ecological adaptation. In addition, they will facilitate comparative genomics and transcriptomics with other woody bamboos and support conservation, breeding, and sustainable utilization efforts.

Here, we sequenced and assembled two Thai hexaploid species, *T. oliveri* and *T. siamensis*, using single-tube long-fragment reads (stLFR) technology [28] combined with chromosome conformation capture (Hi-C) scaffolding. The resulting chromosome-level assemblies provide crucial resources for investigating genomic organization and evolution in these species. Our work fills a critical gap in bamboo genomics and lays the groundwork for future studies aimed at genetic improvement, molecular breeding, and conservation of economically important bamboos.

## Methods

### Plant materials and DNA/RNA isolation

For genome sequencing, young leaf samples were collected from *T. oliveri* and *T. siamensis* plants grown at Kasetsart University, Bangkok Province, Thailand (13.8423° N, 100.5771° E). Immediately after collection, healthy leaves were flash-frozen in liquid nitrogen and stored at -80°C. High-molecular-weight (HMW) DNA was extracted using the Qiagen Genomic-tip 100/G following the manufacturer's instructions (Qiagen, Hilden, Germany). DNA quality and quantity were subsequently evaluated using the Pippin Pulse Electrophoresis System (Sage Science, Beverly, USA) and the Qubit 4 Fluorometer (RRID:SCR_018095) (Thermo Fisher Scientific, Waltham, USA), respectively, prior to library construction. For downstream annotation purposes, total RNA was extracted from leaf and root tissues of the same individual

used for RNA sequencing, following the protocol outlined in [29]. Briefly, RNA was isolated using a CTAB buffer and a 25:24:1 phenol:chloroform:isoamyl alcohol mixture, then precipitated overnight with a quarter volume of 8M LiCl. The resulting RNA pellets were washed with 70% ethanol, air-dried, and resuspended in RNase-free water. RNA integrity was assessed using the Fragment Analyzer system (RRID:SCR_019417) (Agilent, Santa Clara, USA) before RNA sequencing library construction. Representative photographs of *T. oliveri* and *T. siamensis* are shown in Supplementary Fig. S1-S2.

**Genome and transcriptome sequencing**

A preliminary draft genome assembly was generated by first constructing a stLFR (single-tube long fragment read) sequencing library. This library was prepared using 10 ng of HMW DNA and the MGIEasy stLFR Library Prep Kit (MGI Tech, Shenzhen, China), following the manufacturer's protocol. Briefly, this process involved transposon-mediated insertion into the HMW DNA, incubation with clonally barcoded beads for random priming, and subsequent fragmentation into sub-fragments (<1 kb). Following adapter ligation and PCR amplification, the library was ready for sequencing. For transcriptome analysis, 200 ng of total RNA was used to generate a library with the MGIEasy RNA Library Prep Kit v3.0 (MGI Tech, Shenzhen, China), adhering to the manufacturer's instructions. Both the stLFR and RNA libraries were then sequenced on the DNBSEQ-G400 (RRID:SCR_017980) platform using the MGISEQ-2000RS Sequencing Flow Cell v3.0 (MGI Tech, Shenzhen, China).

**Hi-C library preparation and sequencing**

To achieve chromosome-level scaffolding of the initial assembly, Biomarker Technologies (Beijing, China) utilized a chromosome conformation capture (Hi-C)

technique. For Hi-C library preparation using *T. oliveri* tissue, the following procedure was employed. Briefly, chromatin from fresh tissue was crosslinked with formaldehyde, and the fixed chromatin was digested with the restriction endonuclease HindIII [30]. The resulting fragments were then treated to incorporate biotinylated nucleotides at their 5′ ends, followed by ligation to form chimeric junctions representing spatially proximate chromatin regions. After reversing the crosslinks, the DNA was purified and sheared into fragments ranging from 300 to 700 base pairs. Biotinylated fragments were then enriched using streptavidin beads. The purified Hi-C fragments were used to construct Illumina-compatible sequencing libraries, which were subsequently sequenced on the Illumina HiSeq X Ten (RRID:SCR_016385) (PE150; Illumina, San Diego, CA, USA).

**Genome assembly and Hi-C scaffolding**

Preliminary draft assemblies for both genomes were generated from 150-bp paired-end stLFR sequencing data using stLFRdenovo v1.0.5 [31] with default settings. Subsequently, the initial draft assembly of *T. oliveri* was scaffolded to chromosome level utilizing Hi-C data. This Hi-C scaffolding was conducted by Biomarker Technologies Corporation (Beijing, China). The draft assembly and Hi-C reads were processed using HiRise v2.1.9 (RRID:SCR_017788), a pipeline optimized for proximity ligation data [32]. Hi-C reads were aligned to the draft assembly using BWA v0.7.17 (RRID:SCR_010910) [33] while data filtering and quality assessment were performed with HiC-Pro v2.10.0 (RRID:SCR_017643) [34]. The HiRise software analyzed Hi-C read pair distributions to detect and correct misjoins, identify prospective joins, and produce a chromosome-level assembly. For visualization, a genome-wide Hi-C contact matrix was generated with LACHESIS

(RRID:SCR_017644) [35] and plotted in CGAP (Biomarker Technologies' in-house Hi-C visualization software), which displays higher interaction intensities as darker colors. Manual inspection and limited adjustments were performed in CGAP; for example, when the interaction between Position 1 and Position 3 was stronger than that between Position 2 and Position 3 within a local block, the short interval between Positions 1 and 2 was flipped to restore the expected near-diagonal cis signal (Supplementary Fig. S3A–B). *T. siamensis* was subsequently scaffolded using the *T. oliveri* chromosome-level assembly as a reference with the RagTag software v1.1.0 (RRID:SCR_027293) [36, 37]. Both *T. oliveri* and *T. siamensis* genome assemblies have been deposited at NCBI under accession numbers JAWCWW000000000 and JBEFOJ000000000, respectively. The raw stLFR reads for *T. oliveri* and *T. siamensis* are available in the NCBI SRA database under accession numbers SRR32915794 and SRR32915889, respectively. The transcriptome data for *T. oliveri* were submitted under SRR33015685 and SRR33015684. The transcriptome data for *T. siamensis* was submitted under SRR33015804 and SRR33015803.

**Genome size estimation**

Genome size was estimated using two complementary approaches. First, the k-mer analysis was performed on raw stLFR reads using Jellyfish v2.2.10 (RRID:SCR_005491), and the resulting distributions were visualized with GenomeScope v2.0 (RRID:SCR_017014) [38, 39] (k = 21). Second, nuclear DNA content was estimated using flow cytometry. Fresh leaf tissues from *T. oliveri* and *T. siamensis* were processed following the protocol described in [40], using Galbraith's buffer for nuclear isolation. Nuclei were stained with 50 μg/mL propidium iodide

(Thermo Fisher Scientific). Maize (*Zea mays*) leaf sample was used as the reference standard for DNA content.

**Quality assessment of the genome assembly**

To evaluate the quality of the final *T. oliveri* and *T. siamensis* assemblies, both short-read DNA (stLFR) and RNA sequencing data were mapped to each respective assembly. Specifically, stLFR reads were aligned using BWA v0.7.17 [33], and RNA sequencing reads were aligned using HISAT2 v2.2.0 (RRID:SCR_015530) [41]. Furthermore, assembly and proteome completeness was assessed using the Benchmarking Universal Single-Copy Orthologs (BUSCO) pipeline v5.4.4 (RRID:SCR_015008) [42], which examined the presence of conserved orthologous genes against the plant-specific Embryophyta OrthoDB database release 10 [43]. In addition, we computed the LTR Assembly Index (LAI) with LTR_retriever v3.0 (RRID:SCR_017623) [44] and estimated base-level quality (QV) with Merqury v1.3 [45] (RRID:SCR_022964).

**Repetitive sequence identification**

Repetitive elements were identified through both de novo and homology-based approaches. First, we generated a de novo repeat library using RepeatModeler (v2.0.3) (RRID:SCR_015027) [46], which incorporates multiple algorithms (RECON, RepeatScout and LtrHarvest/Ltr_retriever) to detect and characterize repeat boundaries. The resulting consensus sequences were aligned against the NCBI GenBank non-redundant protein database (nr) via BLASTX (e-value ≤ $1e^{-6}$) (RRID:SCR_004870) to confirm the absence of large, non-TE protein-coding families. For homology-based identification, the assembled genome was scanned with

RepeatMasker v4.0.9_p2 (RRID:SCR_012954) [47] using the RepBase plant repeat database (RRID:SCR_021169) [48].

**Gene prediction and annotation**

To annotate protein-coding sequences, we employed both Evidence Modeler (EVM) software version 1.1.1 (RRID:SCR_014659) [49] and the MAKER2 (RRID:SCR_005309) pipeline [50] to identify protein-coding genes in the masked genome assembly. These approaches combined homology-based, RNA-based, and ab initio predictions. Transcript-based gene prediction was performed using RNA-seq data derived from leaf and root tissues. Raw reads were assembled using Trinity v2.9.1 (RRID:SCR_013048) [51] and clustered at 95% identity with CD-HIT v4.8.1 (RRID:SCR_007105) [52, 53]. For each cluster, the longest open reading frame (ORF) was identified and subsequently aligned to the assembled genome using PASA v2.5.3 (RRID:SCR_014656) [54] and GMAP v2020-09-12 (RRID:SCR_008992) [55]. To aid in gene annotation, protein sequences from related bamboo species, specifically *Dendrocalamus latiflorus* [56], *Dendrocalamus sinicus*, *Bonia amplexicaulis*, *Guadua angustifolia*, *Olyra latifolia*, and *Raddia guianensis* [57], were downloaded from publicly available databases and aligned to the genome assembly using AAT [58]. Ab initio gene predictions were generated with AUGUSTUS v3.2.1 (RRID:SCR_008417) [59], trained on data from the same reference species plus PASA transcript alignments. EVM was then used to integrate these lines of evidence—transcript, homology, and ab initio—assigning weights of 5 for PASA, 1 for GMAP, 0.5 for AAT, and 0.1 for AUGUSTUS.

In parallel, the MAKER2 pipeline was employed to merge ab initio predictions (using SNAP (RRID:SCR_007936) [60] and AUGUSTUS), protein homology, and

transcript-based evidence. After an initial MAKER run with default parameters, the resulting gene models were used to retrain SNAP and AUGUSTUS, followed by a second MAKER iteration to refine predictions. Low-confidence models were removed or flagged for manual inspection, and the final consensus gene models were output in GFF format. GFF files exhibiting the highest BUSCO completeness scores were ultimately selected for downstream analyses, ensuring robust and high-quality gene annotations. All predicted genes were functionally annotated using OmicsBox v2.0.10 [61] (RRID:SCR_018930). Protein sequences were aligned against the GenBank's non-redundant (NR) databases via BLASTP [62] with an e-value cutoff of $10^{-5}$. Gene Ontology (GO) terms were retrieved and assigned to the predicted gene models, while enzyme codes (EC) were extracted and mapped to KEGG pathway annotations. To annotate noncoding RNAs (ncRNAs), tRNAs were predicted using tRNAscan-SE v2.0 (RRID:SCR_008637) [63], and rRNA sequences were identified by aligning known rRNA references from closely related species using BLAST. Other ncRNAs, such as miRNAs and snRNAs, were identified by searching against the Rfam v14.1 database (RRID:SCR_007891) [64] with Infernal v1.1.5 (RRID:SCR_011809) [65] using default parameters.

**Comparative genomics and phylogenetic analyses**

To investigate the evolutionary relationships of *T. oliveri* and *T. siamensis* within the grass family, a comparative genomics study was conducted. OrthoFinder v2.5.5 (RRID:SCR_017118) [66] was used to identify orthologous groups among ten grass species, including nine bamboo species (*B. amplexicaulis*, *G. angustifolia*, *P. edulis* (Moso bamboo), *T. oliveri*, *T. siamensis*, *O. latifolia*, *R. guianensis, D. sinicus* and *D. latiflorus*), other grasses (*Brachypodium distachyon* (a model grass species), and a

cereal crop (*Oryza sativa* (rice)). A set of single-copy orthologous proteins was extracted, and these sequences were aligned and trimmed using MUSCLE v3.8 (RRID:SCR_011812) [67] and trimAl (RRID:SCR_017334) [68], respectively. Catsequences software [69] was used to concatenate the alignment blocks. The best substitution model for each block was evaluated using ModelTest-NG (RRID:SCR_026633) [70]. A maximum-likelihood phylogenetic tree was constructed using RAxML-NG (RRID:SCR_022066) [71] with 1000 bootstrap. *O. sativa* was designated as an outgroup.

The divergence time of the 11 species was estimated using BEAST v.2.7.7 (RRID:SCR_017307) [72]. Two independent MCMC tree searches were run for 10,000,000 generations, with a sampling frequency of 1,000 generations. The estimations were run under JTT substitution model together with optimized relaxed clock model and Birth-Death tree prior. The Bambusoideae cf. Chusquea fossil (35-90 Mya;[73]) and the fossilized phytoliths and cuticle of Ehrhartoideae-Oryzeae (67-90 Mya;[74]) were used to calibrate the crown node of Bambusoideae and BEP clade respectively following [75]. The crown node of Bambuseae estimated to be 28.24 (19.82-38.95) Mya by Zhang et al. (2016) was included as a secondary calibration point. To assess the convergence of parameters, Tracer v1.7.1(RRID:SCR_019121) [76] was utilized to check the effective sample sizes (ESSs). ESSs of all parameters were above 200 after a burn-in period of 25%. The maximum clade credibility tree was generated by TreeAnnotator v2.7.7 [72].

Gene family expansions and contractions were identified using CAFE v5 (RRID:SCR_018924) [77], to detect significant changes in gene family size across the phylogeny ($p < 0.05$). This analysis employed a probabilistic model based on a time-calibrated phylogenetic tree and estimated gene birth-death rates ($\lambda$) using a

maximum-likelihood approach. Functionally, Gene Ontology (GO) analyses were performed to annotate expanded and contracted gene families.

**The analysis of genome synteny**

Collinearity analyses were performed using McscanX (RRID:SCR_022067) [78] to investigate syntenic relationships within the *T. oliveri* and *T. siamensis* genomes, and between *T. oliveri* and *T. siamensis*, *B. amplexicaulis*, *D. sinicus*, *D. latiflorus*, *O. latifolia*, *P. edulis*, and *O. sativa*. Putative paralogous gene pairs were identified by aligning *T. oliveri* amino acid sequences against themselves and those of *T. siamensis* using BLASTP with an E-value threshold of $10^{-10}$. Intragenic homeologous blocks were defined as regions containing ten or more collinear or nearly collinear paralogous gene pairs, with a maximum of six intervening non-paralogous genes. The resulting intragenic homeologous blocks were visualized using CIRCOS v0.69.8 (RRID:SCR_011798) [79]. The synteny of *Thyrsostachys* subgenome and *Oryza sativa* was examined using NGenomeSyn 1.41 [80] to identify similarities in their genomic structures. Ks for syntenic anchor pairs was estimated using the MCScanX script add_ka_and_ks_to_collinearity.pl. For the subgenome-level test, we repeated this pipeline on single-copy orthogroups from the five hexaploids (*Thyrsostachys*, *Melocanna baccifera*, *Bonia amplexicaulis*, *Dendrocalamus sinicus*), labeled sequences by A/B/C, and asked whether genes with the same label formed a monophyletic clade across species.

**Subgenome Identification**

To facilitate subgenome classification within the newly assembled *T. oliveri* and *T. siamensis* genomes, the established subgenome assignments of the *D. sinicus*

reference genome, previously determined via allele-aware chromosome-level analysis [27], were adopted as a reference framework. We selected *D. sinicus* as the synteny framework owing to its close placement to *Thyrsostachys* in published plastome phylogenies [81], together with its chromosome-level hexaploid assembly and strong collinearity with our genomes (Supplementary Fig. S4). In practice, protein-coding genes from the *T. oliveri* and *T. siamensis* assemblies were aligned to those of the *D. sinicus* reference using the jcvi synteny pipeline (v1.1.17) (RRID:SCR_018403) [82]. Syntenic gene blocks were identified, with the '–quota' parameter adjusted to reflect genome ploidy. By comparing the resulting syntenic blocks to the previously characterized subgenomes of *D. sinicus*, subgenome identities were inferred for the *T. oliveri* and *T. siamensis* assemblies. This methodology enabled the projection of the established *D. sinicus* subgenome structure onto the target genomes, thereby minimizing methodological redundancy.

**Expression bias between subgenomes**

To investigate subgenome-specific gene expression in hexaploid *T. oliveri* and *T. siamensis*, we utilized 1:1:1 gene triads, identified using the methodology of [27]. Briefly, we defined a triad as expressed when the sum of the A, B and C subgenome homoeologs had transcript per million (TPM)> 0.5 and standardized the relative expression of each homoeolog across the triad. The ternary diagrams were plotted using the R package ggtern [83]. To categorize homoeolog expression bias, we employed a method analogous to that used in wheat [84]. Ideal normalized expression biases for six distinct categories were defined. The Euclidean distance (calculated using the R function rdist) between the observed normalized expression of each triad and each ideal category was determined. Each triad was subsequently assigned to

the category with the shortest Euclidean distance, representing its homoeolog expression bias, and this process was repeated for both leaf and root tissues.

## Results

### Genome assembly and evaluation

High-coverage stLFR libraries generated 408,544,881 reads (81.71 Gb) for *T. oliveri* and 401,766,290 reads (80.35 Gb) for *T. siamensis* (Supplementary Table S1). Genome size estimates for *T. oliveri* were 1.088 Gb by k-mer analysis and 1.226 Gb by flow cytometry, yielding an average of 1.157 Gb; for *T. siamensis*, estimated were 1.097 Gb (k-mer) and 1.247 Gb (flow cytometry), with a mean of 1.172 Gb (Supplementary Fig. S5–S6). Using the mean genome sizes (1.157 Gb for *T. oliveri*; 1.172 Gb for *T. siamensis*), the stLFR yields (81.71 Gb and 80.35 Gb) correspond to ~71x and ~69x sequencing depth, respectively. *De novo* assembly of the stLFR reads produced 32,505 contigs (N50 = 2.45 Mb; 990 Mb) for *T. oliveri* and 77,021 contigs (N50 = 0.32 Mb; 1.14 Gb) for *T. siamensis* (Table 1). Heterozygosity estimates were 0.98% for *T. oliveri* and 2.67% for *T. siamensis* (Supplementary Fig. S6).

For *T. oliveri*, 351 million Hi-C read pairs were used for scaffolding of the preliminary assembly to yield a scaffold N50 of 22.18 Mb and anchoring 81.4% (804 Mb) of sequence into 35 pseudo-chromosomes, matching 2n = 6x = 70 (Fig. 1A and Supplementary Fig. S7). For *T. siamensis*, RagTag scaffolding against the *T. oliveri* reference produced a 19.45 Mb scaffold N50 with 69.2% (789 Mb) of sequence anchored to the 35 *T. oliveri* pseudo-chromosomes (Table 1). Each assembly was partitioned into three homoeologous subgenomes (A, B, C). Extensive intra- and inter-subgenomic collinearity and large blocks aligning to the 12 *O. sativa* chromosomes are shown in Fig. 1A-C.

BUSCO analysis (Embryophyta odb10; 1,614 genes) recovered 97.4% complete genes in *T. oliveri* and 95.2% in *T. siamensis* (Table 1). Read-mapping rates further supported assembly accuracy: 98.83% / 98.10% of stLFR reads and 90.22% / 87.62% of RNA-seq reads mapped back to the *T. oliveri* and *T. siamensis* genomes, respectively. In proteome mode (embryophyta_odb10), BUSCO completeness was 86.0% for *T. oliveri* and 83.0% for *T. siamensis*, directly assessing annotation completeness. Together with LAI scores of 7.75 and 7.10 and Merqury QV values of 55.85 and 52.5 for *T. oliveri* and *T. siamensis*, respectively (Supplementary Table S12), these metrics indicate chromosome-scale assemblies with high per-base accuracy and broad gene-space recovery suitable for comparative and functional genomics. The LAI values (<10) also indicate moderate repeat continuity.

**Table 1.** Assembly statistics of *T. oliveri* and *T. siamensis*

| | ***T. oliveri*** | | ***T. siamensis*** | |
|---|---|---|---|---|
| | **stLFR (pre-scaffold)** | **Hi-C (final)** | **stLFR (pre-scaffold)** | **RagTag (final)** |
| N50 size (bases) | 2,447,080 | 22,175,826 | 319,778 | 19,454,152 |
| L50 number | 86 | 18 | 390 | 23 |
| N75 size (bases) | 407,938 | 15,134,499 | 13,857 | 34,829 |
| L75 number | 313 | 31 | 7,916 | 629 |
| N90 size (bases) | 9,250 | 9,256 | 4,892 | 6,105 |
| L90 number | 5,845 | 4,941 | 29,083 | 15,413 |
| Total (bases) | 990,067,507 | 990,098,900 | 1,138,811,672 | 1,140,691,237 |
| Number of scaffolds | 32,505 | 31,592 | 77,021 | 57,746 |
| Number of scaffolds ≥ 100 kb | 566 | 51 | 964 | 119 |
| Number of scaffolds ≥ 1 Mb | 187 | 38 | 139 | 49 |
| Number of scaffolds ≥ 10 Mb | 10 | 35 | 5 | 36 |
| Longest scaffold (bases) | 15,491,168 | 40,971,832 | 14,091,541 | 40,067,183 |
| GC content (%) | 43.41 | 43.41 | 43.19 | 43.19 |
| BUSCO evaluation (% completeness) | 97.4 | 97.4 | 94.6 | 95.2 |

## Genome annotation

To annotate the *Thyrsostachys* genomes, we employed an integrated pipeline combining ab initio prediction, RNA-seq supported evidence, and protein homology evidence. This analysis revealed distinct differences in the genomic composition of the two bamboo species. *T. oliveri* contained 51,191 predicted gene models, of which 48,070 were protein-coding; *T. siamensis* exhibited 67,483 predicted models with 59,683 protein-coding genes. Average genomic GC content was similar between species (43.41 % in *T. oliveri*, 43.19 % in T. *siamensis*), with exons enriched in GC (53.4 % and 54.7 %, respectively) and introns lower (39.8 % and 39.0 %) (Table 2). Relative to 11 recently published bamboo genomes [27], *Thyrsostachys* shows more compact gene models, with shorter genes and introns overall, while median exon size is broadly conserved; exon number is ~5 in *T. oliveri* and lower in *T. siamensis* (4.27). Full cross-species feature values are provided in Supplementary Table S2.

**Table 2.** Annotation statistics for *T. oliveri* and *T. siamensis*

| | ***T. oliveri*** | ***T. siamensis*** |
|---|---|---|
| Number of predicted gene models | 51,191 | 67483 |
| Total gene length (Mb) | 146.26 | 179.99 |
| Average gene size (nt) | 2857 | 2667 |
| Average number of exons/gene | 5.04 | 4.27 |
| Total exon length (Mb) | 55.98 | 71.55 |
| Average exon length (nt) | 216.8 | 248.2 |

| | | |
|---|---|---|
| GC content of exons (%) | 53.4 | 54.69 |
| Average number of Introns/gene | 4.04 | 3.27 |
| Total intron length (Mb) | 90.33 | 108.51 |
| Average intron length (nt) | 436.4 | 491.5 |
| GC content of introns (%) | 39.81 | 38.99 |

Of these protein-coding genes, 93.9 % in *T. oliveri* and 88.4 % in *T. siamensis* had best hits in the NCBI non-redundant (NR) database. GO terms were assigned to 72.4% of *T. oliveri* genes and 77.8% of *T. siamensis* genes, and KEGG pathways to 32.2% and 28.1%, respectively (Supplementary Table S3). In the GO biological-process category biological-process category, *T. oliveri* was enriched for regulation of DNA-templated transcription, transmembrane transport, and protein ubiquitination, whereas *T. siamensis* showed regulation of DNA-templated transcription, regulation of transcription by RNA polymerase II, and chromatin remodeling. Membrane, nucleus, and cytoplasm dominated the cellular-component category in both species, while ATP binding, metal/zinc-ion binding, and DNA binding dominated the molecular-function category (Supplementary Fig. S8–S9).

For the non-coding RNAs, we also identified 78,048 microRNAs, 1,009 transfer RNAs, 320 ribosomal RNAs and 13,420 small nuclear RNAs in the *T. oliveri* genome (Supplementary Table S4). Similarly, the *T. siamensis* genome contained 36,297 microRNAs, 1,049 transfer RNAs, 336 ribosomal RNAs, and 11,678 small nuclear RNAs (Supplementary Table S5).

**Identification of repetitive elements**

Comparative analysis of repetitive elements in the two *Thyrsostachys* genomes reveals both shared architecture and lineage-specific variation. We grouped repeats into known classes (e.g., LTR, LINE, DNA transposons) and an unclassified category (Other) for sequences lacking clear annotation. Repeats constitute 50.89% of the *T. oliveri* assembly (Table 3) and 48.78% of the *T. siamensis* assembly (Table 4). In *T. oliveri*, unclassified repeats are most abundant (64.55% of all repeats), followed by retrotransposons (24.94%). Within retrotransposons, LTR elements of the Copia and Gypsy superfamilies contribute 13.09% and 9.86% of the genome, respectively. *T. siamensis* exhibits a similar profile: unclassified repeats dominate (67.88%), with Copia and Gypsy elements contributing 13.00% and 8.20% of the genome, respectively. The overall repeat content is approximately two percentage points lower in *T. siamensis*, mainly owing to its smaller Gypsy fraction.

**Table 3.** Repeat elements in the *T. oliveri* genome assembly.

| Types of repeats | Bases (Mb) | % of the assembly | % of total repeats |
|---|---|---|---|
| **DNA transposons:** | 47.68 | 4.81 | 9.46 |
| **Retrotransposons:** | | | |
| LINE | 8.47 | 0.85 | 1.68 |
| SINE | 0.09 | 0.00 | 0.00 |
| LTR: *Copia* | 65.94 | 6.66 | 13.09 |
| LTR: *Gypsy* | 49.68 | 5.02 | 9.86 |
| LTR: Others | 1.56 | 0.16 | 0.31 |

| | | | |
|---|---|---|---|
| **Simple sequence repeats:** | 5.31 | 0.54 | 1.05 |
| **Others:** | 325.08 | 32.85 | 64.55 |
| **Total** | 503.81 | 50.89 | |

**Table 4.** Repeat elements in the *T. siamesis* genome assembly.

| Types of repeats | Bases (Mb) | % of the assembly | % of total repeats |
|---|---|---|---|
| **DNA transposons:** | 45.77 | 4.01 | 8.22 |
| **Retrotransposons:** | | | |
| LINE | 9.09 | 0.80 | 1.63 |
| SINE | 0.00 | 0.00 | 0.00 |
| LTR: *Copia* | 72.08 | 6.32 | 13.00 |
| LTR: *Gypsy* | 45.62 | 4.00 | 8.20 |
| LTR: Others | 0.31 | 0.03 | 0.05 |
| **Simple sequence repeats:** | 5.70 | 0.50 | 1.02 |
| **Others:** | 377.90 | 33.12 | 67.88 |
| **Total** | 556.47 | 48.78 | |

**Phylogenetic and comparative genomics analyses**

To estimate the relative timing of divergence and WGD events in the *Thyrsostachys* lineage, we quantified four-fold degenerate transversions (4DTv) for

orthologous and paralogous gene pairs and synonymous substitution rates (Ks) for syntenic anchor pairs. The interspecific distances are concordant across metrics, with the closest affinity between *T. oliveri* and *T. siamensis* (4DTv = 0.0164; Ks = 0.0131), followed by *D. sinicus* (0.0178; 0.0375), *B. amplexicaulis* (0.0180; 0.0416), *P. edulis* (0.0516; 0.135), and *O. latifolia* (0.0810; 0.2184) (Fig. 2A and Supplementary Fig. S10). Within genomes, 4DTv distributions of paralogous pairs (n = 28,430 in *T. oliveri*; n = 28,061 in *T. siamensis*) are bimodal, with peaks at 0.056 and 0.208 in *T. oliveri* and 0.059 and 0.231 in *T. siamensis*; the corresponding Ks distributions show peaks at 0.150 and 0.516 in *T. oliveri* and 0.152 and 0.533 in *T. siamensis*. Concordant 4DTv and Ks modes support a relatively recent WGD shared by the two *Thyrsostachys* species.

Next, we reconstructed a maximum-likelihood phylogeny from 605 single-copy orthologues sampled from *Thyrsostachys* and eight reference grasses (*B. amplexicaulis*, *P. edulis*, *D. latiflorus*, *D. sinicus*, *G. angustifolia*, *R. guianensis*, *B. distachyon*, and *O. sativa*) (Fig. 2B). The two *Thyrsostachys* species form a clade sister to the *Dendrocalamus* pair, with a crown age of 5.39 million years ago (Mya) and a stem divergence from *Dendrocalamus* at 10.55 Ma

To test whether the *Thyrsostachys* A, B and C subgenomes correspond to the core A, B and C subgenomes in other hexaploid bamboos, we inferred orthogroups and gene trees with OrthoFinder using proteomes from *Thyrsostachys* (this study) and three hexaploids (*M. baccifera*, *B. amplexicaulis*, *D. sinicus*). For single-copy orthogroups with representation across species, we labeled each sequence by its subgenome and assessed subgenome-specific clustering. Across gene trees, A copies from different species cluster together, and likewise for B and C, indicating that

the *Thyrsostachys* subgenomes are orthologous to the core A, B and C subgenomes described for other hexaploids. (Supplementary Fig. S11).

Orthogroup clustering placed 436,476 of 469,186 proteins (93 %) into 37,153 families. Within this 11-species framework, a core set of 12,894 families is shared by all 11 species (Fig. 2C), while species-unique families number 319 in *T. oliveri*, enriched for root-development and plasmodesmata genes, and 1,204 in *T. siamensis*, highlighting histone-binding and proteasome pathways (Supplementary Tables S6–S7).

Finally, gene-copy profiling revealed that hexaploid *D. latiflorus* has undergone the greatest expansion of gene families, followed by *T. siamensis*, with *T. oliveri* showing fewer duplications (Fig. 2D). In *T. oliveri*, 313 families expanded and 637 contracted: in *T. siamensis*, 605 expanded and 354 contracted. GO enrichment of *T. oliveri* expansions highlights protein-kinase activity, defense response, and monooxygenase activity (Supplementary Fig. S12-S13), whereas *T. siamensis* expansions are enriched for ADP-binding, carbohydrate-binding, and DNA-integration functions (Supplementary Fig. S14-S15). At the *Thyrsostachys* crown node, CAFE identifies significant shifts in gene family size, with 197 expansions and 268 contractions. Contracted families are enriched for cell-surface receptor protein Ser/Thr-kinase signaling and broader defense/interaction processes (e.g., defense responses to bacteria and oomycetes), together with oxidoreductase functions and polyamine biosynthesis (adenosylmethionine decarboxylase, spermidine/spermine biosynthetic process). A plasma-membrane cellular-component signal among contractions is compatible with reductions in some membrane-localized receptor-like kinase repertoires (Supplementary Table S8). In contrast, expanded families at this ancestral node are enriched for stress/interaction responses (notably defense agianst

other organisms), osmolyte transport (L-proline/proline transport), proteostasis and organelle-processing functions (proteasome subcomplex, mitochondrial inner-membrane peptidase), and ADP/phosphate binding and transport. Collectively, these patterns indicate a lineage-level remodeling of stress and signaling-related gene content at the *Thyrsostachys* crown node (Supplementary Table S8).

**Homoeolog expression patterns**

From RNA-seq of leaf and root (three biological replicates each), we identified 2,197 homoeologous triads in *T. oliveri* and 2,054 triads in *T. siamensis*. Triads in each tissue with a combined expression of A, B, and C homoeolog expression greater than 0.5 TPM were designated “expressed” and classified into six relative-expression categories (Fig. 3 and Supplementary Table S9). Balanced expression was observed in 36.8 % of expressed *T. oliveri* triads and 33.8% of expressed *T. siamensis* triads, whereas single-homoeolog dominance was the least common (23.4% and 24.6%, respectively). Single-homoeolog suppression affected 39.9% of expressed *T. oliveri* triads and 41.8% of expressed *T. siamensis* triads. Within the dominance categories, B-homoeolog dominance was rare (7.3% in *T. oliveri*; 7.7% in *T. siamensis*), while A- and C-homoeolog dominance showed slightly higher frequencies. Among the suppression categories, B-homoeolog suppression was most frequent in *T. oliveri* (14.5%), whereas C-homoeolog suppression led in *T. siamensis* (14.6%) (Supplementary Table S9).

**Functional enrichment of homoeolog bias categories**

We performed GO enrichment across six bias categories in leaf and root tissues of *T. oliveri* and *T. siamensis* (Supplementary Table S10); five modules recurred: (i) Photosynthesis/growth: C-dominant leaves are enriched for chlorophyll and

gibberellin/terpenoid biosynthesis, indicating a disproportionately large contribution from subgenome C to photosynthesis and growth in leaves. (ii) Cell wall/carbohydrate flux: In *T. oliveri*, B-dominant sets emphasize GDP-mannose supply and cell-wall organization; in *T. siamensis*, they highlight broad transmembrane transport and symporter activity, consistent with roles in carbon allocation. (iii) Hormone and redox signaling: Auxin/brassinosteroid and oxidative-stress detoxification terms concentrate in A- and B- suppressed sets, supporting hormone–ROS crosstalk under stress. (iv) Membrane trafficking/organelles: A- and B-suppressed sets are enriched for ESCRT/HOPS, vacuole organization, and nuclear pore/transport, indicating reduced A/B contribution to these pathways and partitioning across subgenomes rather than pathway absence. (v) Organelle metabolism: Respiration and chloroplast compartment terms co-occur across subgenomes, indicating coordinated mitochondrial–chloroplast support. Together, these five modules are consistent with subgenome C preferentially supporting leaf photosynthesis and growth, subgenome B being associated with cell-wall biogenesis and carbon allocation, and subgenome A contributing to terpenoid/sterol metabolism and translational/quality control. Endomembrane remodeling signals are prominent. To demonstrate subgenome-specific expression biases that differ between species, we highlight a mannose-6-phosphate isomerase (PMI; GO:0004476) triad that is B-dominant in *T. oliveri* leaves but not B-dominant in *T. siamensis* leaves, illustrating species-specific subgenome bias. Full term lists and statistics are provided in Supplementary Table S10**.**

**Subgenome composition and gene retention in *Thyrsostachys*.**

To place the expression results in structural context, we quantified gene retention across the A, B, and C subgenomes by classifying gene sets into triads (1:1:1

across A/B/C), duplets (present in exactly two subgenomes), singletons (present in one), and other multi-copy configurations (including CNVs; any class in which a present subgenome contributes >1 copy) (Supplementary Table S11).

In *T. oliveri*, subgenomes A, B, and C contain 15,803, 14,691, and 13,627 genes, with 2,197 genes per subgenome in 1:1:1 triads (13.90%, 14.95%, 16.12%). Among non-triad classes, duplets account for 33.80%, 32.82%, and 31.84%, singletons for 3.46%, 2.55%, and 1.74%, and the remaining genes fall into other multi-copy (incl. CNVs) at 48.85%, 49.68%, and 50.30% for A, B, and C, respectively.

In *T. siamensis*, subgenomes A, B, and C contain 17,670, 16,171, and 14,838 genes; 2,054 genes per subgenome are in 1:1:1 triads (11.62%, 12.70%, 13.84%). Duplets comprise 31.77%, 29.97%, and 29.72%, singletons 4.24%, 3.36%, and 2.47%, and other multi-copy (incl. CNVs) 52.37%, 53.97%, and 53.97% for A, B, and C, respectively.

Across both species, subgenome C consistently shows the highest triad retention and the lowest absence-bearing fraction (duplet + singleton; 33.58% in *T. oliveri*, 32.19% in *T. siamensis*), whereas subgenome A shows the greatest loss (37.26% and 36.01%). These complementary patterns of loss and duplication indicate asymmetric fractionation among subgenomes following polyploidization (Supplementary Table S11).

## Discussion

Bamboo is one of the world’s most important non-timber forest resources and a crucial component of forest ecosystems. Despite its significance, only a limited number of bamboo genomes have been sequenced to date, resulting in limited

knowledge of bamboo biological mechanisms and hindering progress in understanding genome evolution and the potential for molecular breeding. In this study, we sequenced and assembled chromosome-level genomes of two bamboo species, *T. oliveri* and *T. siamensis*, which resulted in the first reference genome for the *Thyrsostachys* genus. The assembled genome sizes of *T. oliveri* and *T. siamensis* were 990.1 Mb (N50 = 22.18 Mb) and 1.14 Gb (N50 = 19.45 Mb), respectively. The assemblies were anchored into 35 pseudochromosomes, and subgenome partitioning identified three distinct subgenomes, supporting a hexaploid structure (2n = 6x = 70). Compared with previously reported hexaploid bamboo genome assemblies, the genome sizes of *T. oliveri* and *T. siamensis* are smaller than those of *D. latiflorus* (1,368 Mb, 1C) [22] and *D. brandisii* (1,378 Mb, 1C)[21], but larger than that of *B. amplexicaulis* (848 Mb) [9]. Based on the BUSCO assessment, the completeness of the gene space in the *T. oliveri* and *T. siamensis* genomes was estimated at 97.4% and 95.2%, respectively, indicating that the current assemblies cover most of their genomes. The assembly size of *T. oliveri* is slightly smaller than the estimated genome size based on both k-mer analysis and flow cytometry (1.157 Gb). This slight difference likely reflects unassembled repetitive portions of the genome. In contrast, the assembly size of *T. siamensis* is close to the estimates from both methods (1.172 Gb). Compared with PacBio HiFi references, our stLFR+Hi-C assemblies achieve chromosome-scale, scaffold N50s and comparable BUSCO completeness (Supplementary Table S12) [22, 26, 85], while exhibiting lower repeat continuity (LAI) and base-level QV, as expected for short-read barcoded libraries. These trade-offs primarily affect long repetitive regions and do not preclude the gene-space and comparative analyses reported here. While Merqury QV indicates very high per-base accuracy, our LAI scores (<10) point to moderate repeat continuity, consistent with

assemblies derived from 150-bp stLFR short reads. Long LTR retrotransposons and other complex or highly similar repeats are likely fragmented or collapsed, and some homoeologous regions in this hexaploid context may remain under-resolved. Future long-read (e.g., HiFi/ONT) and T2T scaffolding would further improve structural completeness and repeat resolution.

The genome annotations of *T. oliveri* and *T. siamensis* contained 51,191 and 67,483 predicted gene models, respectively. Compared with other bamboo genomes, the number of annotated genes in the haploid assembly of *T. oliveri* is similar to that reported for *P. edulis* (51,074) [13] and higher than that of *B. amplexicaulis* (47,056) [9]. In contrast, the 67,483 genes in the haploid *T. siamensis* assembly correspond to roughly half the total counts observed in diploid-level assemblies: 135,231 genes for *D. latiflorus* [22] and 126,817 genes for *D. brandisii* [21], both of which were assembled at 2n = 70. The proportions of repetitive sequences identified in our genome assemblies were 50.89% for *T. oliveri* and 48.78% for *T. siamensis*. The composition of repeat types was highly similar between the two species, which is unsurprising given their close evolutionary relationship. The content of repetitive elements in our genome assemblies is slightly lower than that reported for moso bamboo [13, 23], *D. latiflorus* [22], and *D. brandisii* [21]. LTR elements are the predominant retrotransposon in our bamboo assemblies. Consistent with previous studies, LTR retroelements are also the most common elements in bamboo genomes [13, 21, 22]. Phylogenetic analysis using single-copy orthologs placed *T. oliveri* and *T. siamensis* as sister taxa, diverging approximately 5.39 Mya. Consistent signals from 4DTv and Ks analyses (using syntenic anchors) indicate that a recent, shared whole-genome duplication (WGD) event occurred in their common ancestor before this speciation.

Gene family analysis revealed distinct genomic features contributing to the phenotypic specificity and adaptive divergence of *T. oliveri* and *T. siamensis*. While 12,894 gene families were shared across 11 species, *T. oliveri* and *T. siamensis* possessed 319 and 1,204 species-specific gene families, respectively, with functions potentially related to root development and carbon metabolism in *T. oliveri* and protein degradation in *T. siamensis*. In our analysis, *T. oliveri* was found to have fewer expanded gene families and more contracted gene families compared to *T. siamensis*. In contrast, *T. siamensis* exhibited a greater number of expanded gene families and fewer contractions. Given that *T. siamensis* also had a higher overall gene count, it is likely that the expanded families in this species contain more gained genes than were lost through contraction, potentially contributing to its distinct genomic and phenotypic features such as rapid growth and high shoot productivity.

By examining the expanded gene families in *T. oliveri*, we found enrichment in genes associated with protein kinase activity and monooxygenase activity that may be linked to signaling pathways and metabolic regulation under more specialized ecological conditions. In *T. siamensis*, expanded gene families were enriched in functions related to ADP binding and protein dimerization activity that potentially support its rapid growth, high shoot productivity, and broader environmental adaptability relative to *T. oliveri* and other woody bamboos. When compared with other woody bamboos, *P. edulis* shows expansions in gene families associated with lignin biosynthesis and has the highest copy numbers in the peroxidase gene family [13]. In contrast, *D. latiflorus* exhibits expansions in genes involved in telomere maintenance and DNA repair, which likely play important roles in supporting its long-lived vegetative growth [22]. These species-specific expansions and contractions of gene families therefore provide insights into the phenotypic characteristics, adaptive evolution, and

unique evolutionary pressures that have shaped the genomes of *Thyrsostachys* species compared with other bamboo lineages. At the genus level, our gene family analysis revealed that the contraction of receptor-like kinase, pathogen-response, and polyamine biosynthesis families suggests streamlining of certain signaling and defense pathways, whereas expansion of stress-response, osmolyte transport, and proteostasis families indicates adaptive innovations that enhance cellular homeostasis.

Allopolyploidy is a defining feature of many bamboo species. Previous studies have shown that duplicated gene pairs may display homoeolog expression bias in several allopolyploid species [86-90], in which bias refers to the preferential expression of one homoeolog relative to the other [91]. In our hexaploid *Thyrsostachys* species, single-homoeolog suppression was the most frequent expression category, affecting 39.9% of expressed triads in *T. oliveri* and 41.8% in *T. siamensis*, a pattern also observed in other woody bamboos [27]. Balanced expression accounted for 36.8% of triads in *T. oliveri* and 33.8% in *T. siamensis*, consistent with coordinated subgenome regulation observed in hexaploid bamboo species [27]. Single-homoeolog dominance was relatively uncommon (<25%). Among the dominant triads, the B-subgenome was the least frequently dominant, while A- and C-subgenomes showed comparable but slightly higher levels of dominance. These findings are consistent with previous reports in *M. baccifera*, *B. amplexicaulis* and *D. sinicus* bamboo species [27]. Suppression biases differed between species: B-subgenome suppression predominated in *T. oliveri*, whereas C-subgenome suppression was more frequent in *T. siamensis*, suggesting lineage-specific regulatory divergence. Together, these findings indicate that, while balanced expression remains substantial, single-homoeolog suppression is

a major driver of subgenome-specific expression and may underlie functional differentiation in these two *Thyrsostachys* species.

## Data Availability

Both *T. oliveri* and *T. siamensis* genome assemblies have been deposited at NCBI under accession numbers JAWCWW000000000 and JBEFOJ000000000, respectively. The raw stLFR reads for *T. oliveri* and *T. siamensis* are available in the NCBI SRA database under accession numbers SRR32915794 and SRR32915889, respectively. The transcriptome data for *T. oliveri* were submitted under SRR33015685 and SRR33015684. The transcriptome data for *T. siamensis* were submitted under SRR33015804 and SRR33015803. All additional supporting data are available in the *GigaScience* repository, GigaDB [92].

## Abbreviations

Hi-C: chromosome conformation capture; stLFR: single-tube long-fragment read ;4DTv: four-fold degenerate transversion; BLASTP: Basic Local Alignment Search Tool for protein; bp: base pair; BUSCO: Benchmarking Universal Single-Copy Orthologues; EVM: EvidenceModeler; GO: Gene Ontology; kb: kilobase; Gb: gigabase; KEGG: Kyoto Encyclopedia of Genes and Genomes; LTR: long terminal repeat; Mb: megabase; Mya: million years ago;

## Additional files

**Supplementary Table S1.** Summary of sequencing data from stLFR and Hi-C platforms.

**Supplementary Table S2.** Comparative gene features across *Thyrsostachys* and 11 bamboo genomes.

**Supplementary Table S3.** Functional annotations of *T. oliveri* and *T. siamensis* protein-coding genes.

**Supplementary Table S4.** Noncoding RNA in the *T. oliveri* genome.

**Supplementary Table S5.** Noncoding RNA in the *T. siamensis* genome.

**Supplementary Table S6.** GO term enrichment analyses for gene families specific to *T. oliveri.*

**Supplementary Table S7.** GO term enrichment analyses for gene families specific to *T. siamensis.*

**Supplementary Table S8.** GO term enrichment analyses for gene families specific to *Thyrsostachys* crown node.

**Supplementary Table S9.** Percentage of syntenic triads assigned to the six homoeolog expression bias categories.

**Supplementary Table S10.** GO enrichment of homoeolog expression-bias categories in leaf and root of two *Thyrsostachys* bamboos.

**Supplementary Table S11.** Subgenome composition and distribution of 1:1:1 triads, duplets, and singletons in *Thyrsostachys*.

**Supplementary Table S12.** Assembly technology and key metrics across *Thyrsostachys* and PacBio long-read bamboo references.

**Supplementary Fig. S1** *Thyrsostachys oliveri* morphology

**Supplementary Fig. S2** *Thyrsostachys siamensis* morphology.

**Supplementary Fig. S3** Example of manual Hi-C curation performed by Biomarker Technologies (BMK).

**Supplementary Fig. S4** Collinearity between *Thyrsostachys* genomes and *D. sinicus*

**Supplementary Fig. S5** Flow-cytometric estimation of nuclear DNA content in Thyrsostachys species.

**Supplementary Fig. S6** Genome-survey k-mer distributions for hexaploidy Thyrsostachys.

**Supplementary Fig. S7** Genome-wide Hi-C contact map of hexaploid *Thyrsostachys oliveri*.

**Supplementary Fig. S8** GO annotation statistics for *T. oliveri*.

**Supplementary Fig. S9** GO annotation statistics for *T. siamensis*.

**Supplementary Fig. S10** Density plot showing pairwise synonymous substitution rate (Ks) values between the indicated pairs of genomes.

**Supplementary Fig. S11** Subgenome-specific clustering of single-copy orthologs across hexaploid bamboos

**Supplementary Fig. S12** GO term enrichment analyses for expanded gene families in *T. oliveri.*

**Supplementary Fig. S13** GO term enrichment analyses for contracted gene families in *T. oliveri.*

**Supplementary Fig. S14** GO term enrichment analyses for expanded gene families in *T. siamensis.*

**Supplementary Fig. S15** GO term enrichment analyses for contracted gene families in *T. siamensis*.

**Acknowledgements**

We thank Dr. Atchara Teerawatananon, Natural History Museum, National Science Museum, Technopolis, Pathum Thani 12120, Thailand, for kindly providing the bamboo photographs used in this study.

**Funding**

This work was supported by the National Science and Technology Development Agency (NSTDA), grant number P2251271.

**Competing Interests**

The authors declare that they have no competing interests.

**Author Contributions**

Research study was designed by C.N., S.K., W.P. and S.T. Sample collection and laboratory work (DNA/RNA extraction, sequencing library preparation, flow cytometry) were performed by S.K., S.U-t., D.S., P.W., and S.S. Bioinformatics analyses were performed by C.N., P.P., and C.S. The manuscript was written and revised by C.N., S.K. and W.P. All authors read and approved the final manuscript.

**Reference**

1. Zhang H, Zhuang S, Sun B, Ji H, Li C and Zhou S. Estimation of biomass and carbon storage of moso bamboo (Phyllostachys pubescens Mazel ex Houz.)

in southern China using a diameter–age bivariate distribution model. Forestry: An International Journal of Forest Research. 2014;87 5:674-82. doi:10.1093/forestry/cpu028.

2. Jember AA, Taye MA, Gebeyehu G, Mulu G, Long TT, Jayaraman D and Abebe S. Carbon stock potential of highland bamboo plantations in northwestern Ethiopia. Carbon Balance and Management. 2023;18 1:3. doi:10.1186/s13021-023-00224-2.
3. Basak M, Dutta S, Biswas S, Chakraborty S, Sarkar A, Rahaman T, et al. Genomic insights into growth and development of bamboos: what have we learnt and what more to discover? Trees. 2021;35 6:1771-91. doi:10.1007/s00468-021-02197-6.
4. Du H, Mao F, Li X, Zhou G, Xu X, Han N, et al. Mapping Global Bamboo Forest Distribution Using Multisource Remote Sensing Data. IEEE Journal of Selected Topics in Applied Earth Observations and Remote Sensing. 2018;11 5:1458-71. doi:10.1109/JSTARS.2018.2800127.
5. Food and Agriculture Organization of the United N. Putting bamboo on the map. 2021.
6. Soreng RJ, Peterson PM, Romaschenko K, Davidse G, Teisher JK, Clark LG, et al. A worldwide phylogenetic classification of the Poaceae (Gramineae) II: An update and a comparison of two 2015 classifications. Journal of Systematics and Evolution. 2017;55 4:259-90. doi:https://doi.org/10.1111/jse.12262.
7. Akinlabi ET, Anane-Fenin K and Akwada DR. Bamboo Taxonomy and Distribution Across the Globe. In: Akinlabi ET, Anane-Fenin K and Akwada DR, editors. Bamboo: The Multipurpose Plant. Cham: Springer International Publishing; 2017. p. 1-37.
8. Zhou M, Xu C, Shen L, Xiang W and Tang D. Evolution of genome sizes in Chinese Bambusoideae (Poaceae) in relation to karyotype. Trees. 2017;31 1:41-8. doi:10.1007/s00468-016-1453-y.
9. Guo Z-H, Ma P-F, Yang G-Q, Hu J-Y, Liu Y-L, Xia E-H, et al. Genome Sequences Provide Insights into the Reticulate Origin and Unique Traits of Woody Bamboos. Molecular Plant. 2019;12 10:1353-65. doi:https://doi.org/10.1016/j.molp.2019.05.009.
10. Clark L, Londoño X and Ruiz-Sanchez E. Bamboo taxonomy and habitat. Bamboo: The plant and its uses. 2015:1-30.
11. Group BP. An updated tribal and subtribal classification of the bamboos (Poaceae: Bambusoideae). The Journal of the American Bamboo Society. 2012:1.
12. Zhao H, Zhao S, Bamboo INf, Rattan, Fei B, Liu H, et al. Announcing the Genome Atlas of Bamboo and Rattan (GABR) project: promoting research in evolution and in economically and ecologically beneficial plants. GigaScience. 2017;6 7:gix046.
13. Zhao H, Gao Z, Wang L, Wang J, Wang S, Fei B, et al. Chromosome-level reference genome and alternative splicing atlas of moso bamboo (Phyllostachys edulis). GigaScience. 2018;7 10 doi:10.1093/gigascience/giy115.
14. Schröder S. Thyrsostachys siamensis – Monastery Bamboo. 2020.
15. Sae-Long W, Chompoorat T, Limkatanyu S, Hansapinyo C, Buakla A, Sukontasukkul P, et al. Investigation on the tensile strength of Dendrocalamus

sericeus, Phyllostachys makinoi, and Thyrsostachys oliveri bamboo: Experiment and simulations. Case Studies in Construction Materials. 2024;20:e03205. doi:https://doi.org/10.1016/j.cscm.2024.e03205.
16. Marueng V, Tanawat T, Pakdeelun W and Klung-ngoen W. *Bamboo Value Chain Analysis in Thailand*. 2021-09 2021. Bangkok: Thailand Environment Institute.
17. Chaowana K, Wisadsatorn S and Chaowana P. Bamboo as a sustainable building material—culm characteristics and properties. Sustainability. 2021;13 13:7376.
18. Zhang Z, Rao F and Wang Y. Morphological, Chemical, and Physical–Mechanical Properties of a Clumping Bamboo (Thyrsostachys oliveri) for Construction Applications. Polymers. 2022;14 17:3681.
19. Banik RL. Thyrsostachys Gamble. Silviculture of South Asian Priority Bamboos. Singapore: Springer Singapore; 2016. p. 261-76.
20. Obsuwan K, Duangmanee A and Thepsithar C. In vitro propagation of a useful tropical bamboo, Thyrsostachys siamensis Gamble, through shoot-derived callus. Horticulture, Environment, and Biotechnology. 2019;60 2:261-7. doi:10.1007/s13580-018-00119-z.
21. Jiang J, Zhang Z, Bai Y, Wang X, Dou Y, Geng R, et al. Chromosomal-level genome and metabolome analyses of highly heterozygous allohexaploid Dendrocalamus brandisii elucidate shoot quality and developmental characteristics. J Integr Plant Biol. 2024;66 6:1087-105. doi:10.1111/jipb.13592.
22. Zheng Y, Yang D, Rong J, Liguang C, Zhu Q, He T, et al. Allele-aware chromosome-scale assembly of the allopolyploid genome of hexaploid Ma bamboo (Dendrocalamus latiflorus Munro). Journal of Integrative Plant Biology. 2022;64 doi:10.1111/jipb.13217.
23. Peng Z, Lu Y, Li L, Zhao Q, Feng Q, Gao Z, et al. The draft genome of the fast-growing non-timber forest species moso bamboo (Phyllostachys heterocycla). Nature Genetics. 2013;45 4:456-61. doi:10.1038/ng.2569.
24. Wang Y-J, Guo C, Zhao L, Mao L, Hu X-Z, Yang Y-Z, et al. Haplotype-resolved nonaploid genome provides insights into in vitro flowering in bamboos. Horticulture Research. 2024;11 12 doi:10.1093/hr/uhae250.
25. Li W, Shi C, Li K, Zhang QJ, Tong Y, Zhang Y, et al. Draft genome of the herbaceous bamboo Raddia distichophylla. G3 (Bethesda). 2021;11 2 doi:10.1093/g3journal/jkaa049.
26. Wang H, Ding H, Cheng P, Jin X, Fu Y and Peng Y. Chromosome-level genome assembly and annotation of Phyllostachys violascens ‘Prevernalis’. Scientific Data. 2025;12 1:912. doi:10.1038/s41597-025-04556-1.
27. Ma PF, Liu YL, Guo C, Jin G, Guo ZH, Mao L, et al. Genome assemblies of 11 bamboo species highlight diversification induced by dynamic subgenome dominance. Nat Genet. 2024;56 4:710-20. doi:10.1038/s41588-024-01683-0.
28. Wang O, Chin R, Cheng X, Wu MKY, Mao Q, Tang J, et al. Efficient and unique cobarcoding of second-generation sequencing reads from long DNA molecules enabling cost-effective and accurate sequencing, haplotyping, and de novo assembly. Genome research. 2019;29 5:798-808.
29. Pootakham W, Sonthirod C, Naktang C, Yundaeng C, Yoocha T, Kongkachana W, et al. Genome assemblies of Vigna reflexo-pilosa (créole bean) and its progenitors, Vigna hirtella and Vigna trinervia, revealed

homoeolog expression bias and expression-level dominance in the allotetraploid. GigaScience. 2023;12:giad050. doi:10.1093/gigascience/giad050.

30. Xie T, Zheng J-F, Liu S, Peng C, Zhou Y-M, Yang Q-Y and Zhang H-Y. De Novo Plant Genome Assembly Based on Chromatin Interactions: A Case Study of Arabidopsis thaliana. Molecular Plant. 2015;8 3:489-92. doi:https://doi.org/10.1016/j.molp.2014.12.015.
31. BGI-biotools: stLFRdenovo v1.0.5. https://github.com/BGI-biotools/stLFRdenovo/releases/tag/v1.0.5 (2025). Accessed 2025-03-01.
32. Putnam NH, O'Connell BL, Stites JC, Rice BJ, Blanchette M, Calef R, et al. Chromosome-scale shotgun assembly using an in vitro method for long-range linkage. Genome Res. 2016;26 3:342-50. doi:10.1101/gr.193474.115.
33. Li H and Durbin R. Fast and accurate short read alignment with Burrows–Wheeler transform. Bioinformatics. 2009;25 14:1754-60. doi:10.1093/bioinformatics/btp324.
34. Servant N, Varoquaux N, Lajoie BR, Viara E, Chen C-J, Vert J-P, et al. HiC-Pro: an optimized and flexible pipeline for Hi-C data processing. Genome Biology. 2015;16 1:259. doi:10.1186/s13059-015-0831-x.
35. Burton JN, Adey A, Patwardhan RP, Qiu R, Kitzman JO and Shendure J. Chromosome-scale scaffolding of de novo genome assemblies based on chromatin interactions. Nature Biotechnology. 2013;31 12:1119-25. doi:10.1038/nbt.2727.
36. Alonge M, Soyk S, Ramakrishnan S, Wang X, Goodwin S, Sedlazeck FJ, et al. RaGOO: fast and accurate reference-guided scaffolding of draft genomes. Genome Biology. 2019;20 1:224. doi:10.1186/s13059-019-1829-6.
37. RagTag developers: RagTag software v1.1.0. https://github.com/malonge/RagTag (2025). Accessed 2025-02-07.
38. Vurture GW, Sedlazeck FJ, Nattestad M, Underwood CJ, Fang H, Gurtowski J and Schatz MC. GenomeScope: fast reference-free genome profiling from short reads. Bioinformatics. 2017;33 14:2202-4. doi:10.1093/bioinformatics/btx153.
39. GenomeScope developers: GenomeScope v2.0. http://genomescope.org/genomescope2.0 (2025). Accessed 2025-03-18.
40. DoleŽEl J and BartoŠ JAN. Plant DNA Flow Cytometry and Estimation of Nuclear Genome Size. Annals of Botany. 2005;95 1:99-110. doi:10.1093/aob/mci005.
41. Kim D, Paggi JM, Park C, Bennett C and Salzberg SL. Graph-based genome alignment and genotyping with HISAT2 and HISAT-genotype. Nature Biotechnology. 2019;37 8:907-15. doi:10.1038/s41587-019-0201-4.
42. Manni M, Berkeley MR, Seppey M, Simão FA and Zdobnov EM. BUSCO Update: Novel and Streamlined Workflows along with Broader and Deeper Phylogenetic Coverage for Scoring of Eukaryotic, Prokaryotic, and Viral Genomes. Molecular Biology and Evolution. 2021;38 10:4647-54. doi:10.1093/molbev/msab199.
43. Kriventseva EV, Tegenfeldt F, Petty TJ, Waterhouse RM, Simão FA, Pozdnyakov IA, et al. OrthoDB v8: update of the hierarchical catalog of orthologs and the underlying free software. Nucleic Acids Research. 2015;43 D1:D250-D6. doi:10.1093/nar/gku1220.

44. Ou S and Jiang N. LTR_retriever: A Highly Accurate and Sensitive Program for Identification of Long Terminal Repeat Retrotransposons. Plant Physiol. 2018;176 2:1410-22. doi:10.1104/pp.17.01310.
45. Rhie A, Walenz BP, Koren S and Phillippy AM. Merqury: reference-free quality, completeness, and phasing assessment for genome assemblies. Genome Biology. 2020;21 1:245. doi:10.1186/s13059-020-02134-9.
46. Flynn JM, Hubley R, Goubert C, Rosen J, Clark AG, Feschotte C and Smit AF. RepeatModeler2 for automated genomic discovery of transposable element families. Proceedings of the National Academy of Sciences. 2020;117 17:9451-7. doi:10.1073/pnas.1921046117.
47. Tempel S. Using and Understanding RepeatMasker. In: Bigot Y, editor. Mobile Genetic Elements: Protocols and Genomic Applications. Totowa, NJ: Humana Press; 2012. p. 29-51.
48. Jurka J, Kapitonov VV, Pavlicek A, Klonowski P, Kohany O and Walichiewicz J. Repbase Update, a database of eukaryotic repetitive elements. Cytogenetic and Genome Research. 2005;110 1-4:462-7. doi:10.1159/000084979.
49. Haas BJ, Salzberg SL, Zhu W, Pertea M, Allen JE, Orvis J, et al. Automated eukaryotic gene structure annotation using EVidenceModeler and the Program to Assemble Spliced Alignments. Genome Biology. 2008;9 1:R7. doi:10.1186/gb-2008-9-1-r7.
50. Holt C and Yandell M. MAKER2: an annotation pipeline and genome-database management tool for second-generation genome projects. BMC Bioinformatics. 2011;12 1:491. doi:10.1186/1471-2105-12-491.
51. Haas BJ, Papanicolaou A, Yassour M, Grabherr M, Blood PD, Bowden J, et al. De novo transcript sequence reconstruction from RNA-seq using the Trinity platform for reference generation and analysis. Nature Protocols. 2013;8 8:1494-512. doi:10.1038/nprot.2013.084.
52. Fu L, Niu B, Zhu Z, Wu S and Li W. CD-HIT: accelerated for clustering the next-generation sequencing data. Bioinformatics. 2012;28 23:3150-2. doi:10.1093/bioinformatics/bts565.
53. Li W and Godzik A. Cd-hit: a fast program for clustering and comparing large sets of protein or nucleotide sequences. Bioinformatics. 2006;22 13:1658-9. doi:10.1093/bioinformatics/btl158.
54. Haas BJ, Delcher AL, Mount SM, Wortman JR, Smith Jr RK, Hannick LI, et al. Improving the Arabidopsis genome annotation using maximal transcript alignment assemblies. Nucleic Acids Research. 2003;31 19:5654-66. doi:10.1093/nar/gkg770.
55. Wu TD and Watanabe CK. GMAP: a genomic mapping and alignment program for mRNA and EST sequences. Bioinformatics. 2005;21 9:1859-75. doi:10.1093/bioinformatics/bti310.
56. Dendrocalamus genome project team. Dendrocalamus latiflorus genome dataset. 2024. https://doi.org/10.6084/m9.figshare.24411913.v3.
57. Dendrocalamus sinicus, Bonia amplexicaulis, Guadua angustifolia, Olyra latifolia, and Raddia guianensis dataset. https://genomevolution.org/coge.
58. Huang X, Adams MD, Zhou H and Kerlavage AR. A Tool for Analyzing and Annotating Genomic Sequences. Genomics. 1997;46 1:37-45. doi:https://doi.org/10.1006/geno.1997.4984.

59. Stanke M, Steinkamp R, Waack S and Morgenstern B. AUGUSTUS: a web server for gene finding in eukaryotes. Nucleic Acids Research. 2004;32 suppl_2:W309-W12. doi:10.1093/nar/gkh379.
60. Korf I. Gene finding in novel genomes. BMC Bioinformatics. 2004;5 1:59. doi:10.1186/1471-2105-5-59.
61. BioBam B: OmicsBox v2.0.10. https://www.biobam.com/download-omicsbox (2025). Accessed 2025-03-20.
62. Gish W and States DJ. Identification of protein coding regions by database similarity search. Nature Genetics. 1993;3 3:266-72. doi:10.1038/ng0393-266.
63. Chan Patricia P, Lin Brian Y, Mak Allysia J and Lowe Todd M. tRNAscan-SE 2.0: improved detection and functional classification of transfer RNA genes. Nucleic Acids Research. 2021;49 16:9077-96. doi:10.1093/nar/gkab688.
64. Griffiths-Jones S, Moxon S, Marshall M, Khanna A, Eddy SR and Bateman A. Rfam: annotating non-coding RNAs in complete genomes. Nucleic Acids Research. 2005;33 suppl_1:D121-D4. doi:10.1093/nar/gki081.
65. Nawrocki EP and Eddy SR. Infernal 1.1: 100-fold faster RNA homology searches. Bioinformatics. 2013;29 22:2933-5. doi:10.1093/bioinformatics/btt509.
66. Emms DM and Kelly S. OrthoFinder: phylogenetic orthology inference for comparative genomics. Genome Biology. 2019;20 1:238. doi:10.1186/s13059-019-1832-y.
67. Edgar RC. MUSCLE: a multiple sequence alignment method with reduced time and space complexity. BMC Bioinformatics. 2004;5 1:113. doi:10.1186/1471-2105-5-113.
68. Capella-Gutiérrez S, Silla-Martínez JM and Gabaldón T. trimAl: a tool for automated alignment trimming in large-scale phylogenetic analyses. Bioinformatics. 2009;25 15:1972-3. doi:10.1093/bioinformatics/btp348.
69. CatSequences developers: CatSequences. https://github.com/ChrisCreevey/catsequences (2025).
70. Darriba D, Posada D, Kozlov AM, Stamatakis A, Morel B and Flouri T. ModelTest-NG: A New and Scalable Tool for the Selection of DNA and Protein Evolutionary Models. Molecular Biology and Evolution. 2020;37 1:291-4. doi:10.1093/molbev/msz189.
71. Kozlov AM, Darriba D, Flouri T, Morel B and Stamatakis A. RAxML-NG: a fast, scalable and user-friendly tool for maximum likelihood phylogenetic inference. Bioinformatics. 2019;35 21:4453-5. doi:10.1093/bioinformatics/btz305.
72. Bouckaert R, Vaughan TG, Barido-Sottani J, Duchêne S, Fourment M, Gavryushkina A, et al. BEAST 2.5: An advanced software platform for Bayesian evolutionary analysis. PLOS Computational Biology. 2019;15 4:e1006650. doi:10.1371/journal.pcbi.1006650.
73. Strömberg CAE. Decoupled taxonomic radiation and ecological expansion of open-habitat grasses in the Cenozoic of North America. Proceedings of the National Academy of Sciences. 2005;102 34:11980-4. doi:10.1073/pnas.0505700102.
74. Prasad V, Strömberg CAE, Leaché AD, Samant B, Patnaik R, Tang L, et al. Late Cretaceous origin of the rice tribe provides evidence for early diversification in Poaceae. Nature Communications. 2011;2 1:480. doi:10.1038/ncomms1482.

75. Zhang X-Z, Zeng C-X, Ma P-F, Haevermans T, Zhang Y-X, Zhang L-N, et al. Multi-locus plastid phylogenetic biogeography supports the Asian hypothesis of the temperate woody bamboos (Poaceae: Bambusoideae). Molecular Phylogenetics and Evolution. 2016;96:118-29. doi:https://doi.org/10.1016/j.ympev.2015.11.025.
76. Rambaut A, Drummond AJ, Xie D, Baele G and Suchard MA. Posterior Summarization in Bayesian Phylogenetics Using Tracer 1.7. Systematic Biology. 2018;67 5:901-4. doi:10.1093/sysbio/syy032.
77. Mendes FK, Vanderpool D, Fulton B and Hahn MW. CAFE 5 models variation in evolutionary rates among gene families. Bioinformatics. 2021;36 22-23:5516-8. doi:10.1093/bioinformatics/btaa1022.
78. Wang Y, Tang H, DeBarry JD, Tan X, Li J, Wang X, et al. MCScanX: a toolkit for detection and evolutionary analysis of gene synteny and collinearity. Nucleic Acids Research. 2012;40 7:e49-e. doi:10.1093/nar/gkr1293.
79. Krzywinski M, Schein J, Birol I, Connors J, Gascoyne R, Horsman D, et al. Circos: an information aesthetic for comparative genomics. Genome Res. 2009;19 9:1639-45. doi:10.1101/gr.092759.109.
80. He W, Yang J, Jing Y, Xu L, Yu K and Fang X. NGenomeSyn: an easy-to-use and flexible tool for publication-ready visualization of syntenic relationships across multiple genomes. Bioinformatics. 2023;39 3:btad121. doi:10.1093/bioinformatics/btad121.
81. Tang SL, Xie JH and Cai JZ. The complete plastid genome of Thyrsostachys siamensis (Poaceae, Bambusoideae). Mitochondrial DNA B Resour. 2021;6 6:1781-3. doi:10.1080/23802359.2021.1934138.
82. Haibao Tang VK, Jingping Li: jcvi: JCVI utility libraries (v0.5.7). (2015).
83. Hamilton NE and Ferry M. ggtern: Ternary Diagrams Using ggplot2. Journal of Statistical Software, Code Snippets. 2018;87 3:1 - 17. doi:10.18637/jss.v087.c03.
84. Ramírez-González RH, Borrill P, Lang D, Harrington SA, Brinton J, Venturini L, et al. The transcriptional landscape of polyploid wheat. Science. 2018;361 6403:eaar6089. doi:10.1126/science.aar6089.
85. Hou Y, Gan J, Fan Z, Sun L, Garg V, Wang Y, et al. Haplotype-based pangenomes reveal genetic variations and climate adaptations in moso bamboo populations. Nature Communications. 2024;15 1:8085. doi:10.1038/s41467-024-52376-5.
86. Li A, Liu D, Wu J, Zhao X, Hao M, Geng S, et al. mRNA and small RNA transcriptomes reveal insights into dynamic homoeolog regulation of allopolyploid heterosis in nascent hexaploid wheat. The Plant Cell. 2014;26 5:1878-900.
87. Akhunova AR, Matniyazov RT, Liang H and Akhunov ED. Homoeolog-specific transcriptional bias in allopolyploid wheat. BMC genomics. 2010;11:1-16.
88. Yoo M, Szadkowski E and Wendel J. Homoeolog expression bias and expression level dominance in allopolyploid cotton. Heredity. 2013;110 2:171-80.
89. Wu J, Lin L, Xu M, Chen P, Liu D, Sun Q, et al. Homoeolog expression bias and expression level dominance in resynthesized allopolyploid Brassica napus. BMC Genomics. 2018;19 1:586. doi:10.1186/s12864-018-4966-5.

90. Combes MC, Cenci A, Baraille H, Bertrand B and Lashermes P. Homeologous gene expression in response to growing temperature in a recent Allopolyploid (Coffea arabica L.). J Hered. 2012;103 1:36-46. doi:10.1093/jhered/esr120.

91. Grover CE, Gallagher JP, Szadkowski EP, Yoo MJ, Flagel LE and Wendel JF. Homoeolog expression bias and expression level dominance in allopolyploids. New Phytologist. 2012;196 4:966-71. doi:https://doi.org/10.1111/j.1469-8137.2012.04365.x.

92. Naktang C, Khanbo S, Phadphon P, U-thoomporn S, Sangsakru D, Sonthirod C, et al. Supporting data for "Chromosome-level assemblies of two hexaploid bamboos Thyrsostachys oliveri and Thyrsostachys siamensis provide a foundation for functional and comparative genomics studies" GigaScience Database. 2025. https://doi.org/10.5524/102770.

## Figure titles and legends

**Figure 1** Genomic landscape and comparative collinearity of hexaploid *Thyrsostachys* and *Oryza sativa*. (A) Circos plot of the hexaploid *Thyrsostachys oliveri* assembly. The outermost ring shows chromosome ideograms for subgenome A (purple; 12 chromosomes), subgenome B (yellow; 12 chromosomes), and subgenome C (orange; 11 chromosomes). From the outermost data track inward:

1. Repeat content—proportion of bases covered by repetitive sequences in 500-kb windows.
2. Gene density—number of genes per 500-kb windows
3. GC content—percentage of G + C bases per 500-kb window.

(B) Circos plot of the *T. siamensis* assembly, formatted identically to panel A.

(C) Global collinearity between the 12 chromosomes of *O. sativa* and the A, B and C subgenomes of *T. oliveri* (left) and *T. siamensis* (right).

**Figure 2** Evolutionary analysis of the hexaploid *Thyrsostachys* genome. (A) Density plot showing pairwise four-fold synonymous (degenerative) third-codon transversion (4DTv) values between the indicated pairs of genomes. (B) Phylogenetic tree of 11 plant species, with the predicted divergence time shown as each branch. Adjacent pie charts show numbers of gene family expansion (shown in light green) and contraction (shown in dark green) among 11 species. The size and color of the circles represent the proportion of genes. (C) Flower-petal plot showing orthologous gene-family sharing among the 11 species (center circle) and species-specific gene families (individual petals). (D) Stacked bar chart of gene-family copy-number distributions across the 11 species. Bars are partitioned into categories (0, 1, 2, 3, 4, >4 copies); colors correspond to each copy-number class (legend at right).

**Figure 3** Homoeolog expression patterns in hexaploid *Thyrsostachys* genome. Ternary plots of relative expression contributions of the A, B and C subgenome homoeologs in (A) *T. oliveri* and (B) *T. siamensis*. Each point represents one expressed gene triad (sum TPM > 0.5) in either leaf (×) or root (○) tissue; its position reflects the proportion of total expression contributed by each homoeolog.

- Vertices (red, blue or green) indicate single-homoeolog dominance (A-, B- or C-dominant).
- Edges (orange, light-blue or light-green) indicate single-homoeolog suppression (A-, B- or C-suppressed).
- Center (grey) indicates balanced expression among all three homoeologs.

Percentages in the legend denote the fraction of expressed triads in each category.

**Supplementary Figure titles and legends**

**Supplementary Table S1.** Summary of sequencing data from stLFR and Hi-C platforms.

**Supplementary Table S2.** Comparative gene features across *Thyrsostachys* and 11 bamboo genomes.

**Supplementary Table S3.** Functional annotations of *T. oliveri* and *T. siamensis* protein-coding genes.

**Supplementary Table S4.** Noncoding RNA in the *T. oliveri* genome.

**Supplementary Table S5.** Noncoding RNA in the *T. siamensis* genome.

**Supplementary Table S6.** GO term enrichment analyses for gene families specific to *T. oliveri.*

**Supplementary Table S7.** GO term enrichment analyses for gene families specific to *T. siamensis.*

**Supplementary Table S8.** GO term enrichment analyses for gene families specific to *Thyrsostachys* crown node.

**Supplementary Table S9.** Percentage of syntenic triads assigned to the six homoeolog expression bias categories.

**Supplementary Table S10.** GO enrichment of homoeolog expression-bias categories in leaf and root of two *Thyrsostachys* bamboos.

**Supplementary Table S11.** Subgenome composition and distribution of 1:1:1 triads, duplets, and singletons in *Thyrsostachys*.

**Supplementary Table S12.** Assembly technology and key metrics across *Thyrsostachys* and PacBio long-read bamboo references.

**Supplementary Fig. S1** *Thyrsostachys oliveri* morphology. (A) Flowering branch with clustered spikelets; imbricate glumes and exserted anthers visible at anthesis. (B) Clump (habit) showing densely tufted, erect culms. (C) Culm node and adjacent internodes with branch complement; persistent leaf-sheath remnants present.

**Supplementary Fig. S2** *Thyrsostachys siamensis* morphology. (A) Clump (habit) with a tight tuft of erect culms. (B) Culm node and adjacent internodes showing the branch complement; persistent leaf-sheath rings at the nodes. (C) Leaf sheath and blade (narrow-lanceolate lamina).

**Supplementary Fig. S3** Example of manual Hi-C curation performed by Biomarker Technologies (BMK). (A) Pre-curation contact map viewed in CGAP with Positions 1–3 marked. The interaction between Positions 1 and 3 is stronger than between Positions 2 and 3. (B) Post-curation map after flipping the interval between Positions 1 and 2, restoring the expected near-diagonal cis signal. The interaction matrix was generated with LACHESIS and plotted in CGAP. Darker colors indicate stronger interaction intensities.

**Supplementary Fig. S4** Syntenic blocks are drawn between homologous chromosome pairs, illustrating strong collinearity that motivated selection of *D. sinicus* as the synteny framework (see Methods).

**Supplementary Fig. S5** Flow-cytometric estimation of nuclear DNA content in *Thyrsostachys* species. Nuclear DNA content was measured by propidium-iodide (PI) staining of nuclei isolated from fresh leaf tissue of (A) *T. siamensis* and (B) *T. oliveri*.

**Supplementary Fig. S6** Genome-survey k-mer distributions for hexaploidy *Thyrsostachys*.

**Supplementary Fig. S7** Genome-wide Hi-C contact map of hexaploid *Thyrsostachys oliveri*.

**Supplementary Fig. S8** GO annotation statistics for *T. oliveri*.

**Supplementary Fig. S9** GO annotation statistics for *T. siamensis*.

**Supplementary Fig. S10** Density plot showing pairwise synonymous substitution rate (Ks) values between the indicated pairs of genomes.

**Supplementary Fig. S11** Subgenome-specific clustering of single-copy orthologs across hexaploid bamboos

**Supplementary Fig. S12** GO term enrichment analyses for expanded gene families in *T. oliveri.* (A) Enriched biological processes among expanded gene families in *T. oliveri*. (B) Enriched cellular components among expanded gene families in *T. oliveri*. (C) Enriched molecular functions among expanded gene families in *T. oliveri*.

**Supplementary Fig. S13** GO term enrichment analyses for contracted gene families in *T. oliveri.* (A) Enriched biological processes among contracted gene families in *T. oliveri*. (B) Enriched molecular functions among contracted gene families in *T. oliveri*.

**Supplementary Fig. S14** GO term enrichment analyses for expanded gene families in *T. siamensis.* (A) Enriched biological processes among expanded gene families in

*T. siamensis*. (B) Enriched cellular components among expanded gene families in *T. siamensis*. (C) Enriched molecular functions among expanded gene families in *T. siamensis*.

**Supplementary Fig. S15** GO term enrichment analyses for contracted gene families in *T. siamensis*. (A) Enriched biological processes among contracted gene families in *T. siamensis*. (B) Enriched cellular components among contracted gene families in *T. siamensis*. (C) Enriched molecular functions among expanded gene families in *T. siamensis*.

Click here to access/download;Figure;Figure 1 Genomic landscape.png

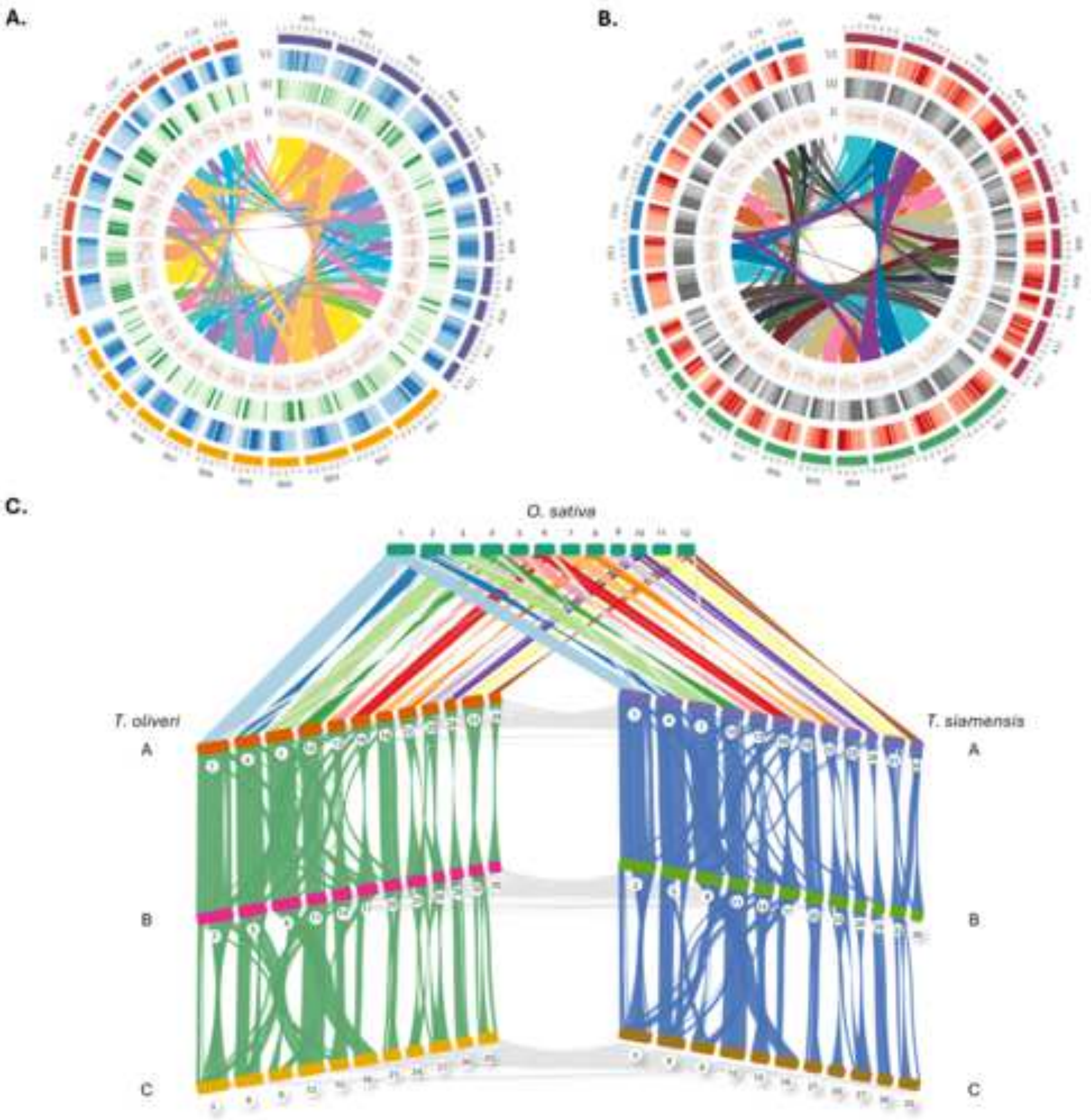

Click here to access/download;Figure;Figure 2 Evolutionary analysis.png

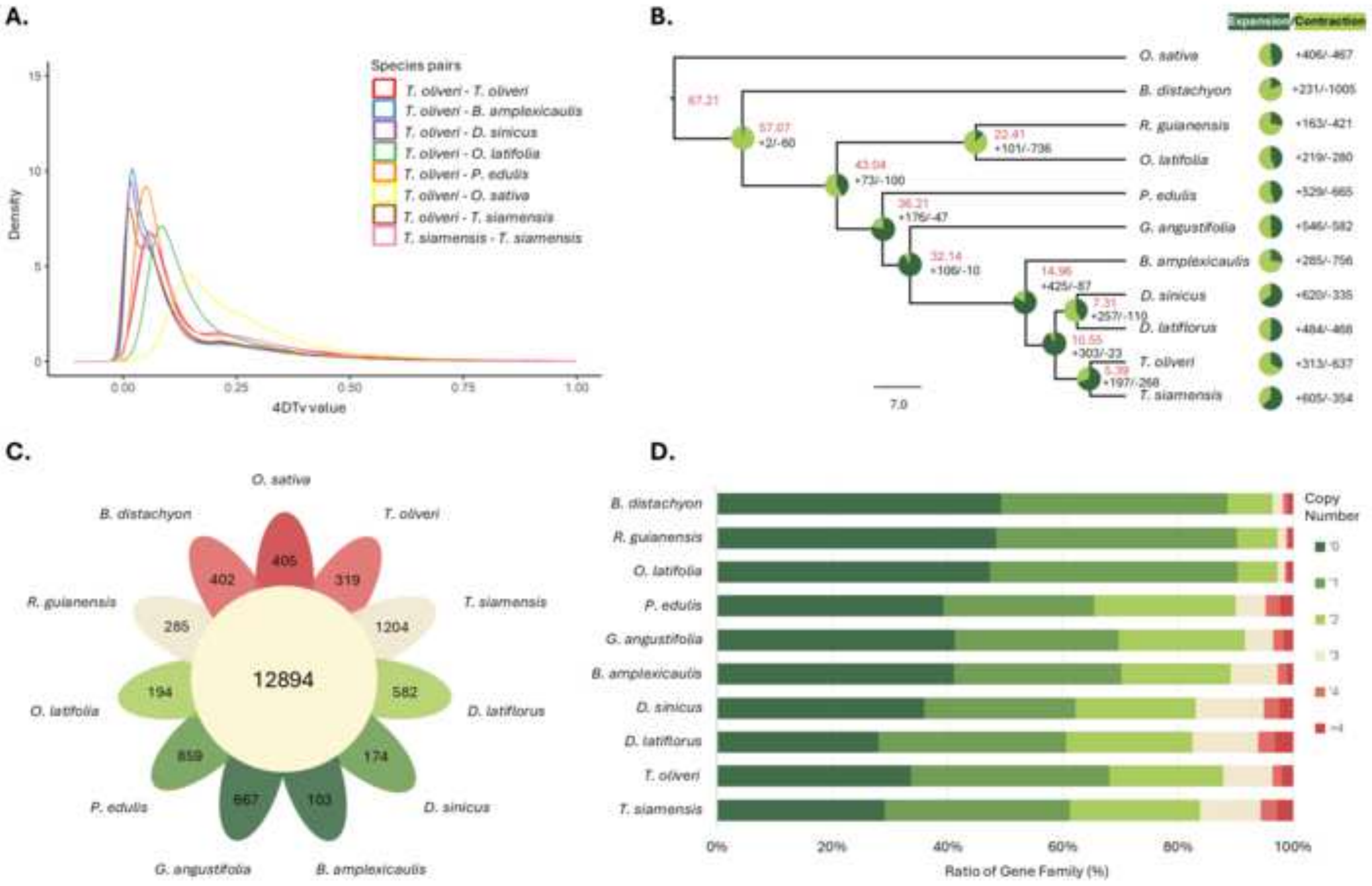

Click here to access/download;Figure;Figure 3 Homoeolog expression patterns.png

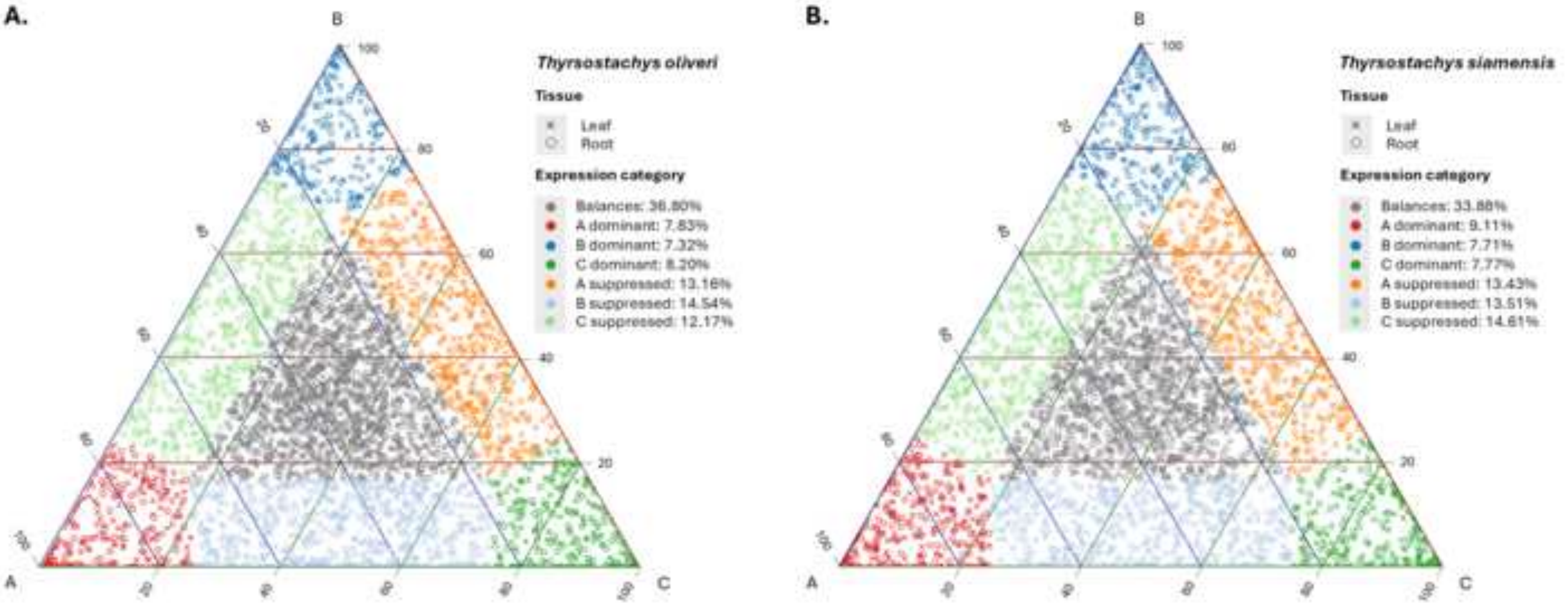

Click here to access/download
**Supplementary Material**
supplement_table_revision.xlsx

Click here to access/download
**Supplementary Material**
Supplementary Fig. S4.svg

Click here to access/download
**Supplementary Material**
Supplementary Fig. S1.PNG

Click here to access/download
**Supplementary Material**
Supplementary Fig. S2.PNG

Click here to access/download
**Supplementary Material**
Supplementary Fig. S3.png

Click here to access/download
**Supplementary Material**
Supplementary Fig. S5.PNG

Click here to access/download
**Supplementary Material**
Supplementary Fig. S6.png

Click here to access/download
**Supplementary Material**
Supplementary Fig. S8.PNG

Click here to access/download
**Supplementary Material**
Supplementary Fig. S9.png

Click here to access/download
**Supplementary Material**
Supplementary Fig. S10.png

Click here to access/download
**Supplementary Material**
Supplementary Fig. S11.tif

Click here to access/download
**Supplementary Material**
Supplementary Fig. S12.PNG

Click here to access/download
**Supplementary Material**
Supplementary Fig. S13.PNG

Click here to access/download
**Supplementary Material**
Supplementary Fig. S14.PNG

Click here to access/download
**Supplementary Material**
Supplementary Fig. S15.PNG

Click here to access/download
**Supplementary Material**
Reviewer reports_revision.docx
