# Supplementary material for: Chromosome-level assemblies of two hexaploid bamboos, Thyrsostachys oliveri and Thyrsostachys siamensis, provide a foundation for functional and comparative genomics studies
Source: Gigascience. 2025 Nov 17;14:giaf142. doi: 10.1093/gigascience/giaf142 (PMC12699669; doi:10.1093/gigascience/giaf142)
Supplement: giaf142_Authors_Response_To_Reviewer_Comments_Original_Submission [file giaf142_authors_response_to_reviewer_comments_original_submission.pdf]

Author's Response To Reviewer Comments

A Point-by-Point Response to Editor and Reviewer Comments.
Reviewer reports:

Reviewer #1: This manuscript by Naktang et al. presents the first chromosome-level genome assemblies for two economically important bamboo species, Thyrsostachys oliveri and T. siamensis. The generation of genomic resources for non-model, complex polyploid species like bamboo is a valuable endeavor for the plant genomics community.
However, despite the potential importance of the data, the manuscript in its current form suffers from several major deficiencies in its analytical depth, methodological reporting, and biological interpretation. The claims of producing "high-quality" genomes are not fully substantiated by state-of-the-art metrics and seem overstated in the current era of genomics. Furthermore, the comparative and functional analyses are largely superficial and fail to leverage this rich dataset to its full potential. The manuscript requires a substantial and thorough revision to address these fundamental issues before it can be considered for publication.

1. The claim of a "high-quality" assembly needs to be tempered. The assemblies are based on 150 bp short-read data (via stLFR), which inherently struggles to resolve complex repetitive regions. In the current era of Telomere-to-Telomere (T2T) genomics, assemblies derived from short reads cannot be unconditionally described as "high-quality" without significant caveats. The authors must discuss these limitations in the Discussion section.
Response: We thank the reviewer for this insightful comment. We agree and have revised the manuscript to temper our wording and to explicitly acknowledge short-read limitations. We now describe the assemblies as “chromosome-scale assemblies” rather than “high-quality.” We also added independent quality metrics to Supplementary Table S12: LAI = 7.75 / 7.10, Merqury QV = 55.85 / 52.5, and proteome-mode BUSCO completeness = 86.0% / 83.0% (embryophyta_odb10). We note in the Discussion that LAI < 10 indicates moderate repeat continuity and that, despite high per-base QV, long LTR-RTs and other complex repeats remain challenging for short reads in a hexaploid background. We have revised the Discussion section, and in lines 555–566 of the revised manuscript, we have added the following text:

“Compared with PacBio HiFi references, our stLFR+Hi-C assemblies achieve chromosome-scale, scaffold N50s and comparable BUSCO completeness (Supplementary Table S12) [22, 26, 78], while exhibiting lower repeat continuity (LAI) and base-level QV, as expected for short-read barcoded libraries. These trade-offs primarily affect long repetitive regions and do not preclude the gene-space and comparative analyses reported here. While Merqury QV indicates very high per-base accuracy, our LAI scores (<10) point to moderate repeat continuity, consistent with assemblies derived from 150-bp stLFR short reads. Long LTR retrotransposons and other complex or highly similar repeats are likely fragmented or collapsed, and some homoeologous regions in this hexaploid context may remain under-resolved. Future long-read (e.g., HiFi/ONT) and T2T scaffolding would further improve structural completeness and repeat resolution.”
2. The suite of genomic evaluation metrics is insufficient. Other standard measures, such as the LTR Assembly Index (LAI) for structural integrity and a base-level quality score (QV) from tools like Merqury, should be included to fully substantiate the quality claims.
Response: Thank you for this suggestion. We have added LAI and Merqury QV metrics to Supplementary Table S12 and now report them in the Results section (lines 355–361). Whole-genome LAI scores are 7.75 for T. oliveri and 7.10 for T. siamensis, indicating moderate repeat continuity. Merqury base-level quality scores are QV 55.85 and QV 52.5, respectively. We also tempered our wording to describe the assemblies as “chromosome-scale assemblies,” and, in the Discussion section (lines 557–566), added a brief note indicating that LAI < 10 reflects limited resolution of long LTR retrotransposons relative to long-read/T2T assemblies.

3. The authors report genome-mode BUSCO scores but omit the equally important proteome-mode BUSCO scores. The latter directly assesses the completeness of the final gene annotation set and is essential for evaluating the annotation quality.
Response: Thank you for the suggestion. We have added proteome-mode BUSCO (on the predicted protein

sets) alongside genome-mode, and we report both in the Results section (lines 354–356) and in Supplementary Table S12.

4. The reporting in Table 1 could be improved. The initial contig N50 is a more informative and standard metric for assessing baseline assembly fragmentation than the scaffold N75. It should be included for a direct comparison with the final scaffold N50.
Response: We revised Table 1 to include the initial contig N50 (pre-scaffold stLFR) alongside the final scaffold N50 (Hi-C for T. oliveri; RagTag for T. siamensis), enabling a direct baseline-to-final comparison. Specifically, contig N50s are 2,447,080 bp (T. oliveri) and 319,778 bp (T. siamensis), and final scaffold N50s are 22,175,826 bp and 19,454,152 bp, respectively. We retained Scaffold N75/L75/N90/L90 for completeness but now label all metrics by assembly stage (stLFR pre-scaffold vs Hi-C/RagTag final) to avoid ambiguity.

5. The manuscript lacks critical details regarding the manual curation and visualization of the Hi-C assembly. The authors state that "Manual inspection and Hi-C visualization tools were employed", but do not specify the criteria used for manual refinement or the names of the software. Furthermore, the methods should explicitly state how the Hi-C contact map in Supplementary Fig. S7 was generated and plotted. This information is essential for reproducibility.
Response: Thank you for this comment. The Methods (lines 168–175) have been revised to specify the software, the manual-refinement criterion, and how the contact map in Supplementary Fig. S3 was generated and plotted. Briefly, the genome-wide Hi-C contact matrix was generated with LACHESIS (Burton et al., 2013) by Biomarker Technologies (BMK). Manual inspection and limited adjustments were performed by BMK in CGAP (their in-house Hi-C visualization software), which displays higher interaction intensities as darker colors. As an example of the criterion used, when the interaction between Position 1 and Position 3 was stronger than that between Position 2 and Position 3 within a local block, the small interval between Positions 1 and 2 was flipped to restore the expected near-diagonal cis signal. Supplementary Fig. S3A shows the map before this flip and S3B shows the result after the flip. These details have been added to the Methods and the Supplementary figure legend for reproducibility.

6. The raw data appears to be inaccessible. The SRA Experiment accession SRX28190747 (corresponding to run SRR32915794 for T. oliveri) is listed as not public.
Response: Thank you for flagging this. The SRA records have now been set to public (as of 4 September 2025). The T. oliveri stLFR dataset SRX28190747 / SRR32915794 is visible and downloadable, and all other runs have likewise been released.

7. The comparative genomic analysis is disappointingly limited. While the authors cite the landmark Ma et al. (2024) paper and adopt its methodology for triad identification, they stop short of the most critical comparison. A fundamental, yet missing, analysis is to determine if the A, B, and C subgenomes in Thyrsostachys are orthologous to the core A, B, and C subgenomes of other hexaploid bamboos described in that study.
Response: We agree and now provide a subgenome-level orthology test across hexaploid bamboos. We compiled representative proteomes for Thyrsostachys (this study) and three hexaploids—Melocanna baccifera, Bonia amplexicaulis, and Dendrocalamus sinicus and ran OrthoFinder to infer orthogroups and gene trees. For single-copy orthogroups represented across species, we labeled tips by subgenome (A/B/C) and species and assessed whether sequences cluster by subgenome across taxa (i.e., A-of-Thyrsostachys groups with A-of-Melocanna, A-of-Bonia, A-of-Dendrocalamus; analogously for B and C). The resulting trees show consistent A-with-A, B-with-B, and C-with-C clustering (see Supplementary Fig. S11), supporting orthology between the Thyrsostachys subgenomes and the core A/B/C subgenomes reported previously. We have added this result to the Results section (lines 437–445).

8. The analysis focuses heavily on species-specific differences, neglecting the evolution of the Thyrsostachys genus as a lineage. At a minimum, the authors should identify and discuss the gene families that are specific to the genus (shared by both species but absent in other taxa) and those that expanded or contracted in the common ancestor of Thyrsostachys.
Response: We thank the reviewer for this insightful suggestion. In the revised manuscript, we have expanded our analysis to include gene family evolution at the Thyrsostachys crown node, thereby addressing lineage-level changes in addition to species-specific differences. Detailed explanations of the

results are provided in lines 458–472, with additional discussion in lines 612–616.

9. The whole-genome duplication (WGD) analysis, only relying on a 4DTv distribution plot. An analysis using synonymous substitution rates (Ks) derived from syntenic gene blocks would provide a clearer signal and is standard practice for a more robust WGD inference.
Response: We agree. We added a Ks-based analysis using syntenic anchor pairs from MCScanX (add_ka_and_ks_to_collinearity.pl). The Ks show clear bimodality with peaks at 0.150/0.516 in T. oliveri and 0.152/0.533 in T. siamensis, mirroring the 4DTv peaks (0.056/0.208 and 0.059/0.231, respectively). Inter-species Ks values recapitulate the same distance ranking as 4DTv (lowest between T. oliveri and T. siamensis), reinforcing the inference of a shared, relatively recent WGD. These additions have been incorporated into the Methods (lines 294–299), Results (lines 419–430, Fig. 2A; Supplementary Fig. S10;), and Discussion (lines 583–585) sections of the revised manuscript.

10. The biological context is underdeveloped. The manuscript provides only general statements about bamboo's utility but fails to frame the genomic analysis around any specific, unique biological or ecological traits of the Thyrsostachys genus.
Response: We appreciate your constructive feedback. In the revised Introduction, we now include specific details about the biological distinctiveness of Thyrsostachys oliveri and T. siamensis. These additions strengthen the rationale for conducting the genomic analyses presented in this study. This context has been incorporated into the Introduction section (lines 78–105).

11. The analysis of homeolog expression is merely descriptive and lacks biological interpretation. To provide meaningful insight, the authors must perform functional enrichment analyses (e.g., GO/KEGG) on the genes within each expression bias category (e.g., A-dominant, C-suppressed).
Response: We agree and have added a full functional-enrichment analysis stratified by homoeolog bias category. Specifically, we performed GO enrichment tests for all six categories (A/B/C-dominant; A/B/C-suppressed) in both tissues (leaf, root) of T. oliveri and T. siamensis. The new Results subsection “Functional enrichment of homoeolog bias categories” (lines 487–511) summarizes the biological interpretation, and Supplementary Table S10 provides full term lists and statistics.

12. The characterization of subgenome composition is incomplete. The authors should provide basic statistics, including the number of genes on each subgenome and the proportion that form 1:1:1 triads. Crucially, the manuscript must also quantify and discuss the genes that do not form triads (e.g., duplets, singlets, copy number variants), as the dynamics of gene loss and retention are central to polyploid evolution.
Response: We appreciate this suggestion and have added a comprehensive subgenome-composition analysis for both species (Results; Supplementary Table S11). We added a comprehensive subgenome-composition analysis for both species. Using the A–B–C subgenome assignments, we recorded, for each homoeolog correspondence set, whether copies were present in subgenomes A, B, and C and summarized these subgenome-presence classes as: triads (present in A, B, and C; 1:1:1), duplets (present in exactly two subgenomes), singletons (present in one), and other multi-copy configurations (including CNVs; any class in which a present subgenome contributes >1 copy). This context has been incorporated into the Results section, under "Subgenome composition and gene retention in Thyrsostachys" (lines 512–532).

13. The authors should ensure that all supplementary figures and tables are cited within the main body of the text to properly link the evidence to the narrative.
Response: We have carefully reviewed the main text and ensured that all supplementary figures and tables are now appropriately cited at relevant points in the manuscript.

14. A comprehensive check of(Akhunova et al. 2010) the reference list is required. Several entries appear to be incomplete or incorrectly formatted (e.g., references [34], [40], [52]), with inconsistencies in style throughout the bibliography. The entire reference list must be carefully reviewed and formatted according to the journal's guidelines.

Response: We have thoroughly reviewed and revised the entire reference list to ensure that all entries are complete and consistently formatted according to the journal’s guidelines.

Reviewer #2: This article provides a valuable resource by establishing reference genomes for two Thyrsostachys bamboo species, contributing significantly to the fields of bioinformatics, molecular biology, and evolutionary research. The study comprehensively investigated the genomics, comparative genomics, and comparative transcriptomics of Thyrsostachys oliveri and T. siamensis. Despite the strong data presented in this article, there is space for improvement in the depth of research. I have outlined some comments for the authors to address and revise accordingly.

（1） Line 35: In cases where the genus name is repeated, it is abbreviated the second time. Please check and revise other texts in your manuscript.
Response: We have carefully reviewed and revised the manuscript accordingly.

（2） Line 76-85: Can the authors emphasize the urgent need for conducting genome sequencing of Thyrsostachys oliveri and T. siamensis? Alternatively, describe the unique features that require research in genomics, comparative genomics, and comparative transcriptomics. Of course, this is not to question the work you all do.
Response: We appreciate the reviewer’s thoughtful suggestion. We have revised the Introduction to emphasize the urgent need for genome sequencing of Thyrsostachys oliveri and T. siamensis and to explicitly highlight their unique morphological and ecological traits, along with the current lack of genomic resources for this genus. This additional context has been incorporated into the Introduction section (lines 78–105).

（3） Line 89: Phyllostachys heterocycla and P. edulis are one species. The latter scientific name is accepted.
Response: We have corrected it.

（4） Line 90: I think authors should check more details about the genomes which had been published. Genomes of several species are not the first-time publised in ma et al. (2024). The article about the genomes of Bambusa odashimae, Raddia distichophylla, and Phyllostachys violascens should also be cited.
Response: We have cited the appropriate articles reporting the genomes of Bambusa odashimae, Raddia distichophylla, and Phyllostachys violascens as recommended.

（5） Line 121: "high-molecular-weight" should be replaced with the abbreviation "HMW".
Response: We have revised it.

（6） Line 241-242 and Line 396-397: If the genus names do not appear consecutively, please use the full name.
Response: We have revised it.

（7） Line335: In Table 1, I think the authors should provided LTR Assembly Index (LAI) for assessing genome assembly quality. The LAI can be obtained from LTR_retriever.
Response: Thank you for this suggestion. We have added LAI and Merqury QV metrics and now report them in the Results section (lines 355–361). Whole-genome LAI scores are 7.75 for T. oliveri and 7.10 for T. siamensis, indicating moderate repeat continuity. Merqury base-level quality scores are QV 55.85 and QV 52.5, respectively (Supplementary Table S12). We also tempered our wording to describe the assemblies as “chromosome-scale assemblies,” and added a brief Discussion note (lines 555–566) acknowledging that LAI < 10 reflects limited resolution of long LTR retrotransposons relative to long-read/T2T assemblies.

（8） Line 411: There are two Fig. S5s (Fig. S5A and Fig. S5B), but what your reference is Fig. S5. I think they should be revised as Fig. S5 nad Fig. S6, respectively. The comments are also for Fig. S6A and Fig. S6B.
Response: Thank you for pointing out the duplicated numbering. We have split the former panels into standalone supplementary figures and renumbered them as follows: former Fig. S5A → Fig. S12; former Fig. S5B → Fig. S13; former Fig. S6A → Fig. S14; former Fig. S6B → Fig. S15. All in-text citations and

figure captions have been updated accordingly.

（9） The font used in Supplementary Tables is rather strange and needs to be adjusted.
Response: We have revised the font style and formatting in all Supplementary Tables.

（10） In Disscussion section, when discussing the expansion and contraction of gene families, authors should consider specific biological characteristics of these two bamboo species or their unique evolutionary status, and may also integrate discussions with other bamboo species, rather than speaking in general terms.
Response: We thank the reviewer for this helpful suggestion. We have revised the Discussion section to explicitly link gene family expansions and contractions with the unique biological traits and evolutionary context of T. oliveri and T. siamensis. Furthermore, we have compared these patterns with gene family dynamics reported in other woody bamboo species (Discussion, lines 598–608).

（11） I think the authors should provide photos of Thyrsostachys oliveri and T. siamensis, whether they are included in the main text or in the supporting materials.

Response: Thank you for the suggestion. We have added photographs of Thyrsostachys oliveri and T. siamensis to the Supplementary Materials (Supplementary Fig. S1-S2).

Reviewer #3: This manuscripts presents chromosome-scale genomes of two economically significant Thai woody bamboos (Thyrsostachys oliveri and T. siamensis) using an integrated approach of single-tube long-fragment read (stLFR) sequencing and Hi-C scaffolding. The authors performed comprehensive assemble, gene annotation, evolutionary analyses, and preliminary investigation of homoeolog expression patterns. The release of these genomes, combined with previously published bamboo genomes, will facilitate deeper exploration of bamboo-specific biological traits. I recommend acceptance after minor revisions, with the following suggestions:

Lines 282-284: Clarify why D. sinicus was selected as the reference framework: Is this solely due to its allele-aware chromosome-level quality, or also because of its closer phylogenetic affinity with Thyrsostachys? Provide supporting evidence for this choice.

Response: We selected D. sinicus as the primary reference for three reasons:
1. Published plastome phylogenies place Thyrsostachys within Bambusinae and resolve T. siamensis as sister to Dendrocalamus birmanicus, indicating close affinity between the genera (consistent with our Fig. 2) [DOI: 10.1080/23802359.2021.1934138]
2. Assembly quality and ploidy match. D. sinicus is one of the hexaploid woody bamboos with a chromosome-level genome assembled using long reads plus Hi-C; hexaploid species in that study were consistently anchored to 35 pseudo-chromosomes. This provides a like-for-like framework for subgenome-aware synteny. [DOI: 10.1038/s41588-024-01683-0]
3. Genome-scale compatibility. Reported hexaploid bamboo genome sizes average ~1.12 Gb, matching our Thyrsostachys assemblies (~1.0–1.14 Gb), which facilitates 1:1 collinearity assessment. [DOI: 10.1038/s41588-024-01683-0]
We also validated this choice empirically: whole-genome dot plots between our Thyrsostachys assemblies and D. sinicus show extensive 1:1 collinearity with limited large-scale rearrangements (now Supplementary Fig. S4). Together, the close phylogenetic relationship, chromosome-scale hexaploid reference, and syntenic congruence justify using D. sinicus as the reference framework.
We added the following sentence to the Methods section (lines 305–308) for clarity: “We selected D. sinicus as the synteny framework owing to its close placement to Thyrsostachys in published plastome phylogenies [74], together with its chromosome-level hexaploid assembly and strong collinearity with our genomes (Supplementary Fig. S4).”

About stLFR Methodology: Given that most published bamboo genomes used PacBio long-read technology, please directly compare key assembly metrics (e.g., N50, BUSCO completeness) between your stLFR-based assemblies and phylogenetically proximate species from published bamboo genomes. This will explicitly demonstrate the competitiveness of stLFR with PacBio technology.

Response: We added Supplementary Table S12 benchmarking our chromosome-scale stLFR assemblies against representative PacBio genomes (the Phyllostachys edulis HiFi pangenome, P. violascens 'Prevernalis', and Dendrocalamus latiflorus). The table reports contiguity (contig/scaffold N50), anchoring, BUSCO (genome and proteome mode), LAI, and QV. In the Discussion (lines 555–566), we summarize the comparison and explicitly acknowledge technology-driven differences.

Lines 337-345: Strengthen the comparative analysis based on gene features (e.g., gene length, exon/intron length, GC content….) with the 11 recently published bamboo genomes, highlighting conserved and divergent features in T. oliveri and T. siamensis.

Response: Thank you for the suggestion. We have added a side-by-side comparison of gene features for T. oliveri and T. siamensis against the 11 recently published bamboo genomes in the Results section (lines 372–376), with the updated Supplementary Table S2 including species, gene count, average gene length, average exon number, median exon length, and average intron length. This highlights both conserved and divergent patterns:
Conserved: median exon size is tightly clustered across bamboos (~124–158 bp), and most species including T. oliveri have ~5 exons per gene.
Divergent in Thyrsostachys: average gene length is shorter in T. oliveri/T. siamensis (2.86/2.67 kb) than in other 11 published bamboo genome (~4.0–5.0 kb), and average intron length is also shorter (436/492 bp vs ~600–770 bp). T. siamensis has fewer exons per gene (4.27) than most bamboos (~5).

Lines 416-429: To reinforce the observed subgenome-specific expression biases, include one concrete example (e.g., evolutionarily significant gene) demonstrating subgenome-specific expression biases differing between species.
Response: We expanded the functional interpretation with GO enrichment across the six bias categories (Results: "Functional enrichment of homoeolog bias categories"). To provide the requested concrete example, we now highlight a mannose-6-phosphate isomerase (PMI; GO:0004476) triad that shows species-specific subgenome bias B-dominant in T. oliveri leaves but not B-dominant in T. siamensis. Full term lists and statistics are provided in Supplementary Table S10.

Line 210: Please updated the outdated hyperlink to the currently accessible version: https://doi.org/10.6084/m9.figshare.24411913.v3
Response: Thank you for the suggestion. We have updated Line 210 to the versioned DOI and now cite the dataset as: "Figshare (DOI: 10.6084/m9.figshare.24411913.v3)."

About data Accessibility (Lines 161-168 & 199-217): While raw reads have been deposited, gene annotation files (GFF format) should be submitted alongside the genome assemblies to ensure full data utility.
Response: We have deposited the complete annotation sets in GigaDB alongside both assemblies, including GFF3, protein (.faa), CDS (.fna), functional-annotation tables, and a README with checksums.

Editorial Manager®

Cookie Preference Center

We use cookies which are necessary to make our site work. We may also use additional cookies to analyze, improve and personalize our content and your digital experience. For more information, see our Cookie Policy.

You may choose not to allow some types of cookies. However, blocking some types may impact your experience of our site and the services we are able to offer. See the different category headings below to find out more or change your settings.

Allow all

Manage Consent Preferences

Strictly Necessary Cookies

Always active

These cookies are necessary for the website to function and cannot be switched off in our systems. They are usually only set in response to actions made by you which amount to a request for services, such as setting your privacy preferences, logging in or filling in forms. You can set your browser to block or alert you about these cookies, but some parts of the site will not then work.

Functional Cookies

These cookies enable the website to provide enhanced functionality and personalisation. They may be set by us or by third party providers whose services we have added to our pages. If you do not allow these cookies then some or all of these services may not function properly.

Performance Cookies

These cookies allow us to count visits and traffic sources so we can measure and improve the performance of our site. They help us to know which pages are the most and least popular and see how visitors move around the site.

Targeting Cookies

These cookies may be set through our site by our advertising partners. They may be used by those companies to build a profile of your interests and show you relevant adverts on other sites. If you do not allow these cookies, you will experience less targeted advertising.

Cookie List

Clear

Apply Cancel
Consent Leg.Interest

Confirm my choices

Powered by onetrust
