# Supplementary material for: Chromosome-level assemblies of two hexaploid bamboos, Thyrsostachys oliveri and Thyrsostachys siamensis, provide a foundation for functional and comparative genomics studies
Source: Gigascience. 2025 Nov 17;14:giaf142. doi: 10.1093/gigascience/giaf142 (PMC12699669; doi:10.1093/gigascience/giaf142)
Supplement: giaf142_Reviewer_1_Report_Original_Submission — Hansheng Zhao -- 7/31/2025 [file giaf142_reviewer_1_report_original_submission.pdf]

Reviewer Report

Title: Chromosome-level assemblies of two hexaploid bamboos Thyrsostachys oliveri and Thyrsostachys siamensis provide a foundation for functional and comparative genomics studies

Version: Original Submission **Date:** 7/31/2025

Reviewer name: Hansheng Zhao

Reviewer Comments to Author:

This manuscript by Naktang et al. presents the first chromosome-level genome assemblies for two economically important bamboo species, Thyrsostachys oliveri and T. siamensis. The generation of genomic resources for non-model, complex polyploid species like bamboo is a valuable endeavor for the plant genomics community.

However, despite the potential importance of the data, the manuscript in its current form suffers from several major deficiencies in its analytical depth, methodological reporting, and biological interpretation. The claims of producing "high-quality" genomes are not fully substantiated by state-of-the-art metrics and seem overstated in the current era of genomics. Furthermore, the comparative and functional analyses are largely superficial and fail to leverage this rich dataset to its full potential. The manuscript requires a substantial and thorough revision to address these fundamental issues before it can be considered for publication.

1. The claim of a "high-quality" assembly needs to be tempered. The assemblies are based on 150 bp short-read data (via stLFR), which inherently struggles to resolve complex repetitive regions. In the current era of Telomere-to-Telomere (T2T) genomics, assemblies derived from short reads cannot be unconditionally described as "high-quality" without significant caveats. The authors must discuss these limitations in the Discussion section.
2. The suite of genomic evaluation metrics is insufficient. Other standard measures, such as the LTR Assembly Index (LAI) for structural integrity and a base-level quality score (QV) from tools like Merqury, should be included to fully substantiate the quality claims.
3. The authors report genome-mode BUSCO scores but omit the equally important proteome-mode BUSCO scores. The latter directly assesses the completeness of the final gene annotation set and is essential for evaluating the annotation quality.
4. The reporting in Table 1 could be improved. The initial contig N50 is a more informative and standard metric for assessing baseline assembly fragmentation than the scaffold N75. It should be included for a direct comparison with the final scaffold N50.
5. The manuscript lacks critical details regarding the manual curation and visualization of the Hi-C assembly. The authors state that "Manual inspection and Hi-C visualization tools were employed", but do not specify the criteria used for manual refinement or the names of the software. Furthermore, the methods should explicitly state how the Hi-C contact map in Supplementary Fig. S7 was generated and plotted. This information is essential for reproducibility.
6. The raw data appears to be inaccessible. The SRA Experiment accession SRX28190747 (corresponding to run SRR32915794 for T. oliveri) is listed as not public.

7. The comparative genomic analysis is disappointingly limited. While the authors cite the landmark Ma et al. (2024) paper and adopt its methodology for triad identification, they stop short of the most critical comparison. A fundamental, yet missing, analysis is to determine if the A, B, and C subgenomes in Thyrsostachys are orthologous to the core A, B, and C subgenomes of other hexaploid bamboos described in that study.
8. The analysis focuses heavily on species-specific differences, neglecting the evolution of the Thyrsostachys genus as a lineage. At a minimum, the authors should identify and discuss the gene families that are specific to the genus (shared by both species but absent in other taxa) and those that expanded or contracted in the common ancestor of Thyrsostachys.
9. The whole-genome duplication (WGD) analysis, only relying on a 4DTv distribution plot. An analysis using synonymous substitution rates (Ks) derived from syntenic gene blocks would provide a clearer signal and is standard practice for a more robust WGD inference.
10. The biological context is underdeveloped. The manuscript provides only general statements about bamboo's utility but fails to frame the genomic analysis around any specific, unique biological or ecological traits of the Thyrsostachys genus.
11. The analysis of homeolog expression is merely descriptive and lacks biological interpretation. To provide meaningful insight, the authors must perform functional enrichment analyses (e.g., GO/KEGG) on the genes within each expression bias category (e.g., A-dominant, C-suppressed).
12. The characterization of subgenome composition is incomplete. The authors should provide basic statistics, including the number of genes on each subgenome and the proportion that form 1:1:1 triads. Crucially, the manuscript must also quantify and discuss the genes that do not form triads (e.g., duplets, singlets, copy number variants), as the dynamics of gene loss and retention are central to polyploid evolution.
13. The authors should ensure that all supplementary figures and tables are cited within the main body of the text to properly link the evidence to the narrative.
14. A comprehensive check of the reference list is required. Several entries appear to be incomplete or incorrectly formatted (e.g., references [34], [40], [52]), with inconsistencies in style throughout the bibliography. The entire reference list must be carefully reviewed and formatted according to the journal's guidelines.

Methods

Are the methods appropriate to the aims of the study, are they well described, and are necessary controls included? Choose an item.

Conclusions

Are the conclusions adequately supported by the data shown? Choose an item.

Reporting Standards

Does the manuscript adhere to the journal's guidelines on [minimum standards of reporting?]() Choose an item.

Choose an item.

Statistics

Are you able to assess all statistics in the manuscript, including the appropriateness of statistical tests used? Choose an item.

Quality of Written English

Please indicate the quality of language in the manuscript: Choose an item.

Declaration of Competing Interests

Please complete a declaration of competing interests, considering the following questions:

- Have you in the past five years received reimbursements, fees, funding, or salary from an organisation that may in any way gain or lose financially from the publication of this manuscript, either now or in the future?
- Do you hold any stocks or shares in an organisation that may in any way gain or lose financially from the publication of this manuscript, either now or in the future?
- Do you hold or are you currently applying for any patents relating to the content of the manuscript?
- Have you received reimbursements, fees, funding, or salary from an organization that holds or has applied for patents relating to the content of the manuscript?
- Do you have any other financial competing interests?
- Do you have any non-financial competing interests in relation to this paper?

If you can answer no to all of the above, write 'I declare that I have no competing interests' below. If your reply is yes to any, please give details below.

All No.

I agree to the open peer review policy of the journal. I understand that my name will be included on my report to the authors and, if the manuscript is accepted for publication, my named report including any attachments I upload will be posted on the website along with the authors' responses. I agree for my report to be made available under an Open Access Creative Commons CC-BY license (http://creativecommons.org/licenses/by/4.0/). I understand that any comments which I do not wish to be included in my named report can be included as confidential comments to the editors, which will not be published.

Choose an item.

To further support our reviewers, we have joined with Publons, where you can gain additional credit to further highlight your hard work (see: https://publons.com/journal/530/gigascience). On publication of this paper, your review will be automatically added to Publons, you can then choose whether or not to claim your Publons credit. I understand this statement.

Yes Choose an item.
