# Supplementary material for: Chromosome-level assemblies of two hexaploid bamboos, Thyrsostachys oliveri and Thyrsostachys siamensis, provide a foundation for functional and comparative genomics studies
Source: Gigascience. 2025 Nov 17;14:giaf142. doi: 10.1093/gigascience/giaf142 (PMC12699669; doi:10.1093/gigascience/giaf142)
Supplement: giaf142_Reviewer_2_Report_Original_Submission — Mingbing Zhou -- 8/2/2025 [file giaf142_reviewer_2_report_original_submission.pdf]

Reviewer Report

Title: Chromosome-level assemblies of two hexaploid bamboos Thyrsostachys oliveri and Thyrsostachys siamensis provide a foundation for functional and comparative genomics studies

Version: Original Submission **Date:** 8/2/2025

Reviewer name: Mingbing Zhou

Reviewer Comments to Author:

This article provides a valuable resource by establishing reference genomes for two Thyrsostachys bamboo species, contributing significantly to the fields of bioinformatics, molecular biology, and evolutionary research. The study comprehensively investigated the genomics, comparative genomics, and comparative transcriptomics of Thyrsostachys oliveri and T. siamensis. Despite the strong data presented in this article, there is space for improvement in the depth of research. I have outlined some comments for the authors to address and revise accordingly.

ï¼ˆ1ï¼‰ Line 35: In cases where the genus name is repeated, it is abbreviated the second time. Please check and revise other texts in your manuscript.

ï¼ˆ2ï¼‰ Line 76-85:Can the authors emphasize the urgent need for conducting genome sequencing of Thyrsostachys oliveri and T. siamensis? Alternatively, describe the unique features that require research in genomics, comparative genomics, and comparative transcriptomics. Of course, this is not to question the work you all do.

ï¼ˆ3ï¼‰ Line 89: Phyllostachys heterocycla and P. edulis are one species. The latter scientific name is accepted.

ï¼ˆ4ï¼‰ Line 90: I think authors should check more details about the genomes which had been published. Genomes of several species are not the first-time publised in ma et al. (2024). The article about the genomes of Bambusa odashimae, Raddia distichophylla, and Phyllostachys violascens should also be cited.

ï¼ˆ5ï¼‰ Line 121: "high-molecular-weight" should be replaced with the abbreviation "HMW".

ï¼ˆ6ï¼‰ Line 241-242 and Line 396-397: If the genus names do not appear consecutively, please use the full name.

ï¼ˆ7ï¼‰ Line335: In Table 1, I think the authors should provided LTR Assembly Index (LAI) for assessing genome assembly quality. The LAI can be obtained from LTR_retriever.

ï¼ˆ8ï¼‰ Line 411: There are two Fig. S5s (Fig. S5A and Fig. S5B), but what your reference is Fig. S5. I think they should be revised as Fig. S5 nad Fig. S6, respectively. The comments are also for Fig. S6A and Fig. S6B.

ï¼ˆ9ï¼‰ The font used in Supplementary Tables is rather strange and needs to be adjusted.

ï¼ˆ10ï¼‰ In Disscussion section, when discussing the expansion and contraction of gene families, authors should consider specific biological characteristics of these two bamboo species or their unique evolutionary status, and may also integrate discussions with other bamboo species, rather than speaking in general terms.

ï¼ˆ11ï¼‰ I think the authors should provide photos of Thyrsostachys oliveri and T. siamensis, whether

they are included in the main text or in the supporting materials.

Methods

Are the methods appropriate to the aims of the study, are they well described, and are necessary controls included? Choose an item.

Conclusions

Are the conclusions adequately supported by the data shown? Choose an item.

Reporting Standards

Does the manuscript adhere to the journal's guidelines on minimum standards of reporting? Choose an item.

Choose an item.

Statistics

Are you able to assess all statistics in the manuscript, including the appropriateness of statistical tests used? Choose an item.

Quality of Written English

Please indicate the quality of language in the manuscript: Choose an item.

Declaration of Competing Interests

Please complete a declaration of competing interests, considering the following questions:

- Have you in the past five years received reimbursements, fees, funding, or salary from an organisation that may in any way gain or lose financially from the publication of this manuscript, either now or in the future?
- Do you hold any stocks or shares in an organisation that may in any way gain or lose financially from the publication of this manuscript, either now or in the future?
- Do you hold or are you currently applying for any patents relating to the content of the manuscript?
- Have you received reimbursements, fees, funding, or salary from an organization that holds or has applied for patents relating to the content of the manuscript?
- Do you have any other financial competing interests?
- Do you have any non-financial competing interests in relation to this paper?

If you can answer no to all of the above, write 'I declare that I have no competing interests' below. If your reply is yes to any, please give details below.

I declare that I have no competing interests

I agree to the open peer review policy of the journal. I understand that my name will be included on my report to the authors and, if the manuscript is accepted for publication, my named report including any attachments I upload will be posted on the website along with the authors' responses. I agree for my report to be made available under an Open Access Creative Commons CC-BY license (http://creativecommons.org/licenses/by/4.0/). I understand that any comments which I do not wish to be included in my named report can be included as confidential comments to the editors, which will not be published.

Choose an item.

To further support our reviewers, we have joined with Publons, where you can gain additional credit to further highlight your hard work (see: https://publons.com/journal/530/gigascience). On publication of this paper, your review will be automatically added to Publons, you can then choose whether or not to claim your Publons credit. I understand this statement.

Yes Choose an item.
