# Supplementary material for: Chromosome-level assemblies of two hexaploid bamboos, Thyrsostachys oliveri and Thyrsostachys siamensis, provide a foundation for functional and comparative genomics studies
Source: Gigascience. 2025 Nov 17;14:giaf142. doi: 10.1093/gigascience/giaf142 (PMC12699669; doi:10.1093/gigascience/giaf142)
Supplement: giaf142_Reviewer_3_Report_Original_Submission — Lianfeng Gu -- 8/5/2025 [file giaf142_reviewer_3_report_original_submission.pdf]

Reviewer Report

Title: Chromosome-level assemblies of two hexaploid bamboos Thyrsostachys oliveri and Thyrsostachys siamensis provide a foundation for functional and comparative genomics studies

Version: Original Submission **Date:** 8/5/2025

Reviewer name: Lianfeng Gu

Reviewer Comments to Author:

This manuscripts presents chromosome-scale genomes of two economically significant Thai woody bamboos (Thyrsostachys oliveri and T. siamensis) using an integrated approach of single-tube long-fragment read (stLFR) sequencing and Hi-C scaffolding. The authors performed comprehensive assemble, gene annotation, evolutionary analyses, and preliminary investigation of homoeolog expression patterns. The release of these genomes, combined with previously published bamboo genomes, will facilitate deeper exploration of bamboo-specific biological traits. I recommend acceptance after minor revisions, with the following suggestions:
Lines 282-284: Clarify why D. sinicus was selected as the reference framework: Is this solely due to its allele-aware chromosome-level quality, or also because of its closer phylogenetic affinity with Thyrsostachys? Provide supporting evidence for this choice.
About stLFR Methodology: Given that most published bamboo genomes used PacBio long-read technology, please directly compare key assembly metrics (e.g., N50, BUSCO completeness) between your stLFR-based assemblies and phylogenetically proximate species from published bamboo genomes. This will explicitly demonstrate the competitiveness of stLFR with PacBio technology.
Lines 337-345: Strengthen the comparative analysis based on gene features (e.g., gene length, exon/intron length, GC content….) with the 11 recently published bamboo genomes, highlighting conserved and divergent features in T. oliveri and T. siamensis.
Lines 416-429: To reinforce the observed subgenome-specific expression biases, include one concrete example (e.g., evolutionarily significant gene) demonstrating subgenome-specific expression biases differing between species.
Line 210: Please updated the outdated hyperlink to the currently accessible version: https://doi.org/10.6084/m9.figshare.24411913.v3
About data Accessibility (Lines 161-168 & 199-217): While raw reads have been deposited, gene annotation files (GFF format) should be submitted alongside the genome assemblies to ensure full data utility.

Methods

Are the methods appropriate to the aims of the study, are they well described, and are necessary controls included? Choose an item.

Conclusions

Are the conclusions adequately supported by the data shown? Choose an item.

Reporting Standards

Does the manuscript adhere to the journal's guidelines on minimum standards of reporting? Choose an item.

Choose an item.

Statistics

Are you able to assess all statistics in the manuscript, including the appropriateness of statistical tests used? Choose an item.

Quality of Written English

Please indicate the quality of language in the manuscript: Choose an item.

Declaration of Competing Interests

Please complete a declaration of competing interests, considering the following questions:

- Have you in the past five years received reimbursements, fees, funding, or salary from an organisation that may in any way gain or lose financially from the publication of this manuscript, either now or in the future?
- Do you hold any stocks or shares in an organisation that may in any way gain or lose financially from the publication of this manuscript, either now or in the future?
- Do you hold or are you currently applying for any patents relating to the content of the manuscript?
- Have you received reimbursements, fees, funding, or salary from an organization that holds or has applied for patents relating to the content of the manuscript?
- Do you have any other financial competing interests?
- Do you have any non-financial competing interests in relation to this paper?

If you can answer no to all of the above, write 'I declare that I have no competing interests' below. If your reply is yes to any, please give details below.

I declare that I have no competing interests

I agree to the open peer review policy of the journal. I understand that my name will be included on my report to the authors and, if the manuscript is accepted for publication, my named report including any attachments I upload will be posted on the website along with the authors' responses. I agree for my report to be made available under an Open Access Creative Commons CC-BY license (http://creativecommons.org/licenses/by/4.0/). I understand that any comments which I do not wish to

be included in my named report can be included as confidential comments to the editors, which will not be published.

Choose an item.

To further support our reviewers, we have joined with Publons, where you can gain additional credit to further highlight your hard work (see: https://publons.com/journal/530/gigascience). On publication of this paper, your review will be automatically added to Publons, you can then choose whether or not to claim your Publons credit. I understand this statement.

Yes Choose an item.
